# Group B streptococcal disease in infants 0–3 months in The Netherlands, 1987–2023: a nationwide genomic and epidemiological surveillance study

**DOI:** 10.1016/j.lanepe.2026.101711

**Published:** 2026-05-12

**Authors:** Marleen A. Groenveld, Dorota Jamrozy, Douwe H. Visser, Matthijs C. Brouwer, Diederik van de Beek, Merijn W. Bijlsma, Nina M. van Sorge, NOGBS study group, Ron van Beek, Vincent Bekker, Maartje van den Berg, Geert Jan Blok, Mijke Breukels, Alwin F.J. Brouwer, Renske J.P.M. Cornelisse-van Vugt, Luçan C. Delemarre, Anouk Dings, Rienus A. Doedens, Stefan M. van Dorth, Hester M. Havers, Jojanneke Heidema, Marieke A.C. Hemels, Maartje E.N. van den Heuvel, Marlies A. van Houten, Monique A.M. Jacobs, Arieke Janse, Miranda de Jong, Anton H. van Kaam, Ageeth Kaspers, Merel N. van Kassel, Anne A.M.W. van Kempen, Kristine Klúčovská, Karen Korbeek, René F. Kornelisse, Anke G. Kuijpers, Taco W. Kuijpers, Elizabeth van Leeuwen, Jeannette von Lindern, Carmen Lorente Flores, Karen Van Mechelen, Clemens B. Meijssen, Jeroen Noordzij, Annemarie Oudshoorn, Frans B. Plötz, Marjolijn Quaak, Maaike van Rossem, Maarten Rijpert, Machteld A.G. van Scherpenzeel-de Vries, Irene Schiering, George Shabo, Linde Snoek, Nina M. van Sorge, Jacqueline U.M. Termote, Gerdien A. Tramper-Stranders, Mirjam van Veen, Marlies Vermaas, Marjoke Verweij, Douwe H. Visser, Wouter J. de Waal, Anne-Marie van Wermeskerken, Janneke F. Wilms, Tom F.W. Wolfs, Angela C.M. van Zijl

**Affiliations:** a Department of Neurology, Amsterdam University Medical Centre, University of Amsterdam, Amsterdam, the Netherlands; b Parasites and Microbes, Wellcome Sanger Institute, Wellcome Genome Campus, Hinxton, United Kingdom; c Department of Neonatology, Emma Children's Hospital, Amsterdam University Medical Centre, University of Amsterdam, Amsterdam, the Netherlands; d Department of Paediatrics, Emma Children's Hospital, Amsterdam University Medical Centre, University of Amsterdam, Amsterdam, the Netherlands; e Department of Medical Microbiology and Infection Prevention, Netherlands Reference Laboratory for Bacterial Meningitis, Amsterdam University Medical Centre, University of Amsterdam, Amsterdam, the Netherlands

**Keywords:** Bacterial meningitis, Group B Streptococcus, Infants, Neonates, Early-onset disease, Late-onset disease, Maternal vaccination

## Abstract

**Background:**

Group B *Streptococcus* (GBS) is the leading cause of sepsis and meningitis in infants. The molecular epidemiology and potential vaccine coverage of GBS disease in infants was assessed in the Netherlands over a 37-year period.

**Methods:**

This nationwide observational study identified infants aged 0–89 days with GBS culture-positive sepsis or meningitis between July 1987 and June 2024, through Dutch surveillance. Serotype was determined by latex agglutination. Whole-genome sequencing determined the clonal complex (CC) using multi-locus sequence typing (MLST) and identified virulence factors associated with meningitis. Strain coverage by the maternal vaccines GBS6 and GBS-AlpN were analysed.

**Findings:**

2212 episodes were identified; 1307 (59%) early onset disease (0–6 days) and 905 (41%) late onset disease (7–89 days). Mean annual incidence was 0·33 per 1000 live births and significantly increased from 0·19 in 1987 to 0·57 in 2023 due to an increase in sepsis cases (p < 0·0001). Serotype data was available for 2163 (98%) of 2212 isolates, of which serotype III was most common (1316/2163; 61%), followed by Ia (383/2163; 18%) and II (123/2163; 6%). MLST data was available for 1723 (78%) isolates; CC17 was most common (705/1723; 41%). CC17 increased from 29% (90/308) in 1987–1996 to 49% (262/538) in 2014–2023 (p < 0·0001). 97% (2095/2163) of cases would be covered by the GBS6 vaccine, and 99% (1709/1723) by GBS-AlpN.

**Interpretation:**

GBS disease is still increasing in the Netherlands. The GBS6 and GBS-AlpN vaccines would potentially prevent >96% of cases.

**Funding:**

Netherlands Organisation for Health Research and Development (ZonMW) and ItsME foundation.

**Research in context**

**Evidence before this study**

We searched PubMed up to August 2025 using the terms “*Streptococcus agalactiae*”, “group B streptococcus”, and “molecular epidemiology” to identify epidemiological studies on invasive group B streptococcal (GBS) disease in young infants. Previous work from several high-income countries has reported a rising incidence of invasive GBS disease, alongside variation in national approaches to intrapartum antibiotic prophylaxis. In the Netherlands, studies reported a sustained increase in GBS disease incidence up to 2015. Maternal immunisation is a promising new strategy to address the rising global burden of GBS disease in young infants, and two candidate vaccine types are presently undergoing clinical evaluation. Prior molecular epidemiology studies have indicated that the predominant serotypes and sequence types causing neonatal disease fall within the anticipated coverage of these candidate vaccines.

**Added value of this study**

This study provides the first national data on the incidence of invasive GBS disease in the Netherlands over the past decade and evaluates the anticipated coverage of two maternal GBS vaccine candidates. We show that the incidence of invasive GBS disease in young infants has continued to rise through 2024, driven primarily by an increase in sepsis cases. Our data indicate that the current Dutch risk-based intrapartum antibiotic prophylaxis strategy does not adequately prevent early-onset GBS disease. The epidemiology remains dominated by clonal complex 17 and serotype III. We evaluated virulence factors previously linked to GBS meningitis and found that HvgA, Srr2, FbsB, and the combination of PI-1 and PI-2B were significantly associated with meningitis in our cohort, while virulence genes *cylE, lmb, fbsA, fbsC, iagA* and *pbsP* showed no statistically significant difference in occurrence between sepsis and meningitis cases.

**Implications of all the available evidence**

Although virulence profiles evolved over time, potential vaccine coverage remained high, with an estimated 97% of cases preventable by the hexavalent GBS6 vaccine and 99% by the GBS-AlpN candidate. These findings strongly underscore the urgent need to consider maternal GBS vaccination as a more effective and durable prevention strategy.

## Introduction

*Streptococcus agalactiae* (Group B Streptococcus, GBS) is the leading cause of sepsis and meningitis in young infants.^1^ Invasive GBS disease manifests as early-onset disease (EOD), occurring within the first week of life, typically from vertical transmission, or late-onset disease (LOD), presenting between 7 and 89 days of life, with more diverse transmission routes.^1^^,^^2^

GBS is an encapsuled Gram-positive bacterium classified into serotypes (Ia–IX) based on its capsular polysaccharide.^1^ Multi-locus sequence typing (MLST) further distinguishes isolates into sequence types (STs), and clonal complexes (CCs). Five major CC groups, CC1, CC12, CC17, CC19, and CC23, are associated with colonisation or invasive disease.^3^ Molecular and functional studies have identified multiple virulence factors in GBS, some associated with hypervirulent clones and others with invasive disease, including meningitis. Factors associated with meningitis include hypervirulent GBS adhesin (HvgA), serine-rich repeat (Srr) proteins, pilus island (PI) proteins, fibrinogen-binding (Fbs) proteins, invasion-associated gene A (IagA), β-hemolysin/cytolysin exporter E (CylE), laminin-binding (Lmb) protein and plasminogen-binding surface protein (PsbP).^4^ Whole-genome sequencing (WGS) enables the systematic detection of genes encoding these virulence factors in clinical isolates.^4^^,^^5^

Despite risk factor-based intrapartum antibiotic prophylaxis (IAP) since 1998, the incidence of GBS disease in the Netherlands increased from 0·19 per 1000 live births in 1987 to 0·36 in 2015, largely driven by CC17 and CC23.^6^, ^7^, ^8^ Similar trends have been reported in the United Kingdom, which also uses a risk factor-based IAP strategy.^9^ In contrast, screening-based IAP has reduced EOD incidence in France, the United States and Canada.^10^, ^11^, ^12^ Although GBS remains universally susceptible to penicillin, increasing resistance to alternative antibiotics highlights the need for ongoing surveillance. Consequently, GBS has been prioritised by the World Health Organisation for vaccine development.^13^^,^^14^ Two vaccine types are currently in phase 2 or phase 3 clinical evaluation: a hexavalent glycoconjugate vaccine (GBS6), targeting serotypes Ia–V, and a protein-subunit vaccine (GBS-AlpN).^4^^,^^15^ GBS-AlpN targets four peptides that correspond to the N-terminal domain of five members of the surface-anchored Alpha-like protein (Alp) family (Alp 1, Alp 2/3, Alpha C and Rib).^15^^,^^16^

We assessed the molecular epidemiology of GBS disease in young infants in the Netherlands over 37 years of bacteriological surveillance, evaluated the impact of the current prevention programme on disease incidence and estimated the potential coverage of candidate maternal vaccines and the distribution of genes encoding GBS virulence factors.

## Methods

### Study design and participants

We identified young infants age 0–89 days with GBS culture-positive sepsis or meningitis in the Netherlands between July 1st, 1987 and June 30th, 2024. Cases were identified through the Netherlands Reference Laboratory for Bacterial Meningitis (NRLBM). Submission of isolates is on a voluntary basis and criteria for isolate submission have changed only slightly over the study time frame. Between 1987 and July 2016, the submission criteria were to submit all bacterial strains cultured from cerebrospinal fluid (CSF) and/or blood from neonates ≤4 weeks of age in case of bacterial meningitis and/or sepsis; infants aged 29 days to < 1 year were not systematically included during this period. In July 2016, the criteria were expanded to include all infants <1 year of age. The NRLBM receives approximately 85–90% of CSF and blood isolates from all patients with bacterial meningitis in the Netherlands; comparable coverage for blood culture isolates from infants with suspected sepsis has not been studied but is expected to be similar, as all microbiology laboratories participate in the same surveillance network.^17^ From 2018 onwards, treating physicians could also contact the study team to include patients who had not yet been reported by the NRLBM; this contributed a small number of additional cases. Clinical data were not systematically collected as part of this laboratory-based surveillance system.

### Definitions

Sepsis was defined as a GBS-positive blood culture and meningitis was defined as a GBS-positive CSF culture with or without a concomitant positive blood culture. Age of onset was calculated as the number of days between the date of birth and the start of symptoms, or if unavailable, the earliest date that positive cultures were obtained. If both the date of first symptoms and culture dates were unavailable, the date the isolate was received by the NRLBM was used to calculate the age of onset. EOD was defined as start of symptoms before 7 days of life (0–6 days) and LOD was defined as start of symptoms after 6 days of life (7–89 days).

### Procedures

Serotype was determined by latex agglutination, as described previously.^18^ A targeted literature search was performed to select virulence factors associated with neonatal GBS meningitis to investigate their presence with WGS (Supplementary File S1). Seven reviews on GBS pathogenesis were screened. Virulence factors that were explicitly discussed to be associated with neonatal meningitis in at least two reviews were selected (HvgA, Srr, PI, CylE, Lmb, FbsA, FbsB, FbsC, IagA and PbsP, Supplementary File S1).

Genomic DNA extraction was performed as described previously.^6^ The extracted DNA was submitted to the Wellcome Sanger Institute for WGS. Tagged DNA libraries were created using NEBNext® UltraTM II DNA Library Prep Kit for Illumina and WGS was performed on the Illumina NovaSeq 6000 platform with 150 bp paired-end reads. The sequence data was assessed using an established GBS pipeline^19^ and sequences that passed quality control were analysed using the GBS Typer pipeline^20^ to determine ST, genotype-based serotypes and the presence of virulence factor genes *alp*, *hvgA, srr* and PI alleles. CCs were assigned based on STs, which are defined by the allelic profiles of housekeeping genes in PubMLST.^21^ Presence of additional virulence genes was determined with SRST2^22^ using a custom virulence gene database consisting of reference sequences extracted from the virulence factor database (VFDB^23^: *cylE, lmb, fbsA* and *fbsB*) and additional genes of interest extracted from selected reference genomes (*iagA, pbsP* and *fbsC*, Supplementary File S2). Gene presence was determined using the default SRST2^22^ thresholds for gene reporting (e.g., minimum 90% coverage). Any genes mapping below these thresholds were classified as absent. Sequence data generated in this study are available in the European Nucleotide Archive (ENA) under three project accessions: PRJEB14124, PRJEB47396, and PRJEB91039. Accession numbers for individual isolates analysed in this work are provided in Supplementary File S3.

### Statistical analysis

Statistical analyses were performed using R (RStudio, version 4.3.2). Incidences were calculated with year-matched population data obtained from Statistics Netherlands with the use of StatLine.^24^ Annual incidence was calculated as the number of new episodes per epidemiological year (1 July–30 June) per 1000 live births; thus, any reference to a given year (e.g., 1987) corresponds to the epidemiological year spanning July 1987 to June 1988. Clinical characteristics were described using frequencies and percentages or the median and interquartile range (IQR). Differences were tested with the Mann–Whitney *U* test for continuous variables. The Fisher's Exact test was used to compare categorical data. Univariable linear regression was used to study the association between year of the observation period and incidence, based on aggregated data per year. Analyses were performed for overall incidence (including both sepsis and meningitis) as well as separately for sepsis and meningitis incidence. The assumption of linearity was checked graphically. Interrupted time series analyses were used to study the effect of the implementation of IAP in the Netherlands. Univariable logistic regression was used to study the associations between sex, age of onset, serotypes, virulence factors, and the outcome, defined as diagnosis (meningitis versus sepsis). Logistic regression analyses were performed using individual-level data, with each row representing one infant. Missing values were expected to be random and uncommon; no imputation was performed. The statistical tests were all two-tailed and a p value of less than 0·05 was considered as statistically significant.

### Role of the funding source

The funding source had no role in the design of the study, data collection, data analysis, data interpretation, writing of the manuscript, or the decision to submit for publication.

## Results

### Epidemiology

**Fig. 1:**
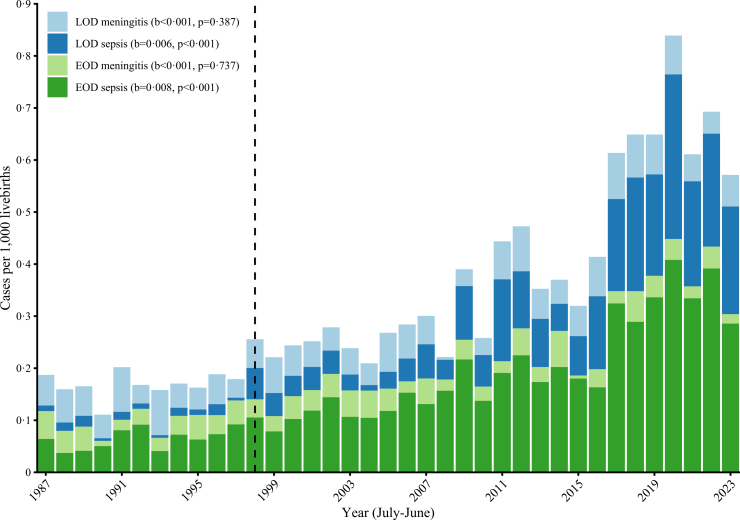
**Incidence of group B streptococcal meningitis and sepsis in infants (0***–***89 days old) per 1000 live births.** The dashed vertical line represent the implementation of risk-based intrapartum antibiotic prophylaxis in 1998 in the Netherlands. Disease onset was calculated based on age of onset, which was calculated by the first culture date or the date the isolate was received by the NRLBM. EOD = early onset disease (0*–*6 days), LOD = late onset disease (7*–*89 days), b = beta-coefficient, NRLBM = Netherlands Reference Laboratory for Bacterial Meningitis.

In the study period July 1st, 1987 until June 30th, 2024, 2212 episodes with a GBS-positive blood or CSF culture in 2180 infants age 0–89 days old were identified through the NRLBM, of which a small number of cases (n = 21) were identified through treating physicians between 2018 and 2024. The mean annual incidence of invasive GBS disease was 0·33 (95% CI 0·27–0·39) per 1000 live births, with a significant increase over time from 0·19 per 1000 live births in 1987 to 0·57 in 2023, with an incidence peak in 2020 of 0·84 per 1000 live births (b = 0·015, p < 0·0001, Fig. 1, Supplementary Table S1). Mean annual incidence of GBS sepsis was 0·23 (95% CI 0·18–0·30) per 1000 live births and significantly increased over time (b = 0·015, p < 0·0001). Mean annual incidence of GBS meningitis was 0·09 (95% CI 0·09–0·10) per 1000 live births and remained stable over time (b < 0·001, p = 0·62, Supplementary Table S1).

For 2153 (97%) episodes the patient's sex was known, of whom 996 (46%) occurred in females (Table 1). EOD accounted for 1307 (59%) and LOD for 905 (41%) of 2212 episodes. Sepsis occurred in 1569 (71%) and meningitis occurred in 643 (29%) of 2212 episodes, of which 440 (68%) also had a positive blood culture (both sepsis and meningitis) and 203 (32%) had meningitis alone. Meningitis was significantly more common in LOD compared to EOD (61% vs 39%, OR 3·2 [95% CI 2·7–3·9], p < 0·0001, Table 1). 32 (1·5%) of 2180 patients experienced a GBS recurrent episode, with a median time interval of 20 days (IQR 14–30 days).

**Table 1:** Group B streptococcal disease in young infants in the Netherlands, 1987–2023.

	Overall, N = 2212	Meningitis, N = 643	Sepsis, N = 1569	p value^a^
Sex				0·18
Female	996/2153 (46%)	300/618 (49%)	696/1535 (45%)	
Male	1157/2153 (54%)	318/618 (51%)	839/1535 (55%)	
Age of onset				<0·0001
EOD (0–6 days)	1307/2212 (59%)	251/643 (39%)	1056/1569 (67%)	
LOD (7–89 days)	905/2212 (41%)	392/643 (61%)	513/1569 (33%)	
Serotype				
Ia	383/2163 (18%)	73/625 (12%)	310/1538 (20%)	<0·0001
Ib	107/2163 (5%)	20/625 (3%)	87/1538 (6%)	0·02
II	123/2163 (6%)	6/625 (1%)	117/1538 (8%)	<0·0001
III	1316/2163 (61%)	504/625 (81%)	812/1538 (53%)	<0·0001
IV	50/2163 (2%)	1/625 (<1%)	49/1538 (3%)	0·003
V	116/2163 (5%)	10/625 (2%)	106/1538 (7%)	<0·0001
VI	13/2163 (1%)	1/625 (<1%)	12/1538 (1%)	0·13
VII	3/2163 (<1%)	0/625 (0%)	3/1538 (<1%)	0·99
VIII	1/2163 (<1%)	0/625 (0%)	1/1538 (<1%)	0·97
IX	11/2163 (1%)	1/625 (<1%)	10/1538 (1%)	0·18
Non-typeable	40/2163 (2%)	9/625 (1%)	31/1538 (2%)	0·36
Alp protein				
*alp1*	291/1723 (17%)	59/549 (11%)	232/1174 (20%)	<0·0001
*alp2/3*	72/1723 (4%)	9/549 (2%)	63/1174 (5%)	<0·001
*alphaC*	243/1723 (14%)	45/549 (8%)	198/1174 (17%)	<0·0001
*rib*	1103/1723 (64%)	431/549 (79%)	672/1174 (57%)	<0·0001
None	14/1723 (1%)	5/549 (1%)	9/1174 (1%)	0·77

Data are shown as n/N (%).

Abbreviations: EOD = early onset disease, LOD = late onset disease, Alp = Alpha-like protein.

a Meningitis versus sepsis with Fisher's Exact test.

Mean annual incidence for EOD was 0·20 (95% CI 0·16–0·23) and 0·14 (95% CI 0·11–0·17) for LOD per 1000 live births. Both significantly increased during the observation period from 0·12 and 0·07 in 1987 to 0·30 and 0·27 in 2023, respectively (p < 0·0001), with peak incidences in 2020 of 0·45 and 0·39, respectively (Supplementary Table S1). The relative proportions of EOD and LOD remained unchanged after implementation of IAP in 1998 (p = 0·57), and interrupted time series analysis showed no immediate or trend-level effect of IAP on EOD incidence (level change: β = −0·025, p = 0·43; change in trend: β = 0·0076 per year, p = 0·10).

The majority of cases (>80%) occurred in infants aged 28 days or younger, with a median of 88% across all years. Age at disease onset distribution is shown in Supplementary Figure S1. The mean annual incidence of GBS disease in patients aged 28 days or younger from 1987 to 2015 was 0·22 (95% CI 0·19–0·25) per 1000 live births and increased to 0·53 (95% CI 0·45–0·61) per 1000 live births in 2016–2023, while the incidence of patients aged 28 or older increased from 0·03 (95% CI 0·02–0·03) to 0·10 (95% CI 0·07–0·13) per 1000 live births.

### Molecular and genomic characteristics

**Fig. 2:**
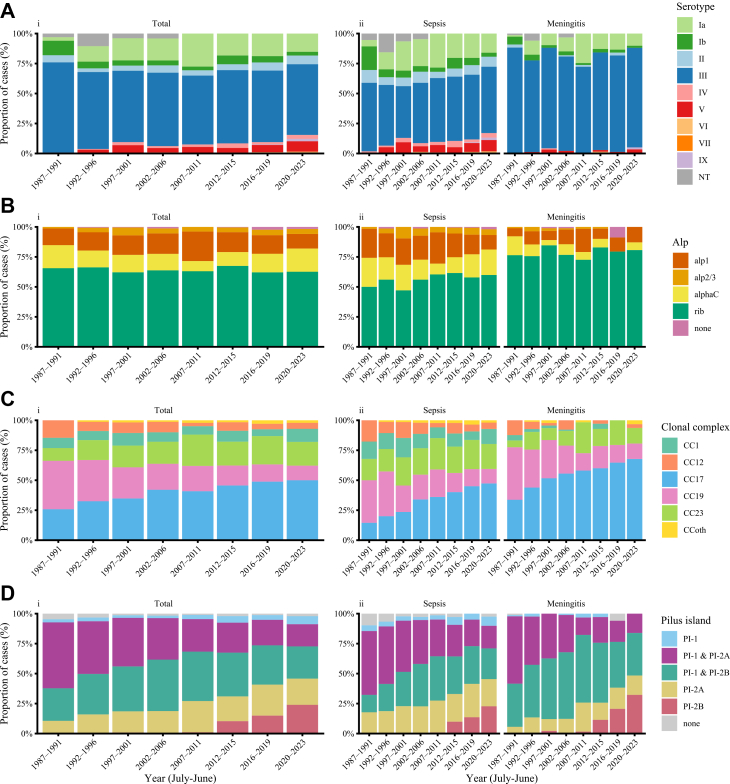
**Serotype, clonal complex and virulence factor distribution of group B streptococcal isolates per year of infection and source of infection.** (A) Serotype distribution. (B) Alp protein distribution. (C) Clonal complex distribution and (D) Pilus island distribution of (i) all cases and (ii) split by diagnosis (sepsis or meningitis; based on source of isolate) and grouped by indicated epidemiological years. Alp = Alpha like protein, CC = clonal complex, CCoth = CC other, PI = pilus island.

Serotype data was available for 2163 (98%) of 2212 isolates (Table 1). Serotype III was most common (61%), followed by Ia (18%), II (6%), V (5%) and Ib (5%). In total, 90% of all sepsis cases were caused by 5 different serotypes (Ia, Ib, II, III, and V), while 90% of meningitis cases were caused by serotypes Ia and III (Fig. 2A and Supplementary Table S2). Serotype III caused 81% of meningitis cases compared to 53% of sepsis cases (OR 3·7 [95% CI 3·0–4·7], p < 0·0001, Table 1). Serotype IV was strongly associated with sepsis (49 out of 50 cases; OR 20·7 [95% CI 2·8–151·5], p = 0·003, Table 1) and caused only one meningitis case. Serotype distribution shifted over time, with an increase in serotype Ia (9% [25/295] in 1987–1996 vs 17% [160/954] in 2014–2023, p < 0·0001) and decreases in serotype Ib and III (9% and 69% in 1987–1996 vs 4% and 60% in 2014–2023 respectively, p < 0·01, Fig. 2A and Supplementary Table S2). Serotypes VI, VII and IX were only detected after 2002. Among the 1770 isolates with known genotype-based serotypes, 1651 (93%) matched the phenotypic latex serotype and 95 (5%) showed a discrepancy, of which 39 (41%) were non-typeable by latex but had a defined genotype-based serotype.

In total, 1773 (80%) of the 2212 isolates were sequenced, of which 1723 (97%) passed quality control. From the Alp-encoding genes, *rib* was most commonly present (64%), followed by a*lp1* (17%) and *alphaC* (14%) (Table 1). Fourteen (1%) isolates had no Alp-encoding gene. *rib* was associated with meningitis (OR 2·7 [95% CI 2·2–3·4], p < 0·0001; Table 1) and present in 99% of CC17 isolates (Supplementary Table S3). In contrast, Alp 1, Alp 2/3 and Alpha C-encoding genes were associated with sepsis (OR 2·1 [95% CI 1·6–3·0], OR 3·5 [95% CI 1·6–7·3], OR 2·2 [95% CI 1·5–3·2], respectively; all p < 0·001; Table 1). Distribution of the Alp-encoding genes remained stable over time (Fig. 2B and Supplementary Table S2).

Isolates were assigned to 144 unique STs and classified into 5 major CC groups (CC1, CC12, CC17, CC19 and CC23), representing 98% of isolates (Supplementary Table S3). The remaining isolates (2%, n = 29) were classified as CC other (5 different CCs). CC17 was most common (41%), followed by CC19 (22%) and CC23 (20%). Over time, CC17 increased from 29% (90/308) in 1987–1996 to 49% (257/526) in 2014–2023 (p < 0·0001), while CC19 declined from 37% (115/308) to 15% (78/526, p < 0·0001, Fig. 2C and Supplementary Table S2). CC17 and CC19 were associated with meningitis (OR 1·9 [95% CI 1·6–2·4] and OR 1·4 [95% CI 1·1–1·8], respectively), whereas CC1, CC12, and CC23 were associated with sepsis (all p < 0·05; Supplementary Table S3).

Of the pilus islands, the combination of PI-1 and PI-2A (32%) or PI-1 and PI-2B (36%) per isolate was most common, followed by PI-2A or PI-2B alone (20% and 7%, respectively; Supplementary Table S3). The combination of PI-1 and PI-2B was associated with meningitis (OR 2·0 [95% CI 1·7–2·5], p < 0·0001), whereas PI-1 and PI-2A were significantly more common in sepsis (Supplementary Tables S2 and S3). *HvgA* was present in 708 (41%) of 1723 isolates; 52% of meningitis isolates and 36% of sepsis isolates were *HvgA*-positive (OR 1·9 [95% CI 1·5–2·3], p < 0·0001). The presence of *srr1* was associated with sepsis (OR 1·8 [95% CI 1·5–2·3], p < 0·0001) and the presence of *srr2* was associated with meningitis (OR 1·9 [95% CI 1·5–2·3], p < 0·0001). *FbsA* was present in 74% of isolates; 71% of meningitis isolates and 75% of sepsis isolates were *fbsA*-positive (p = 0·08). The presence of *fbsB* was associated with meningitis (OR 3·6 [95% CI 1·3–15·4], p = 0·04). All other investigated GBS meningitis-associated virulence genes (*cylE*, *lmb*, *fbsC*, *iagA* and *psbP*) were detected in almost all isolates, with no statistically significant difference between sepsis and meningitis isolates (Supplementary Table S3).

**Fig. 3:**
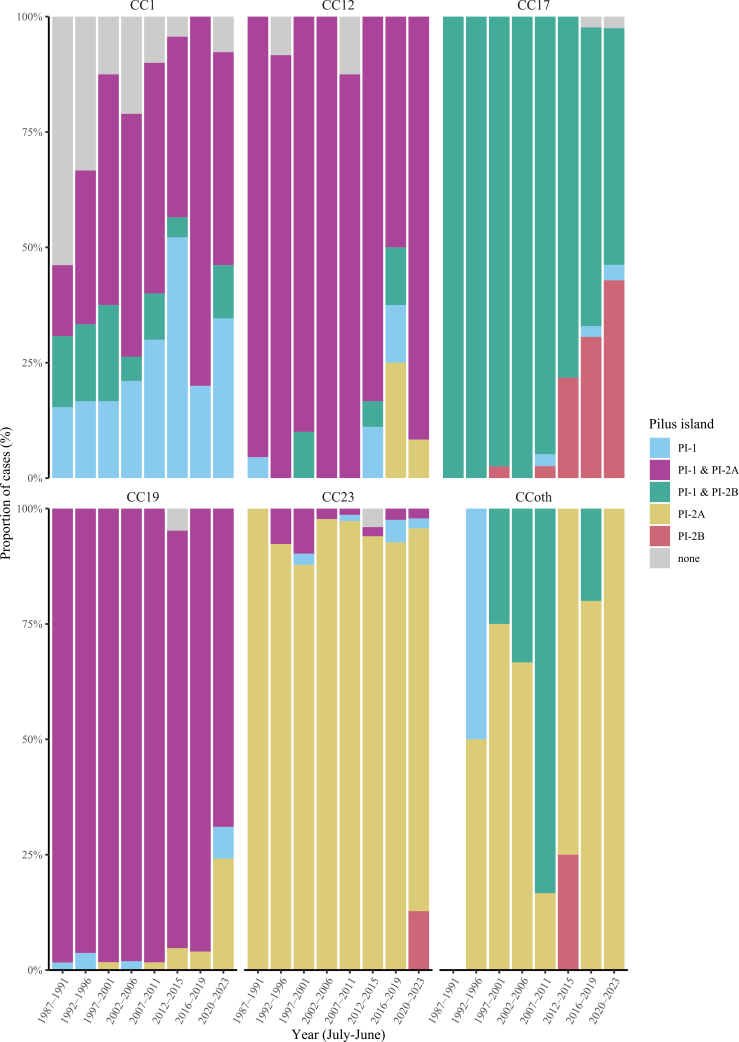
**Pilus island distribution across most common clonal complex groups and epidemiologic year of infection, in groups.** CC = clonal complex, CCoth = CC other, PI = pilus island.

The distribution of some virulence factors was strongly associated with specific CCs, with different patterns observed in each CC (Supplementary Table S4) and changes during the study period (Fig. 3 and Supplementary Table S5). *HvgA* and s*rr2* were almost only present in CC17 isolates, while *srr1* was present in the majority of all other CC groups. *FbsA* was present in most of CC groups, except CC19, and *fbsB* was present in all groups, except the CC other group (Supplementary Table S4). Furthermore, 100% (n = 39) of CC17 isolates from the period 1987–1991 showed presence of PI-1 and PI-2B, whereas from 2012 onwards, the proportion of CC17 isolates with presence of only PI-2B progressively increased towards 43% (51/119) in 2020–2023 (Fig. 3 and Supplementary Table S5). In contrast, CC19 isolates initially (1987–1991) showed presence of both PI-1 and PI-2A (98%, 60/61) and over time, this combination declined markedly with replacement of PI-2A alone towards 24% (7/29) in 2020–2023 (Fig. 3 and Supplementary Table S5). All other investigated virulence genes (*cylE*, *lmb*, *fbsC*, *iagA* and *psbP*) were equally distributed across all CC groups (Supplementary Table S4).

### Vaccine coverage

**Fig. 4:**
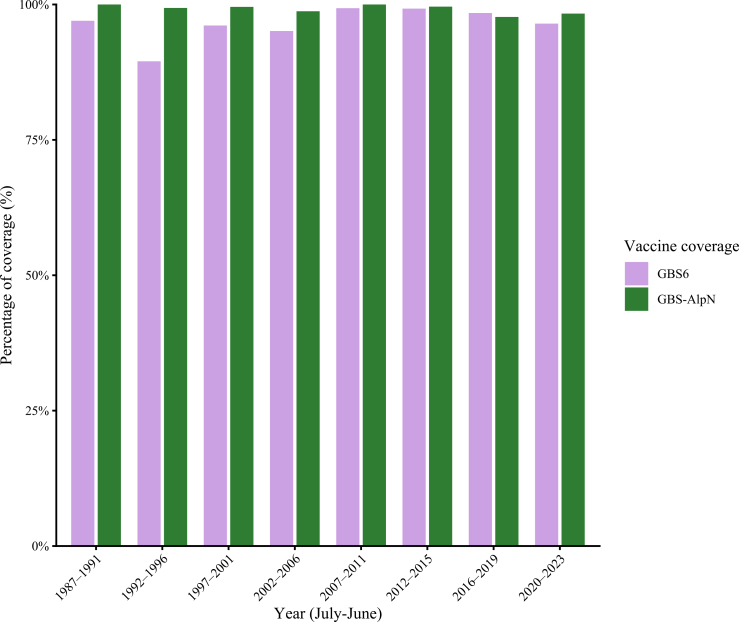
**Vaccine coverage of group B streptococcal cases based on isolate characteristics, in percentages, during the study period, epidemiologic years 1987**–**2023.** Vaccines GBS6 and GBS-AlpN were studied.

We assessed potential strain coverage for the current phase 2 and 3 vaccine candidates, GBS6, and GBS-AlpN. For the GBS6 vaccine, 2095 (97%) of 2163 cases of which serotype was known, representing 96% of sepsis and 98% of meningitis cases, would potentially be covered. Coverage remained high throughout the study period, ranging from 90% to 99%, with no clear change over time (Fig. 4). Reflecting on the last 4 investigated years (2020–2023), 437 (96%) of 453 cases would have been covered by the GBS6 vaccine. The GBS-AlpN vaccine demonstrated the highest potential coverage, covering 99% (1709/1723) of cases over the entire study period, which is statistically higher than the GBS6 coverage (p = 0·001). The GBS-AlpN coverage consistently exceeding 98% across all evaluated time intervals and clinical presentations (range 98%–100%, Fig. 4).

## Discussion

In this nationwide study spanning 37 years of epidemiological surveillance, we found that the incidence of invasive GBS disease in young infants in the Netherlands has risen steadily, driven predominantly by an increase in GBS sepsis, while the incidence of GBS meningitis remained stable. Overall, incidence levels were comparable with other high-income countries, including the United Kingdom and the United States.^9^^,^^11^^,^^12^ The persistent increase in GBS sepsis over nearly four decades suggests a genuine rise in disease burden, although the underlying drivers remain incompletely understood. Improvements in blood culture diagnostics, together with variation in the use of lumbar puncture during neonatal sepsis evaluations, particularly in very low birth weight infants and in light of early-onset sepsis guideline recommendations where lumbar puncture is not routinely advised, may have contributed to higher detection of bloodstream infections and a shift in case classification from meningitis to sepsis.^7^^,^^25^ We observed a minor increase in incidence in older infants (>28 days), this is explained by a change in submission criteria for isolate submission during the study period, where infants aged >28 days were not systematically included prior to 2016. These cases are therefore likely underrepresented in earlier years. However, the incidence among infants 28 days or younger more than doubled, while criteria for this age group did not change during these years, indicating that the upward trend cannot be explained solely by broader inclusion criteria. These findings indicate that the continuing rise in GBS sepsis likely reflects a combination of improved detection and increasing transmission and disease, underscoring the limitations of current prevention strategies.

The rising incidence of invasive disease in the Netherlands appears closely linked to the expanding dominance of the hypervirulent CC17 that has a well-established association with severe neonatal disease.^4^ We observed a substantial increase in CC17 isolates over time. CC17 carries a distinct set of virulence factors, most notably HvgA, Srr2 and the combination of PI-1 and PI-2B, or PI-2B alone. HvgA promotes intestinal colonisation and translocation across both intestinal and blood–brain barriers, facilitating meningitis development. Srr2, further facilities neuroinvasion and PI-2B has become increasingly common among recent isolates, supporting the competitive advantage of this pilus island.^4^^,^^5^^,^^26^ In line with the predominance of CC17, serotype III was the most prevalent serotype in our cohort. However, the relative prevalence of CC17 and serotype III may vary geographically and over time, highlighting the importance of continued surveillance to monitor temporal and regional changes in strain distribution.^1^

We examined a panel of virulence genes previously implicated in GBS meningitis (*hvgA, srr2,* PI*, cylE, lmb, fbsA, fbsB, fbsC, iagA,* and *pbsP,* Supplementary File S1). As expected, HvgA and Srr2 were almost only present in CC17 isolates and associated with meningitis. The combination of PI-1 and PI-2B was also associated with meningitis, and the proportion of CC17 isolates carrying PI-2B alone increased progressively over the study period. FbsB was present in 98% of all isolates, with a significantly higher prevalence in meningitis cases. All other investigated virulence genes previously linked to GBS meningitis (*cylE, lmb, fbsC, iagA,* and *pbsP*) were present in nearly all isolates, with no differences between sepsis and meningitis cases and no variation across CC groups. The only exception was FbsA, which was largely absent in CC19 isolates. Although prior mouse model studies identified these virulence factors as meningitis determinants,^5^^,^^27^^,^^28^ our findings confirm an association only for HvgA, Srr2, FbsB and PI. The remaining factors (*cylE, lmb, fbsA, fbsC, iagA,* and *pbsP*) were equally distributed among sepsis and meningitis cases, suggesting that these genes belong to the conserved GBS gene repertoire rather than being disease-specific. This discrepancy between murine models and human disease may reflect differences in gene expression or functional activity, as genomic presence alone does not capture virulence factor regulation during invasive infection. More research is needed to determine whether these virulence factors are differentially expressed during invasive disease.

Our data indicate that the current Dutch risk-based IAP strategy does not adequately prevent early-onset GBS disease. In countries with screening-based IAP programs, such as the United States and Canada, EOD incidence has declined.^10^, ^11^, ^12^ In addition, the proportion of EOD cases presenting as meningitis in our cohort (19%) is comparable to estimates from global meta-analyses (∼16%), but somewhat higher than reported in countries with screening-based IAP programs, where meningitis accounts for approximately 10% of EOD cases.^10^ Despite guideline implementation, we observe neither a decline in overall incidence nor a relative reduction of EOD in the Netherlands, indicating that the introduction of risk-based IAP was not associated with an immediate or trend-level effect. This is consistent with our previous findings, showing that the Dutch guidelines, which are based on the United Kingdom NICE guidelines, have poor sensitivity for identifying neonates at risk.^29^ The Dutch risk-based strategy has remained largely unchanged over the study period: there were no national modifications in microbiological reporting or interpretation of maternal GBS bacteriuria, and although intrapartum PCR screening has become available it has not been systematically implemented nationally. Similar patterns have been observed internationally: in the United Kingdom, where risk-based IAP is also used, EOD incidence increased from 0·48 per 1000 live births in 2000–2001 to 0·57 per 1000 in 2014–2015, reinforcing that risk-based strategies alone may be insufficient to prevent EOD.^9^

In contrast, our vaccine coverage analysis is strongly favourable: candidate GBS vaccines would potentially have provided protection against more than 96% of invasive cases throughout the entire 37-year period. This high and stable coverage supports maternal vaccination as a promising preventive strategy. This is particularly relevant in comparison with antibiotic prophylaxis, which targets prevention of EOD and is ineffective against LOD. We have previously shown that maternal GBS immunisation could be cost-effective when implemented alongside current risk factor-based approach in the Netherlands.^30^

This study has limitations. First, case numbers are likely underestimated because not all invasive GBS infections are culture-positive and some cases may therefore remain undiagnosed. In particular, lumbar punctures are not always performed or may be delayed until after antibiotic treatment, as reported in the literature, which can further contribute to underestimation.^25^ Second, not all culture-confirmed cases are included in our national bacteriological surveillance. However, previous studies have estimated that for other invasive bacterial infections (e.g., invasive meningococcal and pneumococcal disease) approximately 85–90% of isolates were submitted to the NRLBM.^17^ Third, WGS data were available for approximately 80% of isolates in our study, with underrepresentation in the most recent years. Finally, due to the nature of this laboratory-based surveillance dataset, detailed clinical information was limited, which precluded adjustment for potential confounders and restricted regression analyses to univariable models, and restricted sepsis and meningitis definition to culture results instead of a clinical diagnosis. Nonetheless, the large sample size, long study duration, and the combined use of phenotypic serotyping and genomic data provide a reliable overview of GBS disease epidemiology and bacterial population characteristics in the Netherlands.

In conclusion, this nationwide analysis shows a sustained and substantial increase in invasive GBS disease in young infants in the Netherlands, with no discernible effect of the current risk-based IAP strategy. The current epidemiology is dominated by CC17 and serotype III, both strongly associated with meningitis. Although virulence profiles evolved over time, vaccine coverage was high during all years with over 96% of cases potentially covered by the hexavalent GBS6 vaccine, and 99% by the broader GBS-AlpN candidate. These findings strongly underscore the urgent need to consider maternal GBS vaccination as a more effective and durable prevention strategy.

## Contributors

MAG: data curation, formal analysis, investigation, methodology, visualisation, writing–original draft. DJ: investigation, methodology, writing–review & editing. DHV: writing–review & editing. MCB: conceptualisation, funding acquisition, methodology, writing–review & editing. DvdB: conceptualisation, funding acquisition, methodology, writing–review & editing. NMvS: conceptualisation, methodology, resources, writing–review & editing. MWB: conceptualisation, investigation, methodology, project administration, resources, supervision, writing–review & editing.

## Data sharing statement

Sequence data generated in this study are available in the European Nucleotide Archive (ENA) under three project accessions: PRJEB14124, PRJEB47396, and PRJEB91039. Accession numbers for individual isolates analysed in this work are provided in Supplementary File S3. De-identified individual participant data underlying the findings of this study are available from the corresponding author upon reasonable request.

## Declaration of interests

DvdB reports grants from the Netherlands Organisation for Scientific Research (NWO Vici grant 918.19.627). MCB reports grants from the European Research Council (ERC Consolidator grant 101001237) and grants from Amsterdam UMC, and the ItsME foundation outside the submitted work. MWB reports grants from Amsterdam UMC and the ItsME foundation outside the submitted work. All other authors declare no competing interests.

## Appendix A Supplementary data

Supplementary Tables and Figures

Collab Authors
