# Supplementary material for: Group B streptococcal disease in infants 0–3 months in The Netherlands, 1987–2023: a nationwide genomic and epidemiological surveillance study
Source: Lancet Reg Health Eur. 2026 May 12;66:101711. doi: 10.1016/j.lanepe.2026.101711 (PMC13193784; doi:10.1016/j.lanepe.2026.101711)
Supplement: Supplementary Tables and Figures [file mmc1.docx]

**GROUP B STREPTOCOCCAL DISEASE IN INFANTS 0-3 MONTHS IN THE NETHERLANDS, 1987-2023: A NATIONWIDE GENOMIC AND EPIDEMIOLOGICAL SURVEILLANCE STUDY**

Marleen A. Groenveld^1^, Dorota Jamrozy^2^, Douwe H. Visser^3^, Matthijs C. Brouwer^1^, Diederik van de Beek^1^, Merijn W. Bijlsma^1,4*^, Nina M. van Sorge^5*^, on behalf of the NOGBS study group

1. Department of Neurology, Amsterdam University Medical Centre, University of Amsterdam, Amsterdam, The Netherlands.
2. Parasites and Microbes Programme, Wellcome Sanger Institute, Wellcome Genome Campus, Hinxton, Cambridge, United Kingdom.
3. Department of Neonatology, Emma Children’s Hospital, Amsterdam University Medical Centre, University of Amsterdam, Amsterdam, The Netherlands.
4. Department of Paediatrics, Emma Children’s Hospital, Amsterdam University Medical Centre, University of Amsterdam, Amsterdam, The Netherlands.
5. Department of Medical Microbiology and Infection Prevention, Netherlands Reference Laboratory for Bacterial Meningitis, Amsterdam University Medical Centre, University of Amsterdam, Amsterdam, The Netherlands.

* authors contributed equally

**Supplementary material**

**Contents**

Supplementary Table 1 *-* Incidence Group B Streptococcal disease in the Netherlands, per

epidemiologic years 1987-2023 page 3

Supplementary Figure 1 - Distribution of Group B Streptococcal disease by age page 5

Supplementary Table 2 - Serotype, clonal complex and virulence factor distribution

of Group B Streptococcal isolates per year of infection

and source of infection page 6

Supplementary Table 3 - Virulence factor distribution between sepsis and meningitis isolates page 8

Supplementary Table 4 - Serotype and virulence factor distribution among the clonal complex

groups page 10

Supplementary Table 5 - Pilus island distribution across most common clonal complex groups

and epidemiologic year of infection, in groups page 12

Supplementary File 1 - Targeted literature search page 13

Supplementary File 2 - Custom virulence database page 15

Supplementary File 3 - Sequence data accession numbers page 23

**Supplementary Table 1 *-* Incidence Group B Streptococcal disease in the Netherlands, per epidemiologic years 1987-2023**

| **Year of infection**^1^ | **Total number**  **of cases** | **Number of live births (x1,000)^2^** | **Total annual incidence** | **EOD incidence** | **LOD incidence** | **Meningitis incidence** | **Sepsis incidence** | **Serotype III incidence** |
| --- | --- | --- | --- | --- | --- | --- | --- | --- |
| **1987** | 35 | 187·0 | 0·19 | 0·12 | 0·07 | 0·11 | 0·07 | 0.09 |
| **1988** | 30 | 188·0 | 0·16 | 0·08 | 0·08 | 0·11 | 0·05 | 0·11 |
| **1989** | 32 | 193·5 | 0·17 | 0·09 | 0·08 | 0·10 | 0·06 | 0·12 |
| **1990** | 22 | 198·5 | 0·11 | 0·06 | 0·05 | 0·06 | 0·06 | 0·08 |
| **1991** | 40 | 198·0 | 0·20 | 0·10 | 0·10 | 0·11 | 0·10 | 0·12 |
| **1992** | 33 | 196·5 | 0·17 | 0·12 | 0·05 | 0·07 | 0·10 | 0·13 |
| **1993** | 31 | 196·0 | 0·16 | 0·07 | 0·09 | 0·11 | 0·05 | 0·12 |
| **1994** | 33 | 193·5 | 0·17 | 0·11 | 0·06 | 0·08 | 0·09 | 0·10 |
| **1995** | 31 | 190·5 | 0·16 | 0·11 | 0·05 | 0·09 | 0·07 | 0·08 |
| **1996** | 36 | 191·0 | 0·19 | 0·11 | 0·08 | 0·09 | 0·09 | 0·10 |
| **1997** | 35 | 195·5 | 0·18 | 0·14 | 0·04 | 0·08 | 0·10 | 0·11 |
| **1998** | 51 | 199·5 | 0·26 | 0·14 | 0·12 | 0·09 | 0·17 | 0·14 |
| **1999** | 45 | 203·5 | 0·22 | 0·11 | 0·11 | 0·10 | 0·12 | 0·15 |
| **2000** | 50 | 205·0 | 0·24 | 0·15 | 0·10 | 0·10 | 0·14 | 0·15 |
| **2001** | 51 | 202·5 | 0·25 | 0·16 | 0·09 | 0·09 | 0·16 | 0·13 |
| **2002** | 56 | 201·0 | 0·28 | 0·19 | 0·09 | 0·09 | 0·19 | 0·16 |
| **2003** | 47 | 197·0 | 0·24 | 0·16 | 0·08 | 0·10 | 0·14 | 0·16 |
| **2004** | 40 | 191·0 | 0·21 | 0·16 | 0·05 | 0·09 | 0·12 | 0·12 |
| **2005** | 50 | 186·5 | 0·27 | 0·16 | 0·11 | 0·12 | 0·15 | 0·18 |
| **2006** | 52 | 183·0 | 0·28 | 0·17 | 0·11 | 0·09 | 0·20 | 0·17 |
| **2007** | 55 | 183·0 | 0·30 | 0·18 | 0·12 | 0·10 | 0·20 | 0·22 |
| **2008** | 41 | 185·0 | 0·22 | 0·18 | 0·04 | 0·03 | 0·19 | 0·13 |
| **2009** | 72 | 184·5 | 0·39 | 0·25 | 0·14 | 0·07 | 0·32 | 0·24 |
| **2010** | 47 | 182·0 | 0·26 | 0·16 | 0·09 | 0·06 | 0·20 | 0·13 |
| **2011** | 79 | 178·0 | 0·44 | 0·21 | 0·23 | 0·10 | 0·35 | 0·20 |
| **2012** | 82 | 173·5 | 0·47 | 0·28 | 0·20 | 0·14 | 0·33 | 0·26 |
| **2013** | 61 | 173·0 | 0·35 | 0·20 | 0·15 | 0·09 | 0·27 | 0·23 |
| **2014** | 64 | 173·0 | 0·37 | 0·27 | 0·10 | 0·12 | 0·25 | 0·22 |
| **2015** | 55 | 172·0 | 0·32 | 0·19 | 0·13 | 0·06 | 0·26 | 0·22 |
| **2016** | 71 | 171·5 | 0·41 | 0·20 | 0·22 | 0·11 | 0·30 | 0·29 |
| **2017** | 104 | 169·5 | 0·61 | 0·35 | 0·27 | 0·11 | 0·50 | 0·34 |
| **2018** | 110 | 169·5 | 0·65 | 0·35 | 0·30 | 0·14 | 0·51 | 0·35 |
| **2019** | 110 | 169·5 | 0·65 | 0·38 | 0·27 | 0·12 | 0·53 | 0·37 |
| **2020** | 146 | 174·0 | 0·84 | 0·45 | 0·39 | 0·11 | 0·72 | 0·49 |
| **2021** | 106 | 173·5 | 0·61 | 0·36 | 0·25 | 0·07 | 0·54 | 0·33 |
| **2022** | 115 | 166·0 | 0·69 | 0·43 | 0·26 | 0·08 | 0·61 | 0·42 |
| **2023** | 94 | 164·5 | 0·57 | 0·30 | 0·27 | 0·08 | 0·49 | 0·33 |
| ^1^ Epidemiologic year (June-July), the number of the year is presented as the year from June-December. ^2^ The mean number of live births per two year (number of live births epidemiologic year 1987 is mean number 1987 and 1988). Incidence numbers presented as incidence per 1,000 live births.  EOD = early onset disease (0-6 days), LOD = late onset disease (7-89 days). | | | | | | | | |

**Supplementary Figure 1 – Distribution of Group B Streptococcal disease by age.**


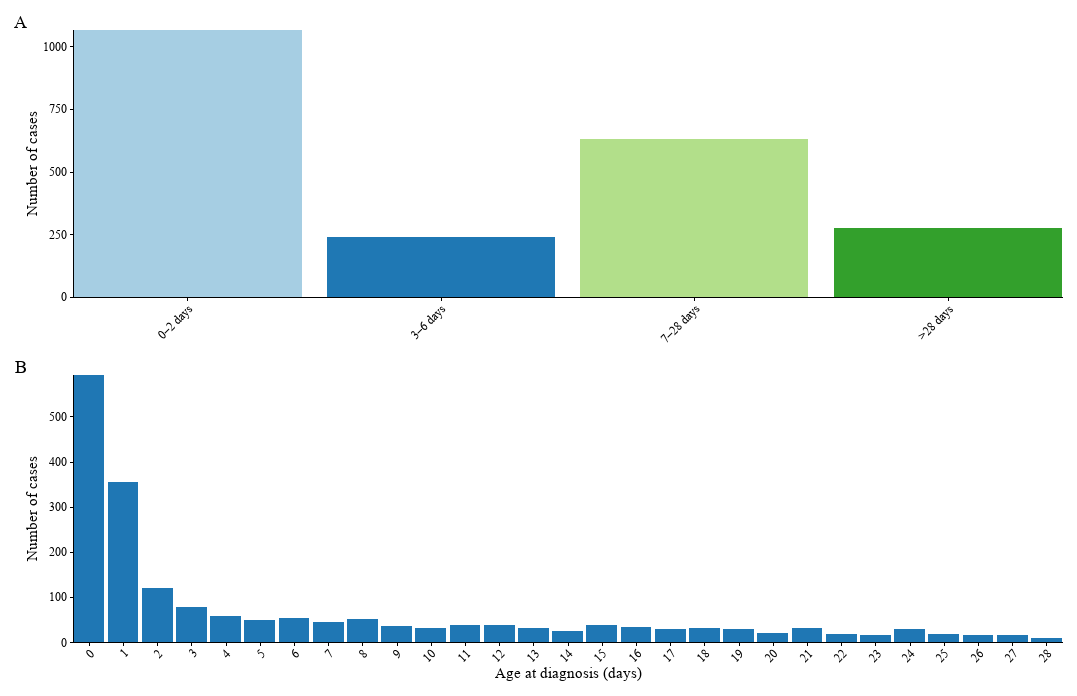


(A) Age of disease onset distribution of cases in the first 28 days of life. The histogram shows the number of cases diagnosed at each day of age. (B) Distribution of cases by age groups: 0–2 days, 3–6 days, 7–28 days and >28 days.

**Supplementary Table 2 - Serotype, clonal complex and virulence factor distribution of Group B Streptococcal isolates per year of infection and source of infection.**

|  | **1987–1991** | | | **1992–1996** | | | **1997–2001** | | | **2002–2006** | | |
| --- | --- | --- | --- | --- | --- | --- | --- | --- | --- | --- | --- | --- |
|  | **Total**  N=133 | **Sepsis**  N=56 | **Meningitis**  N=77 | **Total**  N=162 | **Sepsis**  N=77 | **Meningitis**  N=85 | **Total**  N=232 | **Sepsis**  N=139 | **Meningitis**  N=93 | **Total**  N=245 | **Sepsis**  N=151 | **Meningitis**  N=94 |
| **Serotype** |  |  |  |  |  |  |  |  |  |  |  |  |
| Ia | 4 (3%) | 3 (5%) | 1 (1%) | 21 (13%) | 11 (14%) | 10 (12%) | 43 (19%) | 34 (24%) | 9 (10%) | 45 (18%) | 34 (23%) | 11 (12%) |
| Ib | 16 (12%) | 11 (20%) | 5 (6%) | 9 (6%) | 5 (6%) | 4 (5%) | 10 (4%) | 8 (6%) | 2 (2%) | 10 (4%) | 7 (5%) | 3 (3%) |
| II | 8 (6%) | 6 (11%) | 2 (3%) | 5 (3%) | 5 (6%) | 0 (0%) | 10 (4%) | 10 (7%) | 0 (0%) | 15 (6%) | 14 (9%) | 1 (1%) |
| III | 100 (75%) | 32 (57%) | 68 (88%) | 104 (64%) | 39 (51%) | 65 (76%) | 138 (59%) | 60 (43%) | 78 (84%) | 150 (61%) | 76 (50%) | 74 (79%) |
| IV | 1 (1%) | 1 (2%) | 0 (0%) | 1 (1%) | 1 (1%) | 0 (0%) | 6 (3%) | 5 (4%) | 1 (1%) | 4 (2%) | 4 (3%) | 0 (0%) |
| V | 0 (0%) | 0 (0%) | 0 (0%) | 5 (3%) | 4 (5%) | 1 (1%) | 16 (7%) | 13 (9%) | 3 (3%) | 9 (4%) | 7 (5%) | 2 (2%) |
| VI | 0 (0%) | 0 (0%) | 0 (0%) | 0 (0%) | 0 (0%) | 0 (0%) | 0 (0%) | 0 (0%) | 0 (0%) | 1 (0%) | 1 (1%) | 0 (0%) |
| VII | 0 (0%) | 0 (0%) | 0 (0%) | 0 (0%) | 0 (0%) | 0 (0%) | 0 (0%) | 0 (0%) | 0 (0%) | 1 (0%) | 1 (1%) | 0 (0%) |
| VIII | 0 (0%) | 0 (0%) | 0 (0%) | 0 (0%) | 0 (0%) | 0 (0%) | 0 (0%) | 0 (0%) | 0 (0%) | 0 (0%) | 0 (0%) | 0 (0%) |
| IX | 0 (0%) | 0 (0%) | 0 (0%) | 0 (0%) | 0 (0%) | 0 (0%) | 0 (0%) | 0 (0%) | 0 (0%) | 0 (0%) | 0 (0%) | 0 (0%) |
| NT | 4 (3%) | 3 (5%) | 1 (1%) | 17 (10%) | 12 (16%) | 5 (6%) | 9 (4%) | 9 (6%) | 0 (0%) | 10 (4%) | 7 (5%) | 3 (3%) |
|  | **2007–2011** | | | **2012–2015** | | | **2016–2019** | | | **2020–2023** | | |
|  | **Total**  N=294 | **Sepsis**  N=229 | **Meningitis**  N=65 | **Total**  N=262 | **Sepsis**  N=192 | **Meningitis**  N=70 | **Total**  N=382 | **Sepsis**  N=300 | **Meningitis**  N=82 | **Total**  N=453 | **Sepsis**  N=394 | **Meningitis**  N=59 |
| **Serotype** |  |  |  |  |  |  |  |  |  |  |  |  |
| Ia | 81 (28%) | 65 (28%) | 16 (25%) | 48 (18%) | 39 (20%) | 9 (13%) | 72 (19%) | 61 (20%) | 11 (13%) | 69 (15%) | 63 (16%) | 6 (10%) |
| Ib | 9 (3%) | 8 (3%) | 1 (2%) | 19 (7%) | 17 (9%) | 2 (3%) | 20 (5%) | 18 (6%) | 2 (2%) | 14 (3%) | 13 (3%) | 1 (2%) |
| II | 13 (4%) | 12 (5%) | 1 (2%) | 13 (5%) | 13 (7%) | 0 (0%) | 26 (7%) | 24 (8%) | 2 (2%) | 33 (7%) | 33 (8%) | 0 (0%) |
| III | 169 (57%) | 122 (53%) | 47 (72%) | 160 (61%) | 103 (54%) | 57 (81%) | 228 (60%) | 162 (54%) | 66 (80%) | 267 (59%) | 218 (55%) | 49 (83%) |
| IV | 6 (2%) | 6 (3%) | 0 (0%) | 10 (4%) | 10 (5%) | 0 (0%) | 7 (2%) | 7 (2%) | 0 (0%) | 15 (3%) | 15 (4%) | 0 (0%) |
| V | 14 (5%) | 14 (6%) | 0 (0%) | 10 (4%) | 9 (5%) | 1 (1%) | 23 (6%) | 22 (7%) | 1 (1%) | 39 (9%) | 37 (9%) | 2 (3%) |
| VI | 1 (0%) | 1 (0%) | 0 (0%) | 2 (1%) | 1 (1%) | 1 (1%) | 2 (1%) | 2 (1%) | 0 (0%) | 7 (2%) | 7 (2%) | 0 (0%) |
| VII | 1 (0%) | 1 (0%) | 0 (0%) | 0 (0%) | 0 (0%) | 0 (0%) | 1 (0%) | 1 (0%) | 0 (0%) | 0 (0%) | 0 (0%) | 0 (0%) |
| VIII | 0 (0%) | 0 (0%) | 0 (0%) | 0 (0%) | 0 (0%) | 0 (0%) | 1 (0%) | 1 (0%) | 0 (0%) | 0 (0%) | 0 (0%) | 0 (0%) |
| IX | 0 (0%) | 0 (0%) | 0 (0%) | 0 (0%) | 0 (0%) | 0 (0%) | 2 (1%) | 2 (1%) | 0 (0%) | 9 (2%) | 8 (2%) | 1 (2%) |
| NT | 0 (0%) | 0 (0%) | 0 (0%) | 0 (0%) | 0 (0%) | 0 (0%) | 0 (0%) | 0 (0%) | 0 (0%) | 0 (0%) | 0 (0%) | 0 (0%) |
|  | **1987–1991** | | | **1992–1996** | | | **1997–2001** | | | **2002–2006** | | |
|  | **Total**  N=151 | **Sepsis**  N=62 | **Meningitis**  N=89 | **Total**  N=157 | **Sepsis**  N=75 | **Meningitis**  N=82 | **Total**  N=227 | **Sepsis**  N=136 | **Meningitis**  N=91 | **Total**  N=240 | **Sepsis**  N=150 | **Meningitis**  N=90 |
| **Alp** |  |  |  |  |  |  |  |  |  |  |  |  |
| *alp1* | 21 (14%) | 15 (24%) | 6 (7%) | 24 (15%) | 15 (20%) | 9 (11%) | 37 (16%) | 30 (22%) | 7 (8%) | 41 (17%) | 30 (20%) | 11 (12%) |
| *alp2/3* | 2 (1%) | 1 (2%) | 1 (1%) | 6 (4%) | 3 (4%) | 3 (4%) | 15 (7%) | 13 (10%) | 2 (2%) | 10 (4%) | 9 (6%) | 1 (1%) |
| *alphaC* | 29 (19%) | 15 (24%) | 14 (16%) | 22 (14%) | 14 (19%) | 8 (10%) | 33 (15%) | 29 (21%) | 4 (4%) | 33 (14%) | 25 (17%) | 8 (9%) |
| *rib* | 99 (66%) | 31 (50%) | 68 (76%) | 104 (66%) | 42 (56%) | 62 (76%) | 141 (62%) | 64 (47%) | 77 (85%) | 153 (64%) | 84 (56%) | 69 (77%) |
| None | 0 (0%) | 0 (0%) | 0 (0%) | 1 (1%) | 1 (1%) | 0 (0%) | 1 (0%) | 0 (0%) | 1 (1%) | 3 (1%) | 2 (1%) | 1 (1%) |
|  | **2007–2011** | | | **2012–2015** | | | **2016–2019** | | | **2020–2023** | | |
|  | **Total**  N=284 | **Sepsis**  N=222 | **Meningitis**  N=62 | **Total**  N=252 | **Sepsis**  N=182 | **Meningitis**  N=70 | **Total**  N=174 | **Sepsis**  N=140 | **Meningitis**  N=34 | **Total**  N=238 | **Sepsis**  N=207 | **Meningitis**  N=31 |
| **Alp** |  |  |  |  |  |  |  |  |  |  |  |  |
| *alp1* | 70 (25%) | 58 (26%) | 12 (19%) | 42 (17%) | 36 (20%) | 6 (9%) | 27 (16%) | 23 (16%) | 4 (12%) | 29 (12%) | 25 (12%) | 4 (13%) |
| *alp2/3* | 11 (4%) | 10 (5%) | 1 (2%) | 10 (4%) | 9 (5%) | 1 (1%) | 8 (5%) | 8 (6%) | 0 (0%) | 10 (4%) | 10 (5%) | 0 (0%) |
| *alphaC* | 24 (8%) | 20 (9%) | 4 (6%) | 29 (12%) | 24 (13%) | 5 (7%) | 27 (16%) | 27 (19%) | 0 (0%) | 46 (19%) | 44 (21%) | 2 (6%) |
| *rib* | 179 (63%) | 134 (60%) | 45 (73%) | 170 (67%) | 112 (62%) | 58 (83%) | 108 (62%) | 81 (58%) | 27 (79%) | 149 (63%) | 124 (60%) | 25 (81%) |
| None | 0 (0%) | 0 (0%) | 0 (0%) | 1 (0%) | 1 (1%) | 0 (0%) | 4 (2%) | 1 (1%) | 3 (9%) | 4 (2%) | 4 (2%) | 0 (0%) |
|  | **1987–1991** | | | **1992–1996** | | | **1997–2001** | | | **2002–2006** | | |
|  | **Total**  N=151 | **Sepsis**  N=62 | **Meningitis**  N=89 | **Total**  N=157 | **Sepsis**  N=75 | **Meningitis**  N=82 | **Total**  N=227 | **Sepsis**  N=136 | **Meningitis**  N=91 | **Total**  N=240 | **Sepsis**  N=150 | **Meningitis**  N=90 |
| **Clonal complex** |  |  |  |  |  |  |  |  |  |  |  |  |
| CC1 | 13 (9%) | 9 (15%) | 4 (4%) | 12 (8%) | 10 (13%) | 2 (2%) | 24 (11%) | 22 (16%) | 2 (2%) | 19 (8%) | 18 (12%) | 1 (1%) |
| CC12 | 22 (15%) | 11 (18%) | 11 (12%) | 12 (8%) | 7 (9%) | 5 (6%) | 20 (9%) | 18 (13%) | 2 (2%) | 21 (9%) | 14 (9%) | 7 (8%) |
| CC17 | 39 (26%) | 9 (15%) | 30 (34%) | 51 (32%) | 15 (20%) | 36 (44%) | 79 (35%) | 32 (24%) | 47 (52%) | 101 (42%) | 51 (34%) | 50 (56%) |
| CC19 | 61 (40%) | 22 (35%) | 39 (44%) | 54 (34%) | 28 (37%) | 26 (32%) | 59 (26%) | 30 (22%) | 29 (32%) | 52 (22%) | 31 (21%) | 21 (23%) |
| CC23 | 16 (11%) | 11 (18%) | 5 (6%) | 26 (17%) | 14 (19%) | 12 (15%) | 41 (18%) | 32 (24%) | 9 (10%) | 44 (18%) | 33 (22%) | 11 (12%) |
| CC other | 0 (0%) | 0 (0%) | 0 (0%) | 2 (1%) | 1 (1%) | 1 (1%) | 4 (2%) | 2 (1%) | 2 (2%) | 3 (1%) | 3 (2%) | 0 (0%) |
|  | **2007–2011** | | | **2012–2015** | | | **2016–2019** | | | **2020–2023** | | |
|  | **Total**  N=284 | **Sepsis**  N=222 | **Meningitis**  N=62 | **Total**  N=252 | **Sepsis**  N=182 | **Meningitis**  N=70 | **Total**  N=174 | **Sepsis**  N=140 | **Meningitis**  N=34 | **Total**  N=238 | **Sepsis**  N=207 | **Meningitis**  N=31 |
| **Clonal complex** |  |  |  |  |  |  |  |  |  |  |  |  |
| CC1 | 20 (7%) | 20 (9%) | 0 (0%) | 23 (9%) | 20 (11%) | 3 (4%) | 10 (6%) | 10 (7%) | 0 (0%) | 26 (11%) | 26 (13%) | 0 (0%) |
| CC12 | 8 (3%) | 8 (4%) | 0 (0%) | 18 (7%) | 16 (9%) | 2 (3%) | 8 (5%) | 8 (6%) | 0 (0%) | 12 (5%) | 11 (5%) | 1 (3%) |
| CC17 | 116 (41%) | 80 (36%) | 36 (58%) | 115 (46%) | 73 (40%) | 42 (60%) | 85 (49%) | 63 (45%) | 22 (65%) | 119 (50%) | 98 (47%) | 21 (68%) |
| CC19 | 60 (21%) | 51 (23%) | 9 (15%) | 42 (17%) | 29 (16%) | 13 (19%) | 25 (14%) | 20 (14%) | 5 (15%) | 29 (12%) | 25 (12%) | 4 (13%) |
| CC23 | 74 (26%) | 58 (26%) | 16 (26%) | 50 (20%) | 40 (22%) | 10 (14%) | 41 (24%) | 34 (24%) | 7 (21%) | 47 (20%) | 43 (21%) | 4 (13%) |
| CC other | 6 (2%) | 5 (2%) | 1 (2%) | 4 (2%) | 4 (2%) | 0 (0%) | 5 (3%) | 5 (4%) | 0 (0%) | 5 (2%) | 4 (2%) | 1 (3%) |
|  | **1987–1991** | | | **1992–1996** | | | **1997–2001** | | | **2002–2006** | | |
|  | **Total**  N=151 | **Sepsis**  N=62 | **Meningitis**  N=89 | **Total**  N=157 | **Sepsis**  N=75 | **Meningitis**  N=82 | **Total**  N=227 | **Sepsis**  N=136 | **Meningitis**  N=91 | **Total**  N=240 | **Sepsis**  N=150 | **Meningitis**  N=90 |
| **Pilus island** |  |  |  |  |  |  |  |  |  |  |  |  |
| PI-1 | 4 (3%) | 3 (5%) | 1 (1%) | 5 (3%) | 3 (4%) | 2 (2%) | 5 (2%) | 5 (4%) | 0 (0%) | 5 (2%) | 4 (3%) | 1 (1%) |
| PI-1 & PI-2A | 83 (55%) | 33 (53%) | 50 (56%) | 69 (44%) | 36 (48%) | 33 (40%) | 92 (41%) | 58 (43%) | 34 (37%) | 83 (35%) | 55 (37%) | 28 (31%) |
| PI-1 & PI-2B | 41 (27%) | 9 (15%) | 32 (36%) | 53 (34%) | 17 (23%) | 36 (44%) | 85 (37%) | 39 (29%) | 46 (51%) | 103 (43%) | 53 (35%) | 50 (56%) |
| PI-2A | 16 (11%) | 11 (18%) | 5 (6%) | 25 (16%) | 14 (19%) | 11 (13%) | 40 (18%) | 31 (23%) | 9 (10%) | 45 (19%) | 34 (23%) | 11 (12%) |
| PI-2B | 0 (0%) | 0 (0%) | 0 (0%) | 0 (0%) | 0 (0%) | 0 (0%) | 2 (1%) | 0 (0%) | 2 (2%) | 0 (0%) | 0 (0%) | 0 (0%) |
| None | 7 (5%) | 6 (10%) | 1 (1%) | 5 (3%) | 5 (7%) | 0 (0%) | 3 (1%) | 3 (2%) | 0 (0%) | 4 (2%) | 4 (3%) | 0 (0%) |
|  | **2007–2011** | | | **2012–2015** | | | **2016–2019** | | | **2020–2023** | | |
|  | **Total**  N=284 | **Sepsis**  N=222 | **Meningitis**  N=62 | **Total**  N=252 | **Sepsis**  N=182 | **Meningitis**  N=70 | **Total**  N=174 | **Sepsis**  N=140 | **Meningitis**  N=34 | **Total**  N=238 | **Sepsis**  N=207 | **Meningitis**  N=31 |
| **Pilus island** |  |  |  |  |  |  |  |  |  |  |  |  |
| PI-1 | 10 (4%) | 8 (4%) | 2 (3%) | 14 (6%) | 12 (7%) | 2 (3%) | 7 (4%) | 7 (5%) | 0 (0%) | 16 (7%) | 16 (8%) | 0 (0%) |
| PI-1 & PI-2A | 77 (27%) | 68 (31%) | 9 (15%) | 63 (25%) | 48 (26%) | 15 (21%) | 37 (21%) | 31 (22%) | 6 (18%) | 44 (18%) | 39 (19%) | 5 (16%) |
| PI-1 & PI-2B | 117 (41%) | 82 (37%) | 35 (56%) | 92 (37%) | 57 (31%) | 35 (50%) | 57 (33%) | 44 (31%) | 13 (38%) | 64 (27%) | 53 (26%) | 11 (35%) |
| PI-2A | 74 (26%) | 59 (27%) | 15 (24%) | 52 (21%) | 42 (23%) | 10 (14%) | 45 (26%) | 39 (28%) | 6 (18%) | 52 (22%) | 47 (23%) | 5 (16%) |
| PI-2B | 3 (1%) | 2 (1%) | 1 (2%) | 26 (10%) | 18 (10%) | 8 (11%) | 26 (15%) | 19 (14%) | 7 (21%) | 57 (24%) | 47 (23%) | 10 (32%) |
| None | 3 (1%) | 3 (1%) | 0 (0%) | 5 (2%) | 5 (3%) | 0 (0%) | 2 (1%) | 0 (0%) | 2 (6%) | 5 (2%) | 5 (2%) | 0 (0%) |
| Data are shown as n (%).  Abbreviations: NT = non-typeable, CC = clonal complex, PI = pilus island protein, | | | | | | | | | | | | |

**Supplementary Table 3 - Virulence factor distribution between sepsis and meningitis isolates.**

|  | **Overall**,  N = 1,723 | **Meningitis**,  N = 549 | **Sepsis**,  N = 1,174 | **p value^1^** |
| --- | --- | --- | --- | --- |
| **Clonal complex** |  |  |  |  |
| CC1 | 147 (9%) | 12 (2%) | 135 (11%) | <0·0001 |
| CC12 | 121 (7%) | 28 (5%) | 93 (8%) | 0·03 |
| CC17 | 705 (41%) | 284 (52%) | 421 (36%) | <0·0001 |
| CC19 | 382 (22%) | 146 (27%) | 236 (20%) | <0·01 |
| CC23 | 339 (20%) | 74 (13%) | 265 (23%) | <0·0001 |
| CC other | 29 (2%) | 5 (1%) | 24 (2%) | 0·10 |
| **Pilus island** |  |  |  |  |
| PI-1 | 66 (4%) | 8 (1%) | 58 (5%) | <0·001 |
| PI-1 & PI-2A | 548 (32%) | 180 (33%) | 368 (31%) | 0·55 |
| PI-1 & PI-2B | 612 (36%) | 258 (47%) | 354 (30%) | <0·0001 |
| PI-2A | 349 (20%) | 72 (13%) | 277 (24%) | <0·0001 |
| PI-2B | 114 (7%) | 28 (5%) | 86 (7%) | 0·08 |
| None | 34 (2%) | 3 (1%) | 31 (3%) | 0·008 |
| ***hvgA*** |  |  |  |  |
| Positive | 708 (41%) | 283 (52%) | 425 (36%) | <0·0001 |
| Negative | 1,015 (59%) | 266 (48%) | 749 (64%) | <0·0001 |
| ***Srr*** |  |  |  |  |
| *srr1* | 933 (54%) | 244 (44%) | 689 (59%) | <0·0001 |
| *srr2* | 705 (41%) | 282 (51%) | 423 (36%) | <0·0001 |
| Negative | 85 (5%) | 23 (4%) | 62 (5%) | 0·49 |
| ***cylE*** |  |  |  |  |
| Positive | 1,717 (100%) | 547 (100%) | 1,170 (100%) | 0·94 |
| Negative | 6 (0%) | 2 (0%) | 4 (0%) | 0·94 |
| ***lmb*** |  |  |  |  |
| Positive | 1,698 (99%) | 542 (99%) | 1,156 (98%) | 0·68 |
| Negative | 25 (1%) | 7 (1%) | 18 (2%) | 0·68 |
| ***fbsA*** |  |  |  |  |
| Positive | 1,268 (74%) | 389 (71%) | 879 (75%) | 0·08 |
| Negative | 455 (26%) | 160 (29%) | 295 (25%) | 0·08 |
| ***fbsB*** |  |  |  |  |
| Positive | 1,697 (98%) | 546 (99%) | 1,151 (98%) | 0·04 |
| Negative | 26 (2%) | 3 (1%) | 23 (2%) | 0·04 |
| ***fbsC*** |  |  |  |  |
| Positive | 1,718 (100%) | 548 (100%) | 1,170 (100%) | 0·58 |
| Negative | 5 (0%) | 1 (0%) | 4 (0%) | 0·58 |
| ***iagA*** |  |  |  |  |
| Positive | 1,723 (100%) | 549 (100%) | 1,174 (100%) | NA |
| Negative | 0 (0%) | 0 (0%) | 0 (0%) | NA |
| ***pbsP*** |  |  |  |  |
| Positive | 1,711 (99%) | 544 (99%) | 1,167 (99%) | 0·47 |
| Negative | 12 (1%) | 5 (1%) | 7 (1%) | 0·47 |
| Data are shown as n (%). ^1^ Meningitis versus sepsis with Fisher’s Exact test.  Abbreviations: CC = clonal complex, PI = pilus island protein, HvgA = hypervirulent GBS adhesin protein, Srr = Serine-rich repeat protein, CylE = β-hemolysin/cytolysin exporter protein E, Lmb = laminin-binding protein, Fbs = fibrinogen-binding protein, IagA = invasion-associated gene A, PbsP = plasminogen-binding surface protein. Gene names are shown in italics; pilus islands are shown in uppercase. | | | | |

**Supplementary Table 4 - Serotype and virulence factor distribution among the clonal complex groups.**

|  | **CC1**  N = 147 | **CC12**  N = 121 | **CC17**  N = 705 | **CC19**  N = 382 | **CC23**  N = 339 | **CC other**  N = 29 |
| --- | --- | --- | --- | --- | --- | --- |
| **Serotype** |  |  |  |  |  |  |
| Ia | 12 (8%) | 2 (2%) | 2 (0%) | 0 (0%) | 283 (86%) | 9 (31%) |
| Ib | 16 (11%) | 64 (57%) | 1 (0%) | 3 (1%) | 3 (1%) | 0 (0%) |
| II | 24 (17%) | 18 (16%) | 0 (0%) | 39 (10%) | 3 (1%) | 4 (14%) |
| III | 4 (3%) | 6 (5%) | 700 (99%) | 308 (81%) | 14 (4%) | 1 (3%) |
| IV | 30 (21%) | 2 (2%) | 1 (0%) | 0 (0%) | 6 (2%) | 0 (0%) |
| V | 35 (25%) | 6 (5%) | 0 (0%) | 23 (6%) | 13 (4%) | 7 (24%) |
| VI | 11 (8%) | 0 (0%) | 0 (0%) | 0 (0%) | 0 (0%) | 0 (0%) |
| VII | 1 (1%) | 0 (0%) | 0 (0%) | 0 (0%) | 0 (0%) | 1 (3%) |
| VIII | 0 (0%) | 0 (0%) | 0 (0%) | 1 (0%) | 0 (0%) | 0 (0%) |
| IX | 1 (1%) | 1 (1%) | 0 (0%) | 0 (0%) | 0 (0%) | 5 (17%) |
| Non-typeable | 8 (6%) | 14 (12%) | 1 (0%) | 7 (2%) | 7 (2%) | 2 (7%) |
| **Alp** |  |  |  |  |  |  |
| *alp1* | 51 (35%) | 0 (0%) | 0 (0%) | 12 (3%) | 218 (64%) | 10 (34%) |
| *alp2/3* | 58 (39%) | 0 (0%) | 0 (0%) | 0 (0%) | 14 (4%) | 0 (0%) |
| *alphaC* | 35 (24%) | 119 (98%) | 0 (0%) | 4 (1%) | 70 (21%) | 15 (52%) |
| *rib* | 3 (2%) | 2 (2%) | 702 (100%) | 364 (95%) | 32 (9%) | 0 (0%) |
| None | 0 (0%) | 0 (0%) | 3 (0%) | 2 (1%) | 5 (1%) | 4 (14%) |
| **Pilus island** |  |  |  |  |  |  |
| PI-1 | 41 (28%) | 4 (3%) | 9 (1%) | 6 (2%) | 5 (1%) | 1 (3%) |
| PI-1 & PI-2A | 67 (46%) | 108 (89%) | 0 (0%) | 362 (95%) | 11 (3%) | 0 (0%) |
| PI-1 & PI-2B | 16 (11%) | 4 (3%) | 584 (83%) | 0 (0%) | 0 (0%) | 8 (28%) |
| PI-2A | 0 (0%) | 3 (2%) | 0 (0%) | 12 (3%) | 315 (93%) | 19 (66%) |
| PI-2B | 0 (0%) | 0 (0%) | 107 (15%) | 0 (0%) | 6 (2%) | 1 (3%) |
| None | 23 (16%) | 2 (2%) | 5 (1%) | 2 (1%) | 2 (1%) | 0 (0%) |
| ***hvgA*** |  |  |  |  |  |  |
| Positive | 0 (0%) | 0 (0%) | 702 (99%) | 0 (0%) | 6 (2%) | 0 (0%) |
| Negative | 147 (100%) | 121 (100%) | 3 (<1%) | 382 (100%) | 333 (98%) | 29 (100%) |
| ***Srr*** |  |  |  |  |  |  |
| *srr1* | 145 (99%) | 115 (95%) | 0 (0%) | 317 (83%) | 328 (97%) | 28 (97%) |
| *srr2* | 0 (0%) | 0 (0%) | 699 (99%) | 0 (0%) | 6 (2%) | 0 (0%) |
| Negative | 2 (1%) | 6 (5%) | 6 (1%) | 65 (17%) | 5 (1%) | 1 (3%) |
| ***cylE*** |  |  |  |  |  |  |
| Positive | 145 (99%) | 119 (98%) | 705 (100%) | 381 (99%) | 339 (100%) | 28 (97%) |
| Negative | 2 (1%) | 2 (2%) | 0 (0%) | 1 (<1%) | 0 (0%) | 1 (3%) |
| ***lmb*** |  |  |  |  |  |  |
| Positive | 143 (97%) | 119 (98%) | 700 (99%) | 371 (97%) | 337 (99%) | 28 (97%) |
| Negative | 4 (3%) | 2 (2%) | 5 (1%) | 11 (3%) | 2 (1%) | 1 (3%) |
| ***fbsA*** |  |  |  |  |  |  |
| Positive | 116 (79%) | 114 (94%) | 677 (96%) | 6 (2%) | 331 (98%) | 24 (83%) |
| Negative | 31 (21%) | 7 (6%) | 28 (4%) | 376 (98%) | 8 (2%) | 5 (17%) |
| ***fbsB*** |  |  |  |  |  |  |
| Positive | 144 (98%) | 118 (98%) | 705 (100%) | 382 (100%) | 339 (100%) | 9 (31%) |
| Negative | 3 (2%) | 3 (2%) | 0 (0%) | 0 (0%) | 0 (0%) | 20 (69%) |
| ***fbsC*** |  |  |  |  |  |  |
| Positive | 147 (100%) | 120 (99%) | 701 (99%) | 382 (100%) | 339 (100%) | 29 (100%) |
| Negative | 0 (0%) | 1 (1%) | 4 (1%) | 0 (0%) | 0 (0%) | 0 (0%) |
| ***iagA*** |  |  |  |  |  |  |
| Positive | 147 (100%) | 121 (100%) | 705 (100%) | 382 (100%) | 339 (100%) | 29 (100%) |
| Negative | 0 (0%) | 0 (0%) | 0 (0%) | 0 (0%) | 0 (0%) | 0 (0%) |
| ***pbsP*** |  |  |  |  |  |  |
| Positive | 147 (100%) | 109 (90%) | 705 (100%) | 382 (100%) | 339 (100%) | 29 (100%) |
| Negative | 0 (0%) | 12 (10%) | 0 (0%) | 0 (0%) | 0 (0%) | 0 (0%) |
| Data are shown as n (%).  Abbreviations: CC = clonal complex, Alp = Alpha-like protein, PI = pilus island protein, hvgA = hypervirulent GBS adhesin protein, Srr = Serine-rich repeat protein, cylE = β-hemolysin/cytolysin exporter protein E, lmb = laminin-binding protein, fbs = fibrinogen-binding protein, iagA = invasion-associated gene A, pbsP = plasminogen-binding surface protein. Gene names are shown in italics; pilus islands are shown in uppercase. | | | | | | |

**Supplementary Table 5 - Pilus island distribution across most common clonal complex groups and epidemiologic year of infection, in groups**

|  | **1987–1991** | | | | | | **1992–1996** | | | | | |
| --- | --- | --- | --- | --- | --- | --- | --- | --- | --- | --- | --- | --- |
|  | **CC1**  N=13 | **CC12**  N=22 | **CC17**  N=39 | **CC19**  N=61 | **CC23**  N=16 | **CCoth**  N=0 | **CC1**  N=12 | **CC12**  N=12 | **CC17**  N=51 | **CC19**  N=54 | **CC23**  N=26 | **CCoth**  N=2 |
| **Pilus island** |  |  |  |  |  |  |  |  |  |  |  |  |
| PI-1 | 2 (15%) | 1 (5%) | 0 (0%) | 1 (2%) | 0 (0%) | 0 (0%) | 2 (17%) | 0 (0%) | 0 (0%) | 2 (4%) | 0 (0%) | 1 (50%) |
| PI-1 & PI-2A | 2 (15%) | 21 (95%) | 0 (0%) | 60 (98%) | 0 (0%) | 0 (0%) | 4 (33%) | 11 (92%) | 0 (0%) | 52 (96%) | 2 (8%) | 0 (0%) |
| PI-1 & PI-2B | 2 (15%) | 0 (0%) | 39 (100%) | 0 (0%) | 0 (0%) | 0 (0%) | 2 (17%) | 0 (0%) | 51 (100%) | 0 (0%) | 0 (0%) | 0 (0%) |
| PI-2A | 0 (0%) | 0 (0%) | 0 (0%) | 0 (0%) | 16 (100%) | 0 (0%) | 0 (0%) | 0 (0%) | 0 (0%) | 0 (0%) | 24 (92%) | 1 (50%) |
| PI-2B | 0 (0%) | 0 (0%) | 0 (0%) | 0 (0%) | 0 (0%) | 0 (0%) | 0 (0%) | 0 (0%) | 0 (0%) | 0 (0%) | 0 (0%) | 0 (0%) |
| None | 7 (54%) | 0 (0%) | 0 (0%) | 0 (0%) | 0 (0%) | 0 (0%) | 4 (33%) | 1 (8%) | 0 (0%) | 0 (0%) | 0 (0%) | 0 (0%) |
|  | **1997–2001** | | | | | | **2002–2006** | | | | | |
|  | **CC1**  N=24 | **CC12**  N=20 | **CC17**  N=79 | **CC19**  N=59 | **CC23**  N=41 | **CCoth**  N=4 | **CC1**  N=19 | **CC12**  N=21 | **CC17**  N=101 | **CC19**  N=52 | **CC23**  N=44 | **CCoth**  N=3 |
| **Pilus island** |  |  |  |  |  |  |  |  |  |  |  |  |
| PI-1 | 4 (17%) | 0 (0%) | 0 (0%) | 0 (0%) | 1 (2%) | 0 (0%) | 4 (21%) | 0 (0%) | 0 (0%) | 1 (2%) | 0 (0%) | 0 (0%) |
| PI-1 & PI-2A | 12 (50%) | 18 (90%) | 0 (0%) | 58 (98%) | 4 (10%) | 0 (0%) | 10 (53%) | 21 (100%) | 0 (0%) | 51 (98%) | 1 (2%) | 0 (0%) |
| PI-1 & PI-2B | 5 (21%) | 2 (10%) | 77 (97%) | 0 (0%) | 0 (0%) | 1 (25%) | 1 (5%) | 0 (0%) | 101 (100%) | 0 (0%) | 0 (0%) | 1 (33%) |
| PI-2A | 0 (0%) | 0 (0%) | 0 (0%) | 1 (2%) | 36 (88%) | 3 (75%) | 0 (0%) | 0 (0%) | 0 (0%) | 0 (0%) | 43 (98%) | 2 (67%) |
| PI-2B | 0 (0%) | 0 (0%) | 2 (3%) | 0 (0%) | 0 (0%) | 0 (0%) | 0 (0%) | 0 (0%) | 0 (0%) | 0 (0%) | 0 (0%) | 0 (0%) |
| None | 3 (12%) | 0 (0%) | 0 (0%) | 0 (0%) | 0 (0%) | 0 (0%) | 4 (21%) | 0 (0%) | 0 (0%) | 0 (0%) | 0 (0%) | 0 (0%) |
|  | **2007–2011** | | | | | | **2012–2015** | | | | | |
|  | **CC1**  N=20 | **CC12**  N=8 | **CC17**  N=116 | **CC19**  N=60 | **CC23**  N=74 | **CCoth**  N=6 | **CC1**  N=23 | **CC12**  N=18 | **CC17**  N=115 | **CC19**  N=42 | **CC23**  N=50 | **CCoth**  N=4 |
| **Pilus island** |  |  |  |  |  |  |  |  |  |  |  |  |
| PI-1 | 6 (30%) | 0 (0%) | 3 (3%) | 0 (0%) | 1 (1%) | 0 (0%) | 12 (52%) | 2 (11%) | 0 (0%) | 0 (0%) | 0 (0%) | 0 (0%) |
| PI-1 & PI-2A | 10 (50%) | 7 (88%) | 0 (0%) | 59 (98%) | 1 (1%) | 0 (0%) | 9 (39%) | 15 (83%) | 0 (0%) | 38 (90%) | 1 (2%) | 0 (0%) |
| PI-1 & PI-2B | 2 (10%) | 0 (0%) | 110 (95%) | 0 (0%) | 0 (0%) | 5 (83%) | 1 (4%) | 1 (6%) | 90 (78%) | 0 (0%) | 0 (0%) | 0 (0%) |
| PI-2A | 0 (0%) | 0 (0%) | 0 (0%) | 1 (2%) | 72 (97%) | 1 (17%) | 0 (0%) | 0 (0%) | 0 (0%) | 2 (5%) | 47 (94%) | 3 (75%) |
| PI-2B | 0 (0%) | 0 (0%) | 3 (3%) | 0 (0%) | 0 (0%) | 0 (0%) | 0 (0%) | 0 (0%) | 25 (22%) | 0 (0%) | 0 (0%) | 1 (25%) |
| None | 2 (10%) | 1 (12%) | 0 (0%) | 0 (0%) | 0 (0%) | 0 (0%) | 1 (4%) | 0 (0%) | 0 (0%) | 2 (5%) | 2 (4%) | 0 (0%) |
|  | **2016–2019** | | | | | | **2020–2023** | | | | | |
|  | **CC1**  N=10 | **CC12**  N=8 | **CC17**  N=85 | **CC19**  N=25 | **CC23**  N=41 | **CCoth**  N=5 | **CC1**  N=26 | **CC12**  N=12 | **CC17**  N=119 | **CC19**  N=29 | **CC23**  N=47 | **CCoth**  N=5 |
| **Pilus island** |  |  |  |  |  |  |  |  |  |  |  |  |
| PI-1 | 2 (20%) | 1 (12%) | 2 (2%) | 0 (0%) | 2 (5%) | 0 (0%) | 9 (35%) | 0 (0%) | 4 (3%) | 2 (7%) | 1 (2%) | 0 (0%) |
| PI-1 & PI-2A | 8 (80%) | 4 (50%) | 0 (0%) | 24 (96%) | 1 (2%) | 0 (0% | 12 (46%) | 11 (92%) | 0 (0%) | 20 (69%) | 1 (2%) | 0 (0%) |
| PI-1 & PI-2B | 0 (0%) | 1 (12%) | 55 (65%) | 0 (0%) | 0 (0%) | 1 (20%) | 3 (12%) | 0 (0%) | 61 (51%) | 0 (0%) | 0 (0%) | 0 (0%) |
| PI-2A | 0 (0%) | 2 (25%) | 0 (0%) | 1 (4%) | 38 (93%) | 4 (80%) | 0 (0%) | 1 (8%) | 0 (0%) | 7 (24%) | 39 (83%) | 5 (100%) |
| PI-2B | 0 (0%) | 0 (0%) | 26 (31%) | 0 (0%) | 0 (0%) | 0 (0%) | 0 (0%) | 0 (0%) | 51 (43%) | 0 (0%) | 6 (13%) | 0 (0%) |
| None | 0 (0%) | 0 (0%) | 2 (2%) | 0 (0%) | 0 (0%) | 0 (0%) | 2 (8%) | 0 (0%) | 3 (3%) | 0 (0%) | 0 (0%) | 0 (0%) |
| Data are shown as n (%).  Abbreviations: CC = clonal complex, CCoth = CC other, PI = pilus island protein. | | | | | | | | | | | | |

**Supplementary File 1 - Targeted literature search**

Search for reviews:

((GBS[Title/Abstract]) OR (agalactiae[Title/Abstract])) AND (meningitis[Title/Abstract]), filter review (n=139)

((GBS[Title/Abstract]) OR (agalactiae[Title/Abstract])) AND (meningitis[Title/Abstract]) AND (virulence[Title/Abstract]), filter review (n=16)

Selected 6 relevant reviews below + Maisey 2009 as lateral search

|  | **Megli^1^** | **Manuel^2^** | **Liu^3^** | **Furuta^4^** | **Armistead^5^** | **Pietrocola^6^** | **Maisey^7^** | N ✔= |
| --- | --- | --- | --- | --- | --- | --- | --- | --- |
| **HvgA** | ✔ | ✔ | ✔ | ✔ | ✔ | ✔ |  | 6 |
| **Srr2** | ✔ | ✔ |  | ✔ |  | ✔ |  | 4 |
| **IagA** |  | ✔ |  |  |  |  | ✔ | 2 |
| **BspC** |  |  |  | ✔ |  |  |  | 1 |
| **PbsP** | ✔ | ✔ |  |  | ✔ | ✔ |  | 4 |
| **CylE** |  | ✔ |  |  | ✔ |  | ✔ | 3 |
| **PI-1/2A/2B** | ✔ | ✔ |  |  | ✔ |  | ✔ | 4 |
| **ScpB** |  |  |  |  |  |  |  | 0 |
| **Lmb** |  | ✔ | ✔ |  | ✔ |  | ✔ | 4 |
| **FbsA/B/C** |  |  | ✔ |  | ✔ |  | ✔ | 3 |
| **Blr** |  |  |  | ✔ |  |  |  | 1 |
| **BibA** |  |  |  |  |  |  |  | 0 |
| **SfbA** |  |  |  |  | ✔ |  |  | 1 |

✔ means a remark associating the virulence factor (positively or negatively) with meningitis was in the review.

Selected GBS virulence factors associated with neonatal GBS meningitis:

| **Factor / Protein** | **Gene name** | **Function** |
| --- | --- | --- |
| HvgA | *hvgA* | Hypervirulent adhesin specific to CC17; promotes intestinal and BBB crossing, associated with late-onset meningitis |
| Srr2 | *srr2* | Serine-rich repeat adhesin; binds fibrinogen and promotes adhesion to brain endothelium; enriched in CC17 strains |
| IagA | *iagA* | Glycosyltransferase required for proper LTA anchoring; essential for BBB invasion |
| PbsP | *pbsP* | Plasminogen-binding surface protein; promotes BMEC invasion and meningitis in mice |
| CylE (β-hemolysin/cytolysin) | *cylE* | β-hemolysin/cytolysin; pore-forming toxin; enhances BBB penetration and contributes to lethality |
| Pilus island 1, 2A, 2B | PI (PI-1, PI-2A, PI-2B) | Pilus structural genes; mediate adhesion and invasion of epithelial and endothelial cells |
| Lmb | *lmb* | Laminin-binding protein; promotes adhesion to endothelial cells; contributes to neurotropism |
| FbsA, B and C | *fbsA*, *fbsB* and *fbsC* | Fibrinogen-binding surface protein; contributes to BMEC invasion and brain colonization |

**References**

1. Megli CJ, Carlin SM, Giacobe EJ, Hillebrand GH, Hooven TA. Virulence and pathogenicity of group B Streptococcus: Virulence factors and their roles in perinatal infection. *Virulence* 2025;**16**(1):2451173.

2. Manuel G, Twentyman J, Noble K, et al. Group B streptococcal infections in pregnancy and early life. *Clin Microbiol Rev* 2025;**38**(1):e0015422.

3. Liu Y, Liu J. Group B Streptococcus: Virulence Factors and Pathogenic Mechanism. *Microorganisms* 2022;**10**(12)

4. Furuta A, Brokaw A, Manuel G, et al. Bacterial and Host Determinants of Group B Streptococcal Infection of the Neonate and Infant. *Front Microbiol* 2022;**13**:820365.

5. Armistead B, Oler E, Adams Waldorf K, Rajagopal L. The Double Life of Group B Streptococcus: Asymptomatic Colonizer and Potent Pathogen. *J Mol Biol* 2019;**431**(16):2914–2931.

6. Pietrocola G, Arciola CR, Rindi S, Montanaro L, Speziale P. Streptococcus agalactiae Non-Pilus, Cell Wall-Anchored Proteins: Involvement in Colonization and Pathogenesis and Potential as Vaccine Candidates. *Front Immunol* 2018;**9**:602.

7. Maisey HC, Doran KS, Nizet V. Recent advances in understanding the molecular basis of group B Streptococcus virulence. *Expert Rev Mol Med* 2008;**10**:e27.

**Supplementary File 2 - Custom virulence database**

>1__lmb__VFG001330__1 VFG001330(gb|WP_000755138) (lmb) laminin-binding surface protein [Lmb (VF0275) - Adherence (VFC0001)] [Streptococcus agalactiae 2603V/R]

ATGAAAAAAGTTTTTTTTCTCATGGCTATGGTTGTGAGTTTAGTAATGATAGCAGGGTGTGATAAGTCAGCAAACCCCAAACAGCCTACGCAAGGCATGTCAGTTGTAACCAGCTTTTACCCAATGTATGCGATGACAAAAGAAGTATCTGGAGACCTAAATGATGTGAGGATGATCCAATCAGGTGCAGGCATTCATTCCTTTGAACCGTCTGTAAATGATGTGGCAGCTATTTATGACGCGGATTTGTTTGTTTACCATTCACATACCTTAGAAGCTTGGGCAAGGGATCTAGACCCTAATTTAAAAAAATCAAAGGTTAATGTGTTTGAAGCGTCAAAACCTCTGACACTAGATAGAGTCAAAGGGCTAGAAGATATGGAAGTCACACAAGGCATTGACCCTGCGACACTTTATGACCCACATACCTGGACGGATCCCGTTTTAGCTGGTGAGGAAGCTGTTAATATCGCTAAAGAGCTAGGACATTTGGATCCTAAACACAAAGACAGTTACACTAAAAAGGCTAAGGCTTTCAAAAAAGAAGCAGAGCAACTAACTGAAGAATACACTCAAAAATTTAAAAAGGTGCGCTCAAAAACATTTGTGACGCAACACACGGCATTTTCTTATCTGGCTAAACGATTCGGCTTGAAACAACTTGGTATCTCGGGTATTTCTCCAGAGCAAGAGCCCTCTCCTCGCCAATTGAAAGAAATTCAAGACTTTGTTAAAGAATACAACGTCAAGACTATTTTTGCAGAAGACAACGTCAACCCCAAAATTGCTCATGCTATTGCGAAATCAACAGGAGCTAAAGTAAAGACATTAAGTCCACTTGAAGCTGCTCCAAGCGGAAACAAGACATATCTAGAAAATCTTAGAGCAAATTTGGAAGTGCTCTATCAACAGTTGAAGTAA

>2__cylE__VFG001332__3 VFG001332(gb|WP_000650743) (cylE) haemolysin CylE [-haemolysin/cytolysin (VF0279) - Exotoxin (VFC0235)] [Streptococcus agalactiae 2603V/R]

ATGAAAGATGATAATAAATTAAAGATTTCTGAAGCTTCCTTAGAAGATTATTCTGAAGTGGTTCATTTATTTAATAGGAATCATGTTTATCAATTTCCAGACGGTAGGCCTTTAACTGTTGATGACCTTGATTTAACGTTAAAAGTTAAAGAAGTGACACATTTATTTTTACTGAAAAATCATGGTGTTTTGATTGGAACATCAGCTTTCTTTAAATTTATTACCTATGGTTGTTTAGATTGGAATAGTAGCTTTAGTGGTTTTCTTTTAATTGATTCGAAAAGTCGTAGTGGACAGGCAATCACTTATCTATATAAAACTATCTTAAAGAAGATAACAAAACTGAAGTTTTCTAATATCTATACCGAAATCAGTAACTACAATAAACCCTCCTTAGCTTTATCAAAATTGAATGGCTTCAAAGAGTATGATAAGACATATGAAGACATACTGCATTGTCGATCATTGCGTAGTCACCTCCCTAAGATATTGAATACGTTTCGTATTTCGAATTATTATGGTAAAACATACGACATATCAACTTTTCAAATTATGGAGGAAATTGAAAATCCCTTGGAAGAGGAGACAGAAATTAGGACCAAAGTCTCTGATGAAGAAATATTATTTAAAGCAGAAGATAGTGCTTCACTTCCTTATTATTTAAAAATGAGCTTGTTTCAAATGGAAATTGCTAAGTTAGATAACCGTTATGTTTTACAAGTTGACTTTTTATCGGAACAAGTAAAGAGGGTTCGGGTTAAAACTGGAAAATATCACTTGGCAAACTTAACGAGGGCTCACCCTAGCCTAACTTTAAGTCGTTTTGCCAATTATTATTACATTCAAGCAACTGTGGAAACCCTTTATGGTAATATCGATGTTCAACTGGAACGTCGAAAAAAGCATTATAGAGATGCAACTATTTGTCTTAAGAGAACTTTTCAAGGTTATGATTTACTTATCTCTCCAAATGGCAGCCTTATTTTTGAAAAGCAGAAAAGAAAGATACTTGAAGATAGTTTTTTGATTTTTAGCCAACCTTTGGACAAGAAATTAGTTGTAAAAGAAGAAGAAAATCACATTACAATCAAATGTTTCTATCAAGGTGCTTTAATTGAGAAAATCATGACATTTACAAGTGACGAAGAAATCACTTGTGTTTATAAATGTAATCAAAAGGCTAAAGAGATGTTCCCAAAATTATTGAAACAGACTTTCAAGTTACACTGCCAAGAGCAGTTGATTAGAGATAGTGAAGGTTATCTTGTGAATGTTCCAGGAAGTTACCCGATTGAGCATGATGATTTTCTTCGAGCAGATAAGTTTGAAGATAGACAATTCCATTATTACCTCCCGAATGAAGATAAAATGATTTCCTATTCTCCTCCTGGCAAAGCCAGCAATCAAATGCAATTTAGACCCTTATGTCTTATTGATACAGATAGCCTTTCTTTTCCACTGACTTATCATTTTAGGATTTCACAAGCATCATCGGAGGAGGCGTTAATCAATCTAAAAAAACAGCCAATATGGGACAGCAACTACCAAGCGTCAGCAACTGACCTTTTAAAACATATCTCTAACCTAACATTAGAAGAAGAGAAGGATTATGGCATCAAGAGAATGATAGCTAACAGAAAGCATTATCCTAGTCACAAACTGGTTTTAGCCTATAATCAGATTGTTTTGCCTAAGAATGAAATTCCAAGAGACAGTGAATTATATTCTATTTCTTTTGATTATCGAATTAGAGGAAAGTTTGTCCAAATCCGCCAAGGGGAACATGTCAAATATGATAATAAATCCTATGTTTTGGAAAATAGTCAACAATTAGTATTGTATGTAGCTAGCGATGATAAATATATACTAATTTCTGCTAAAAATGGAATATTTTATTCTTACAAAGAGAACAATCATTTAAAAATAAGGTGCATGTTTAAAAGGAACTCACCTTATGCTACAAATGTGAGCATTACAGAATATAGAAAGTGTGAAGAAAAATGA

>3__fbsA__VFG001796__5 VFG001796(gb|WP_000482192) (fbsA) fibrinogen receptor FbsA [FbsA (VF0282) - Adherence (VFC0001)] [Streptococcus agalactiae NEM316]

TTGTTCAATAAAATAGGTTTTAGAACTTGGAAATCAGGAAAGCTTTGGCTTTATATGGGAGTGCTAGGATCAACTATTATTTTAGGATCAAGTCCTGTATCTGCTATGGATAGTGTTGGAAATCAAAGTCAGGGCAATGTTTTAGAGCGTCGTCAACGTGATGCAGAAAACAGAAGCCAAGGCAATGTTCTAGAGCGTCGTCAACGCGATGCAGAAAACAGAAGCCAAGGTAATGTTCTAGAGCGTCGTCAACGTGATGCAGAAAACAGAAGCCAAGGTAATGTTCTAGAGCGTCGTCAACGTGATGCAGAAAACAGAAGCCAAGGTAATGTTCTAGAGCGTCGTCAACGCGATGTTGAGAATAAGAGCCAAGGCAATGTTTTAGAGCGTCGTCAACGTGATGCGGAAAACAAGAGCCAAGGCAATGTTTTAGAGCGTCGTCAACGTGATGCAGAAAACAGAAGCCAAGGCAATGTTTTAGAGCGTCGTCAACGCGATGTTGAGAATAAGAGCCAAGGCAATGTTTTAGAGCGTCGTCAACGTGATGCAGAAAACAGAAGCCAAGGTAATGTTCTAGAGCGTCGTCAACGCGATGTTGAGAATAAGAGCCAAGGTAATGTTCTAGAGCGTCGTCAACGCGATGTTGAGAATAAGAGCCAAGGCAATGTTTTAGAGCGTCGTCAACGTGATGCAGAAAACAGAAGCCAAGGTAATGTTCTAGAGCGTCGTCAACGCGATGTTGAGAATAAGAGCCAAGGTAATGTTCTAGAGCGTCGTCAACGCGATGTTGAGAATAAGAGCCAAGGCAATGTTTTAGAGCGTCGTCAACGTGATGCAGAAAACAGAAGCCAAGGCAATGTTCTAGAGCGTCGTCAACGCGATGCAGAAAACAGAAGCCAAGGTAATGTTCTAGAGCGTCGTCAACGTGATGCGGAAAACAAGAGCCAAGTAGGTCAACTTATAGGGAAAAATCCACTTCTTTCAAAGTCAATTATATCTAGAGAAAATAATCACTCTAGTCAAGGTGACTCTAACAAACAGTCATTCTCTAAAAAAGTATCTCAGGTTACTAATGTAGCTAATAGACCGATGTTAACTAATAATTCTAGAACAATTTCAGTGATAAATAAATTACCTAAAACAGGTGATGATCAAAATGTCATTTTTAAACTTGTAGGTTTTGGTTTAATTTTGTTAACAAGTCGCTGCGGTTTGAGACGCAATGAAAATTAA

>4__fbsA__VFG005211__6 VFG005211(gb|WP_000482175) (fbsA) fibrinogen receptor FbsA [FBPs (VF0243) - Adherence (VFC0001)] [Streptococcus agalactiae A909]

TTGTTCAATAAAATAGGTTTTAGAACTTGGAAATCAGGAAAGCTTTGGCTTTATATGGGAGTGCTAGGATCAACTATTATTTTAGGATCAAGTCCTGTATCTGCTATGGATAGTGTTGGAAATCAAAGTCAAGGTAATGTTCTAGAGCGTCGTCAACGTGATGCGGATAACAAGAGCCAAGGCAATGTTCTAGAACGTCGTCAACGCGATGTAGAAAACAGAAGCCAAGGCAATGTTCTAGAGCGTCGTCAACGCGATGCGGATAACAAGAGCCAAGGCAATGTTTTAGAGCGCCGCCAACGCGATGCAGAAAACAAAAGTCAGGGCAATGTTCTAGAACGTCGTCAACGTGATGTTGAGAATAAGAGCCAAGGCAATGTTCTAGAGCGTCGCCAACGTGATGCAGAAAACAAAAGTCAGGGTAATGTTCTAGAGCGTCGTCAACGCGATGCAGATAACAAGAGCCAAGGTAATGTTCTAGAACGTCGTCAACGCGATGTGGAAAACAAAAGTCAGGGCAATGTTCTAGAACGTCGTCAACGTGATGTTGAGAATAAGAGCCAAGGCAATGTTCTAGAGCGTCGCCAACGTGATGCAGAAAACAAAAGTCAGGGTAATGTTCTAGAGCGTCGTCAACGCGATGCAGATAACAAGAGCCAAGGTAATGTTCTAGAACGTCGTCAACGCGATGTGGAAAACAAAAGTCAGGGCAATGTTCTAGAGCGTCGCCAACGTGATGTTGAGAACAAGAGCCAAGTAGGTCAACTTATAGGGAAAAATCCACTTCTTTCAAAGTCAACTATATCTAGAGAAAATAATCACTCTAGTCAAGGTGACTCTAACAAACAGTCATTCTCTAAAAAAGTATCTCAGGTTACTAATGTAGCTAATAGACCAATGTTAACTAATAATTCTAGAACAATTTCAGTGATAAATAAATTACCTAAAACAGGTGATGATCAAAATGTCATTTTTAAACTTGTAGGTTTTGGTTTAATTTTGTTAACAAGTCGCTGCGGTTTGAGACGCAATGAAAATTAA

>5__fbsB__VFG005214__7 VFG005214(gb|WP_000743864) (fbsB) Fibrinogen-binding surface protein B [FBPs (VF0243) - Adherence (VFC0001)] [Streptococcus agalactiae A909]

ATGAAAAAACAATTTTTAAAATCAGCAGCGATTCTATCGCTAGCAGTAACAGCAGTATCTACAAGTCAGCCGGTAGGGGCGATAGTGGGAAAAGATGAAACGAAATTAAGACAGCAACTTGGGTATATTGATTCAAAAAAATCTGGAAAGAAAATTGATGAGAGATGGGGTGAAAAAATTTATAACTATCTAAGTTATGAGCTCATTGAAGCAAACGAATGGATTAACCGAAGTGAGTTTCAGGAACCTGAGTATAGAACTATTTTATCAGAATTTAAAGATAAAATAGATAGTATTGAATACTACTTAATTAACTTAAGTAATATTGCTAAAGAAGATGCGCATCAAAGAAATATTCTTCAATCATTAGATAAATATGAAAAAAGTGGAATATATAATTTAGACCAAGGTGTTTATAATTATATATATCAAGAAATTAGTTCAGCGAAGCACAAGTTTTCGGATGGGGTAGATAAAATTTATCGTTTAGATAGTACTTTATTTCCTTTCTCTGTATGGTATGATAAACATTTAGATAATAATGATAACTACAAGGATAATAAAGATTTTAAAGAGTACATTGCTTTATTAAATGAAATTACACGAAAAGCAAGGTTAGGATATCAAATTGTAAATAACCATAAAGATGGGGAGCATAAAGATGAAGCAGAAATACTGGATATTTTAATCAGAGATATAACTTTTGTTTCAAAAGATGCTCCTGGATATAAATATATTCCAAATAAAAGAATTGCAGCGAAAATAATTGAAGATTTAGATGGAGTTATCAATGACTTTTTTAAGAATACAGGAAAAGATAAGCCTTCTCTTGAAAAGTTAAAGGACACAGAATTCCATAAAAAATATCTTAATTCAACTGAACCGTATAGTATTGAAACTAACTTACCTTCAAATTATAAAGAATTGAAGGAAAAGCAAATAAAAAAATTGGAATATGGATACAAAAAGTCTTCTAAAATTTATACGTCTGCTCATTATGCACTATATAGTGAAGAAATTGATGCTGCAAAAGAATTGCTTCAAAAAGTGAAGATTGCAAAAGATAATTATAATGAAATTAAGTCCATGAATCTAAGCCCTTCAATTTTTAATCAGTATTTACAACTTCTTCAAATAGTTATAAGTTCAGAGATAAATCTAAAAAAGGCACTCGATAACACTGTGGATTTACCCATAGAAAATAATTTTAATACATTGGACATTCAATATAATAAATTAGACACTGCTATTAAGTCACTTCGAAAATTTGTAACTAAGTATAAACAAGAAGTAAGAAAAGCTACTAAGAGTTATTCTAAAAAAGAATTAGTAAATGCAGAGTTAACAAAAGTAATTTCTAATGACAATATTTTATTAGACATGCAAGCAATTAGTTCCAATTATGGGTCTACTAAAAAGTTTGTTTATTCAGTCAAAAGATTACCTTACGTCCCGCAAGTAATTATGACAACAACGTCTAATGTACTTATGCCTCAAAAGCAAGTTGAAAAAGTGAAGCTACTCACCCCATTTACAATTTCTAATAAAGAAGTATTGAATCATGACTCACTAGTAGAAAATGATGCTCAAAAACAAAAAGTAGAACAGGAAAAAACTAAATCGCTAGCTCCTCAAAAAGGGGCTGTAAAAGAACAAACTGAGCAAAAAGTATCTGGAAATACTCAAGAGATAGAAAAGAAATCTGAAACTGTGGCAACTCCACAACAAAGTTCAGTTGCGCAAACTTCTGTCCAACAGCCGGCTCCGGTTCAATCAGTTGTTCAAGAATCCAAAGCTTCTCAAGAGGAGATTAATGCAGCACACGATGCTATTTCGGCGTATAAATCAACAGTCAATATTGCTAATACAGCCGGTGTAACAACTGCGGAAATGACCACGCTCATTAATACTCAAACTTCTAATCTTTCTGATGTTGAGAAAGCTTTAGGAAATAATAAGGTTAATAATGGTGCAGTCAATGTATTGAGAGAAGATACAGCTCGTCTTGAGAATATGATTTGGAATCGTGCTTACCAAGCTATTGAAGAATTCAACGTCGCTCGTAATACTTATAATAACCAAATCAAGACAGAAACAGTTCCAGTTGATAATGATATTGAAGCTATTTTAGCAGGTTCTCAAGCTAAAATTAGCCATTTGGACAATCGTATCGGAGCGCGCCACATGGATCAAGCTTTTGTAGCTAGTTTATTAGAAGTTACTGAGATGAGTAAATCAATCTCATCGCGTATAAAAGAGTAG

>6__fbsB__VFG005215__9 VFG005215(gb|WP_000743858) (fbsB) Fibrinogen-binding surface protein B [FbsB (VF0523) - Adherence (VFC0001)] [Streptococcus agalactiae NEM316]

ATGAAAAAACAATTTTTAAAATCAGCAGCGATTCTATCGCTAGCAGTAACAGCAGTATCTACAAGTCAGCCGGTAGCCGGGATAACTAAAGATTATAATAACCGAAATGAAAAAGTAAAAAAGTATTTACAAGAAAATAATTTCGGTCATAAAATAGCGTATGGATGGAAAAATAAAGTAGAATTTGATTTTCGTTATTTATTGGATACTGCTAAATATTTAGTAAATAAAGAAGAATTTCAAGATCCTTTATATAATGATGCGCGCGAAGAATTGATAAGTTTTATTTTTCCTTATGAGAAATTTTTAATTAACAATCGTGACATAACTAAATTAACAGTTAATCAGTATGAAGCGATTGTGAATAGAATGAGTGTTGCTTTACAAAAATTTTCAAAGAATATTTTTGAGAAACAGAAAGTAAATAAAGATTTAATCCCTATTGCGTTTTGGATTGAGAAAAGTTACAGAACTGTTGGAACGAATGAAATCGCCGCTTCTGTAGGCATTCAAGGAGGATTTTATCAAAACTTCCATGATTATTATAATTATTCATATCTATTAAATTCTTTATGGCATGAAGGAAATGTAAAAGAAGTAGTTAAGGATTATGAAAACACTATTCGTCAAATACTATCTAAAAAGCATGAGATTGAAAAAATTCTTAATCAGAGCACTTCTGATATCTCTATAGATGATGATGATTACGAAAAAGGAAATAAAGAATTGCTAAGGGAAAAATTAAATATTATTCTAAATCTTTCAAAGAGAGATTACAGAGTAACTCCATACTATGAAGTGAATAAACTACATACAGGGCTTATTTTATTGGAGGATGTCCCTAATTTAAAGATTGCTAAGGATAAGTTGTTCTCATTAGAGAATTCTTTAAAGGAATACAAAGGAGAGAAAGTTAATTATGAGGAACTAAGATTCAATACGGAACCTTTAACTAGTTACTTAGAAAATAAAGAAAAATTTTTAGTCCCCAATATTCCATATAAAAATAAATTAATTTTAAGGGAAGAAGATAAATATAGTTTTGAAGATGATGAAGAAGAGTTTGGAAATGAACTTCTAAGTTACAATAAGCTTAAGAATGAAGTTTTACCTGTTAATATTACAACTTCTACTATATTAAAACCGTTTGAACAGAAGAAAATTGTGGAAGATTTTAATCCTTATTCTAATTTAGACAATTTAGAAATAAAAAAAATAAGGTTGAATGGCTCCCAAAAACAAAAAGTAGAACAGGAAAAAACTAAATCGCCAACTCCTCAAAAAGAGACTGTGAAAGAACAAACTGAGCAAAAAGTATCTGGAAATACTCAAGAGGTAGAAAAGAAATCTGAAACTGTGGCAACTTCACAACAAAGTTCAGTTGCGCAAACTTCTGTCCAACAGCCGGCTCCGGTTCAATCAGTTGTTCAAGAATCCAAAGCTTCTCAAGAGGAGATTAATGCAGCACACGATGCTATTTCGGCGTATAAATCAACAGTCAATATTGCTAATACAGCCGGTGTAACAACTGCGGAAATGACCACGCTCATTAATACTCAAACTTCTAATCTTTCTGATGTTGAGAAAGCTTTAGGAAATAATAAGGTTAATAATGGTGCAGTCAATGTATTGAGAGAAGATACAGCTCGTCTTGAGAATATGATTTGGAATCGTGCTTACCAAGCTATTGAAGAATTCAACGTCGCTCGTAATACTTATAATAACCAAATCAAGACAGAAACAGTTCCAGTTGATAATGATATTGAAGCTATTTTAGCAGGTTCTCAAGCTAAAATTAGCCATTTGGACAATCGTATCGGAGCGCGCCACATGGATCAAGCTTTTGTAGCTAGTTTATTAGAAGTTACTGAGATGAGTAAATCAATCTCATCGCGTATAAAAGAGTAG

>5__fbsB__VFG005216__8 VFG005216(gb|WP_000743861) (fbsB) Fibrinogen-binding surface protein B [FBPs (VF0243) - Adherence (VFC0001)] [Streptococcus agalactiae 2603V/R]

ATGAAAAAACAATTTTTAAAATCAGCAGCGATTCTATCGCTAGCAGTAACAGCAGTATCTACAAGTCAGCCGGTAGGGGCGATAGTGGGAAAAGATGAAACGAAATTAAGACAGCAACTTGGGTATATTGATTCAAAAAAATCTGGAAAGAAAATTGATGAGAGATGGGGTGAAAAAATTTATAACTATCTAAGTTATGAGCTCATTGAAGCAAACGAATGGATTAACCGAAGTGAGTTTCAGGAACCTGAGTATAGAACTATTTTATCAGAATTTAAAGATAAAATAGATAGTATTGAATACTACTTAATTAACTTAAGTAATATTGCTAAAGAAGATGCGCATCAAAGAAATATTCTTCAATCATTAGATAAATATGAAAAAAGTGGAATATATAATTTAGACCAAGGTGTTTATAATTATATATATCAAGAAATTAGTTCAGCGAAGCACAAGTTTTCGGATGGGGTAGATAAAATTTATCGTTTAGATAGTACTTTATTTCCTTTCTCTGTATGGTATGATAAACATTTAGATAATAATGATAACTACAAGGATAATAAAGATTTTAAAGAGTACATTGCTTTATTAAATGAAATTACACGAAAAGCAAGGTTAGGATATCAAATTGTAAATAACCATAAAGATGGGGAGCATAAAGATGAAGCAGAAATACTGGATATTTTAATCAGAGATATAACTTTTGTTTCAAAAGATGCTCCTGGATATAAATATATTCCAAATAAAAGAATTGCAGCGAAAATAATTGAAGATTTAGATGGAATTATCAATGACTTTTTTAAGAATACAGGAAAAGATAAGCCTTCTCTTGAAAAGTTAAAGGACACAGAATTCCATAAAAAATATCTTAATTCAACTGAACCGTATAGTATTGAAACTAACTTACCTTCAAATTATAAAGAATTGAAGGAAAAGCAAATAAAAAAATTGGAATATGGATACAAAAAGTCTTCTAAAATTTATACGTCTGCTCATTATGCACTATATAGTGAAGAAATTGATGCTGCAAAAGAATTGCTTCAAAAAGTGAAGATTGCAAAAGATAATTATAATGAAATTAAGTCCATGAATCTAAGCCCTTCAATTTTTAATCAGTATTTACAACTTCTTCAAATAGTTATAAGTTCAGAGATAAATCTAAAAAAGGCACTCGATAACACTGTGGATTTACCCATAGAAAATAATTTTAATACATTGGACATTCAATATAATAAATTAGACACTGCTATTAAGTCACTTCGAAAATTTGTAACTAAGTATAAACAAGAAGTAAGAAAAGCTACTAAGAGTTATTCTAAAAAAGAATTAGTAAATGCAGAGTTAACAAAAGTAATTTCTAATGACAATATTTTATTAGACATGCAAGCAATTAGTTCCAATTATGGGTCTACTAAAAAGTTTGTTTATTCAGTCAAAAGATTACCTTACGTCCCGCAAGTAATTATGACAACAACGTCTAATGTACTTATGCCTCAAAAGCAAGTTGAAAAAGTGAAGCTACTCACCCCATTTACAATTTCTAATAAAGAAGTATTGAATCATGACTCACTAGTAGAAAATGATGCTCAAAAACAAAAAGTAGAACAGGAAAAAACTAAATCGCTAGCTCCTCAAAAAGGGGCTGTAAAAGAACAAACTGAGCAAAAAGTATCTGGAAATACTCAAGAGATAGAAAAGAAATCTGAAACTGTGGCAACTCCACAACAAAGTTCAGTTGCGCAAACTTCTGTCCAACAGCCGGCTCCGGTTCAATCAGTTGTTCAAGAATCCAAAGCTTCTCAAGAGGAGATTAATGCAGCACACGATGCTATTTCGGCGTATAAATCAACAGTCAATATTGCTAATACAGCCGGTGTAACAACTGCGGAAATGACCACGCTCATTAATACTCAAACTTCTAATCTTTCTGATGTTGAGAAAGCTTTAGGAAATAATAAGGTTAATAATGGTGCAGTCAATGTATTGAGAGAAGATACAGCTCGTCTTGAGAATATGATTTGGAATCGTGCTTACCAAGCTATTGAAGAATTCAACGTCGCTCGTAATACTTATAATAACCAAATCAAGACAGAAACAGTTCCAGTTGATAATGATATTGAAGCTATTTTAGCAGGTTCTCAAGCTAAAATTAGCCATTTGGACAATCGTATCGGAGCGCGCCACATGGATCAAGCTTTTGTAGCTAGTTTATTAGAAGTTACTGAGATGAGTAAATCAATCTCATCGCGTATAAAAGAGTAG

>1__lmb__VFG005230__2 VFG005230(gb|WP_000715197) (lmb) laminin-binding surface protein [Lmb (VF0275) - Adherence (VFC0001)] [Streptococcus agalactiae NEM316]

ATGAAAAAAGGTTTTTTTCTCATGGCTATGGTTGTGAGTTTAGTAATGATAGCAGGGTGTGATAAGTCAGCAAACCCCAAACAGCCTACGCAAGGCATGTCAGTTGTAACCAGCTTTTACCCAATGTATGCGATGACAAAAGAAGTATCTGGAGACCTCAATGATGTGAGGATGATCCAATCAGGTGCAGGCATTCATTCCTTTGAACCGTCTGTAAATGATGTGGCAGCTATTTATGACGCGGATTTGTTTGTTTACCATTCACATACCTTAGAAGCTTGGGCAAGGGATCTAGACCCTAATTTAAAAAAATCAAAGGTTGATGTGTTTGAAGCGTCAAAACCTTTGACACTAGATAGAGTCAAAGGGCTAGAAGATATGGAAGTCACACAAGGCATTGACCCTGCGACACTTTATGACCCACATACCTGGACGGATCCCGTTTTAGCTGGTGAGGAAGCTGTTAATATCGCTAAAGAGCTAGGACGTTTGGATCCTAAACACAAAGACAGTTACACTAAAAAGGCTAAGGCTTTCAAAAAAGAAGCAGAGCAACTAACTGAAGAATACACTCAAAAATTTAAAAAGGTGCGCTCAAAAACATTCGTGACGCAACACACGGCATTTTCTTATCTGGCTAAACGATTCGGCTTGAAACAACTTGGTATCTCGGGTATTTCTCCAGAGCAAGAGCCCTCTCCTCGCCAATTGAAAGAAATTCAAGACTTTGTCAAAGAATACAACGTCAAGACTATTTTTGCAGAAGACAACGTCAATCCCAAAATTGCTCATGCTATTGCGAAATCAACAGGAGCTAAAGTAAAGACATTAAGTCCACTTGAAGCTGCTCCAAGCGGAAACAAGACATATCTAGAAAATCTTAGAGCAAATTTGGAAGTGCTCTATCAACAGTTGAAGTAA

>2__cylE__VFG005781__4 VFG005781(gb|WP_000650746) (cylE) haemolysin CylE [Beta-haemolysin/cytolysin (VF0279) - Exotoxin (VFC0235)] [Streptococcus agalactiae NEM316]

ATGAAAGATGATAATAAATTAAAGATTTCTGAAGCTTCCTTAGAAGATTATTCTGAAGTGGTTCATTTATTTAATAGGAATCATGTTTATCAATTTCCAGACGGTAGGCCTTTAACTGTTGATGACCTTGATTTAACGTTAAAAGTTAAAGAAGTGACACATTTATTTTTACTGAAAAATCATGGTGTTTTGATTGGAACATCAGCTTTCTTTAAATTTATTACCTATGGTTGTTTAGATTGGAATAGTAGCTTTAGTGGTTTTCTTTTAATTGATTCGAAAAGTCGTAGTGGACAGGCAATCACTTATCTATATAAAACTATCTTAAAGAAGATAACAAAACTGAAGTTTTCTAATATCTATACCGAAATCAGTAACTACAATAAACCCTCCTTAGCTTTATCAAAATTGAATGGCTTCAAAGAGTATGATAAGACATATGAAGACATACTGCATTGTCGATCATTGCGTAGTCACCTCCCTAAGATATTGAATACGTTTCGTATTTCGAATTATTATGGTAAAACATACGACATATCAACTTTTCAAATTATGGAGGAAATTGAAAATCCCTTGGAAGAGGAGACAGAAATTAGGACCAAAGTCTCTGATGAAGAAATATTATTTAAAGCAGAAGATAGTGCTTCACTTCCTTATTATTTAAAAATGAGCTTGTTTCAAATGGAAATTGCTAGGTTAGATAACCGTTATGTTTTACAAGTTGACTTTTTATCGGAACAAGTAAAGAGGGTTCGGGTTAAAACTGGAAAATATCACTTGGCAAACTTAACGAGGGCTCACCCTAGCCTAACTTTAAGTCGTTTTGCCAATTATTATTACATTCAAGCAACTGTGGAAACCCTTTATGGTAATATCGATGTTCAACTGGAACGTCGAAAAAAGCATTATAGAGATGCAACTATTTGTCTTAAGAGAACTTTTCAAGGTTATGATTTACTTATCTCTCCAAATGGCAGCCTTATTTTTGAAAAGCAGAAAAGAAAGATACTTGAAGATAGTTTTTTGATTTTTAGCCAACCTTTGGACAAGAAATTAGTTGTAAAAGAAGAAGAAAATCACATTACAATCAAATGTTTCTATCAAGGTGCTTTAATTGAGAAAATCATGACATTTACAAGTGACGAAGAAATCACTTGTGTTTATAAATGTAATCAAAAGGCTAAAGAGATGTTCCCAAAATTATTGAAACAGACTTTCAAGTTACACTGCCAAGAGCAGTTGATTAGAGATAGTGAAGGTTATCTTGTGAATGTTCCAGGAAGTTACCCGATTGAGCATGATGATTTTCTTCGAGCAGATAAGTTTGAAGATAGACAATTCCATTATTACCTCCCGAATGAAGATAAAATGATTTCCTATTCTCCTCCTGGCAAAGCCAGCAATCAAATGCAATTTAGACCCTTATGTCTTATTGATACAGATAGCCTTTCTTTTCCACTGACTTATCATTTTAGGATTTCACAAGCATCATCGGAGGAGGCGTTAATCAATCTAAAAAAACAGCCAATATGGGACAGCAACTACCAAGCGTCAGCAACTGACCTTTTAAAACATATCTCTAACCTAACATTAGAAGAAGAGAAGGATTATGGCATCAAGAGAATGATAGCTAACAGAAAGCATTATCCTAGTCACAAACTGGTTTTAGCCTATAATCAGATTGTTTTGCCTAAGAATGAAATTCCAAGAGACAGTGAATTATATTCTATTTCTTTTGATTATCGAATTAGAGGAAAGTTTGTCCAAATCCGCCAAGGGGAACATGTCAAATATGATAATAAATCCTATGTTTTGGAAAATAGTCAACAATTAGTATTGTATGTAGCTAGCGATGATAAATATATACTAATTTCTGCTAAAAATGGAATATTTTATTCTTACAAAGAGAACAATCATTTAAAAATAAGGTGCATGTTTAAAAGGAACTCACCTTATGCTACAAATGTGAGCATTACAGAATATAGAAAGTGTGAAGAAAAATGA

>7__iagA__SS1_00801__10 SS1_00801 Glycosyltransferase LafB, responsible for the formation of Gal-Glc-DAG,glycosyltransferase, MSMEG_0565 family,Glycosyl transferases group 1 737286:738284 forward [Streptococcus agalactiae SS1, CP010867.1, locus tag RDF_0721]

ATGAAAGTTTTACTGTATTTAGAGGCTGAAGAATACTTGAAAAAGTCAGGTATCGGTCGTGCTATCAAACATCAAGAGAAAGCTCTTCAAATTGCTGGGATAGATTATACCACTAATCCTGCAGATGACTTTGACCTTGTTCATATGAATACCTATGGTATTAGAAGTTGGCTTTTGATGAGTAAAGCTAAAAAAACTGGGAAAAAAGTAATTATGCATGGTCATTCCACTGAAGAGGACTTTAGAAATTCTTTTATTGGCTCAAATTTAGTATCCCCGCTTTTTAAATGGTACCTTTGCCGTTTTTATCAAAAAGCAGATGCCATTATAACCCCAACTGATTATTCTAAACAACTAATAAAAGCATATGGCATCAAGAAACCAATTTTTGTTTTATCTAACGGGATTGATCTAAGTCGCTATCAGAGGTCAGAAAAGAAAGAAGCTGCATTTCGTCACTATTTTCACTTGAGTAAAGATGATAAAGTTGTTATGGGGGCCGGTCTTTATTTCATGCGTAAGGGAATTGACCAATTTGTTGAAGTAGCTGCAAAAATGCCTGATATTCGTTTTATTTGGTTTGGAGAAACAAATAAGTGGGTTATTCCTCGAAAGGTTAGGCAAATCGTTACCAAGCAACATCCCTCAAATGTTACTTTTGCTGGTTACATAAAAGGTGACGTTTATGAAGGTGCCATGAGTGCTAGCGATGCTTTCTTCTTTCCGAGTCGTGAAGAGACAGAAGGGATAGTAGTTTTAGAGGCTTTAGCTAGTCATCAGCATGTGGTTTTAAGAGATATCCCTGTGTATCACGGTTGGGTGACCGAAGACAGTGTTGAATTAGCGACTGATGTTGATGGTTTTGTTGAAAAATTGGATAAAGTCTTATCTGGTAAGAGTGATAAGATTAAAGAAGGTTATCATGTGGCTGAGAGTCGAAGTATCGAACGAATTGCCCATGAATTGGCAAGTGTTTATCAAAAAGTAATGGAGTTGTAA

>8__pbsP1__gbs0428__11 gbs0428 Unknown 450011:451576 forward [Streptococcus agalactiae NEM316, AL732656, locus tag gbs0428]

ATGAAAATATCACAATACAATAAATGGTCAATTCGCCGATTGAAAGTTGGAGCTGCATCTGTAATGATTGCTAGTGGAAGCATTGTTGCTTTAGGGCAATCTCATATTGTTTCAGCAGATGAGATGTCCCAACCTAAAACAACCATTACAGCTCCAACAGCTAACACTTCTACAAATGTAGAAAGTAGTACTGATAAGGCATTATCAAAAGTTACTACGATGGAGACAAGTTCAGAAATGCCTAAAATGCAAAATATGGCAAAAGTTGAAAAAACGAGTGACAAACCTATGATGGTGGCTACCTCTGTTAGAAAAATGATGGCAACGCCGACACCTGTAGCGATGACCAAAACGACTAGTGTAGATGAGGTAAAGAAATCAACTGATACTGCTTTTAAACAGACAGTAGACGTTCCGGCTCATTATGTTAATGCAGCAAAAGGGAATGGCCCATTTCTAGCAGGTGTAAATCAAACGATTCCATATGAAGCGTTTGGTGGAGATGGCATGTTAACGCGTTTAATCCTTAAATCTTCTGAAGGAGCAAAATGGTCAGATAATGGTGTTGATAAAAATAGTCCATTATTACCATTGAAAGGCTTAACTAAAGGTAAATATTTCTATCAAGTTTCTTTAAATGGTAATACGACAGGTAAAGAGGGACAAGCTCTCTTAGATCAAATTAAAGCTAATGATAAACATAGCTACCAAGCCACAATTAGAGTTTATGGTGCTAAAGATGGTAAAGTTGATTTAAAAAACATGATTAGCCAAAAAATGGTAACCATTAACATTCCTCACATTACAACTGATATGGAAGTAAAAAATTCTCTAAAGATGGCCTTTAAAGAAAAAGTAGACGTCCCAGCAAAATATGTTAGTGCTGCAAAAGCTAAAGGTCCATTTTTAGCAGGTGTTAACGAAACGATTCCATATGAAGCGTTTGGTGGGGATGGCATGTTAACGCGTTTAATCCTTAAAGCTTCAGAGGGAGCAAAATGGTCAGACAATGGTGTTGATAAAAATAGTCCATTATTACCGTTAAAAGATTTAACTAAGGGTAAATACTTCTACCAAGTCTCTCTTAATGGCAATACAGCCGGTAAAAAAGGCCAAGCTCTCTTAGATCAAATTAAGGCAAATGGAAGCCATACTTATCAAGCAACTATCACTATTTATGGTACGAAAGATGGCAAAGTTGATATGAATACTATTTTAGGACAAAAGACAGTGATGATTCATATTAATGTCGCTAAAAAAGATATGAATAGCACATCAATGATGATGAAAAAAGATAAGATGACAATGCCTATGAAGAAAGAAATGACTTCATCTAAAATAAATACCGGTATGATGATGTCTAACAACAAGATGTCAGCTAATATGCAAATGTCATCACAAGCCAAATCAAATGATAAAGCTGGTAAAAAAATGTCTATGATGTCTAAAAATTTACCTAATACAGGTGAAACAAAGCAACAAAATGTAGGTGTGCTAGGTATGCTTAGTCTAGCCTTTGCGACAGGTCTAACTGCGCTAGGCCTTAAAAAATCTAAACAAAGATAG

>9__pbsP2__fdaargos512_00736__12 fdaargos512_00736 Cell wall surface anchor family protein,gram-positive signal peptide, YSIRK family,Fibronectin-binding repeat 724001:725116 reverse [Streptococcus agalactiae FDAARGOS_512, CP033822.1, locus tag EGX82_03735]

ATGAAAATATCACAATACAATAAATGGTCAATTCGCCGATTGAAAGTTGGAGCTGCATCTGTAATGATTGCTAGTGGAAGCATTGTTGCTTTAGGGCAATCTCATATTGTTTCAGCAGATGAGATGTCCCAACCTAAAACAACCATTACAGCCCCAACAGCTAACACTTCTACAAATGTAGAAAGTAGTACTGATAAGGCATTATCAAAAGTTACTACGATGGAGACAAGTTCAGAAATGCCTAAAATGCAAAATATGGCAAAAGTTGAAAAAACGAGTGACAAACCTATGATGGTGGCTACCTCTGTTAGAAAAATGATGGCAACGCCGACACCTGTAGCGATGACCAAAACGACTAGTGTAGATGAGGTAAAGAAATCAACTGATACTGCTTTTAAACAGACAGTAGACGTTCCGGCTCATTATGTTAATGCAGCAAAAGGGAATGGCCCATTTCTAGCAGGTGTAAATCAAACGATTCCATATGAAGCGTTTGGTGGAGACGGCATGTTAACGCGTTTAATCCTTAAAGCTTCAGAGGGAGCAAAATGGTCAGACAATGGTGTTGATAAAAATAGTCCATTATTACCGTTAAAAGATTTAACTAAGGGTAAATACTTCTACCAAGTCTCTCTTAATGGCAATACAGCCGGTAAAAAAGGCCAAGCTCTCTTAGATCAAATTAAGGCAAATGGAAGCCATACTTATCAAGCAACTATCACTATTTATGGTACGAAAGATGGCAAAGTTGATATGAATACTATTTTAGGACAAAAGACAGTGATGATTCATATTAATGTCGCTAAAAAAGATATGAATAGCACATCAATGATGATGAAAAAAGATAAGATGACAATGCCTATGAAGAAAGAAATGACTTCATCTAAAATAAATACCGGTATGATGATGTCTAACAACAAGATGTCAGCTAATATGCAAATGTCATCACAAGCCAAATCAAATGATAAAGCTGGTAAAAAAATGTCTATGATGTCTAAAAATTTACCTAATACAGGTGAAACAAAGCAACAAAATGTAGGTGTGCTAGGTATGCTTAGTCTAGCCTTTGCGACAGGTCTAACTGCGCTAGGCCTTAAAAAATCTAAACAAAGATAG

>10__fbsC__SS1_00863__13 SS1_00863 Cell wall surface anchor family protein,Predicted outer membrane protein,Bacterial Ig-like domain (group 1) 801956:803494 forward [Streptococcus agalactiae SS1, CP010867.1, locus tag RDF_0782]

ATGAATAAATCATTCAATACCAAATTAGGTTTGGTAACTGTAGCGGTATTATCAGGTATTGTATTAACGTCTCAATTACCTGTGAACGCTAAAGCTGAGACACCAGTAATGGCAGCAAGTGCACAACAAGGTTTCCGTTTTGTAGCAACCGTTGTAGATAGTCAGACGCATGTCTTGCCGGGAAAATTGGTAACTTTGTCAGAGGTGACAAGTGGTCAGCCTAAAATTATTGCAAGTGTGAAAACGAATGATGCCGGACAAGCTATTTTTAATAATCTCCCTATAAAACGTAACCTTAGTGTGTCTGTTGATGGACAAGTAAAAGGATATACTATTCGAACTGACGTAGCAGGTTCTAGTAAAGCAGCTAGTTTTACAGCAACAGGAGTAGGGACAAATGAGCCAACGTATTCAAAAAAAACCATAGACATCACAGTGAGAGACCAGAATGCTGAACCTGTTTCAGGCAGGACAGTTACCTTAAAAACACAAGCTGGTCGTGAGATTGCTAGCTTGGTTTCTGGTGACAATGGATTAACTCGCTTTACTGATCGTTTACTAGATGGAACATTCTACCAATATTTTGTTGATGGGAAAAAAATTGGTGATATTGTACCTGGAGAATCAAGAAGTGCATATGTTAATGTTGCACCTAAAAAAGATTATTTTACCTTTACAGTCACTGCTTTAGATAAAGATGGACTTGTTGTTAAGGGAAAAGAAGTAACGTTGACTGATATTACTGATGGTAAAGCAGTAGCGCTTGCTTCTTTAAAAACGAATGATAATGGACAAGCTATCTTTACCAACCTACCATTATCAAGAAATATCAGTGTTTCAATTGATGGAAAATCAAAGGGCTATACTCTTCGAACTGATGTAGCAGGCTCTCATAAAGCGGCTGCATTCTATGTAGATGGTAAAGGTACCAAAGCACCTCAATTTAGTGCAGAAGCAGCGGTTGTAACTGTTTACGATGCTAATGGAAATACTATAGCAAACCAAGAAGTGACATTAACTAATTCAAATGGGACAATTGTTGCAAAAGGTTTGACTGAAAAAGATGGCAAAGCAAGGTTCGCCAATAAATTAATGGCAGGAACGTTATACAATATTTTTGTAAATGGTATTGAGATGCCTAAAACTGCCCTTGTTGGTAGTGATGTCAGTGTTTTTCTAACAGATAAACAAATTAAGAAAGAAAAACCTGTTACTCCTGATAAATTGGATAAAGAAATGAACCAAGAGAAGGAAAAGGAGCTCTTAGATACTCCAAAACCAGTACCACCTACTTCGAAACCAACAGCACCAATTAAAAATAAAAAGGACGATTCCCCTAAGAAGTTCACGAAAAGTCAATCTTCTAGTTTGAAGTCAAGTGTTAAGGCTATAGGTGCTAAGCCAAAAGCTATGACAGCCTTAGCTAAAAAATTGCCTAAAACTGGTGATCAAGCCATTAGTATTTTAACTTTGGTTGGTTTTATTGTAACAGGGTTAGCGATTATGTTAGGCTTCCTTAAAAAATCACGATCAAAATAA

**Supplementary File 3 - Sequence data accession numbers**

| **Sanger sample ID** | **Lane** | **Accession** |
| --- | --- | --- |
| 4238STDY6426091 | 20280_5#1 | ERR1624733 |
| 4238STDY6426092 | 20280_5#2 | ERR1624734 |
| 4238STDY6426093 | 20280_5#3 | ERR1624735 |
| 4238STDY6426094 | 20280_5#4 | ERR1624736 |
| 4238STDY6426095 | 20280_5#5 | ERR1624737 |
| 4238STDY6426096 | 20280_5#6 | ERR1624738 |
| 4238STDY6426098 | 20280_5#8 | ERR1624740 |
| 4238STDY6426099 | 20280_5#9 | ERR1624741 |
| 4238STDY6426102 | 20280_5#12 | ERR1624744 |
| 4238STDY6426104 | 20280_5#14 | ERR1624746 |
| 4238STDY6426105 | 20280_5#15 | ERR1624747 |
| 4238STDY6426106 | 20280_5#16 | ERR1624748 |
| 4238STDY6426107 | 20280_5#17 | ERR1624749 |
| 4238STDY6426111 | 20280_5#21 | ERR1624753 |
| 4238STDY6426112 | 20280_5#22 | ERR1624754 |
| 4238STDY6426113 | 20280_5#23 | ERR1624755 |
| 4238STDY6426114 | 20280_5#24 | ERR1624756 |
| 4238STDY6426115 | 20280_5#25 | ERR1624757 |
| 4238STDY6426116 | 20280_5#26 | ERR1624758 |
| 4238STDY6426119 | 20280_5#29 | ERR1624761 |
| 4238STDY6426121 | 20280_5#31 | ERR1624763 |
| 4238STDY6426122 | 20280_5#32 | ERR1624764 |
| 4238STDY6426123 | 20280_5#33 | ERR1624765 |
| 4238STDY6426124 | 20280_5#34 | ERR1624766 |
| 4238STDY6426125 | 20280_5#35 | ERR1624767 |
| 4238STDY6426126 | 20280_5#36 | ERR1624768 |
| 4238STDY6426127 | 20280_5#37 | ERR1624769 |
| 4238STDY6426128 | 20280_5#38 | ERR1624770 |
| 4238STDY6426129 | 20280_5#39 | ERR1624771 |
| 4238STDY6426130 | 20280_5#40 | ERR1624772 |
| 4238STDY6426132 | 20280_5#42 | ERR1624774 |
| 4238STDY6426133 | 20280_5#43 | ERR1624775 |
| 4238STDY6426134 | 20280_5#44 | ERR1624776 |
| 4238STDY6426136 | 20280_5#46 | ERR1624778 |
| 4238STDY6426137 | 20280_5#47 | ERR1624779 |
| 4238STDY6426138 | 20280_5#48 | ERR1624780 |
| 4238STDY6426139 | 20280_5#49 | ERR1624781 |
| 4238STDY6426140 | 20280_5#50 | ERR1624782 |
| 4238STDY6426141 | 20280_5#51 | ERR1624783 |
| 4238STDY6426142 | 20280_5#52 | ERR1624784 |
| 4238STDY6426143 | 20280_5#53 | ERR1624785 |
| 4238STDY6426144 | 20280_5#54 | ERR1624786 |
| 4238STDY6426147 | 20280_5#57 | ERR1624789 |
| 4238STDY6426148 | 20280_5#58 | ERR1624790 |
| 4238STDY6426149 | 20280_5#59 | ERR1624791 |
| 4238STDY6426150 | 20280_5#60 | ERR1624792 |
| 4238STDY6426151 | 20280_5#61 | ERR1624793 |
| 4238STDY6426152 | 20280_5#62 | ERR1624794 |
| 4238STDY6426153 | 20280_5#63 | ERR1624795 |
| 4238STDY6426155 | 20280_5#65 | ERR1624797 |
| 4238STDY6426158 | 20280_5#68 | ERR1624800 |
| 4238STDY6426160 | 20280_5#70 | ERR1624802 |
| 4238STDY6426161 | 20280_5#71 | ERR1624803 |
| 4238STDY6426162 | 20280_5#72 | ERR1624804 |
| 4238STDY6426163 | 20280_5#73 | ERR1624805 |
| 4238STDY6426164 | 20280_5#74 | ERR1624806 |
| 4238STDY6426165 | 20280_5#75 | ERR1624807 |
| 4238STDY6426166 | 20280_5#76 | ERR1624808 |
| 4238STDY6426168 | 20280_5#78 | ERR1624810 |
| 4238STDY6426169 | 20280_5#79 | ERR1624811 |
| 4238STDY6426170 | 20280_5#80 | ERR1624812 |
| 4238STDY6426171 | 20280_5#81 | ERR1624813 |
| 4238STDY6426173 | 20280_5#83 | ERR1624815 |
| 4238STDY6426175 | 20280_5#85 | ERR1624817 |
| 4238STDY6426177 | 20280_5#87 | ERR1624819 |
| 4238STDY6426178 | 20280_5#88 | ERR1624820 |
| 4238STDY6426187 | 20280_5#96 | ERR1624828 |
| 4238STDY6426188 | 20280_5#97 | ERR1624829 |
| 4238STDY6426190 | 20280_5#99 | ERR1624831 |
| 4238STDY6426191 | 20280_5#100 | ERR1624832 |
| 4238STDY6426192 | 20280_5#101 | ERR1624833 |
| 4238STDY6426193 | 20280_5#102 | ERR1624834 |
| 4238STDY6426194 | 20280_5#103 | ERR1624835 |
| 4238STDY6426197 | 20280_5#106 | ERR1624838 |
| 4238STDY6426198 | 20280_5#107 | ERR1624839 |
| 4238STDY6426199 | 20280_5#108 | ERR1624840 |
| 4238STDY6426201 | 20280_5#110 | ERR1624842 |
| 4238STDY6426203 | 20280_5#112 | ERR1624844 |
| 4238STDY6426205 | 20280_5#114 | ERR1624846 |
| 4238STDY6426206 | 20280_5#115 | ERR1624847 |
| 4238STDY6426207 | 20280_5#116 | ERR1624848 |
| 4238STDY6426208 | 20280_5#117 | ERR1624849 |
| 4238STDY6426209 | 20280_5#118 | ERR1624850 |
| 4238STDY6426210 | 20280_5#119 | ERR1624851 |
| 4238STDY6426211 | 20280_5#120 | ERR1624852 |
| 4238STDY6426212 | 20280_5#121 | ERR1624853 |
| 4238STDY6426213 | 20280_5#122 | ERR1624854 |
| 4238STDY6426214 | 20280_5#123 | ERR1624855 |
| 4238STDY6426215 | 20280_5#124 | ERR1624856 |
| 4238STDY6426216 | 20280_5#125 | ERR1624857 |
| 4238STDY6426218 | 20280_5#127 | ERR1624859 |
| 4238STDY6426219 | 20280_5#128 | ERR1624860 |
| 4238STDY6426220 | 20280_5#129 | ERR1624861 |
| 4238STDY6426221 | 20280_5#130 | ERR1624862 |
| 4238STDY6426222 | 20280_5#131 | ERR1624863 |
| 4238STDY6426223 | 20280_5#132 | ERR1624864 |
| 4238STDY6426224 | 20280_5#133 | ERR1624865 |
| 4238STDY6426226 | 20280_5#135 | ERR1624867 |
| 4238STDY6426227 | 20280_5#136 | ERR1624868 |
| 4238STDY6426228 | 20280_5#137 | ERR1624869 |
| 4238STDY6426229 | 20280_5#138 | ERR1624870 |
| 4238STDY6426230 | 20280_5#139 | ERR1624871 |
| 4238STDY6426232 | 20280_5#141 | ERR1624873 |
| 4238STDY6426233 | 20280_5#142 | ERR1624874 |
| 4238STDY6426234 | 20280_5#143 | ERR1624875 |
| 4238STDY6426235 | 20280_5#144 | ERR1624876 |
| 4238STDY6426236 | 20280_5#145 | ERR1624877 |
| 4238STDY6426237 | 20280_5#146 | ERR1624878 |
| 4238STDY6426238 | 20280_5#147 | ERR1624879 |
| 4238STDY6426239 | 20280_5#148 | ERR1624880 |
| 4238STDY6426240 | 20280_5#149 | ERR1624881 |
| 4238STDY6426241 | 20280_5#150 | ERR1624882 |
| 4238STDY6426242 | 20280_5#151 | ERR1624883 |
| 4238STDY6426243 | 20280_5#152 | ERR1624884 |
| 4238STDY6426244 | 20280_5#153 | ERR1624885 |
| 4238STDY6426246 | 20280_5#155 | ERR1624887 |
| 4238STDY6426247 | 20280_5#156 | ERR1624888 |
| 4238STDY6426248 | 20280_5#157 | ERR1624889 |
| 4238STDY6426249 | 20280_5#158 | ERR1624890 |
| 4238STDY6426251 | 20280_5#159 | ERR1624891 |
| 4238STDY6426253 | 20280_5#161 | ERR1624893 |
| 4238STDY6426254 | 20280_5#162 | ERR1624894 |
| 4238STDY6426255 | 20280_5#163 | ERR1624895 |
| 4238STDY6426256 | 20280_5#164 | ERR1624896 |
| 4238STDY6426257 | 20280_5#165 | ERR1624897 |
| 4238STDY6426258 | 20280_5#166 | ERR1624898 |
| 4238STDY6426262 | 20280_5#170 | ERR1624902 |
| 4238STDY6426263 | 20280_5#171 | ERR1624903 |
| 4238STDY6426264 | 20280_5#172 | ERR1624904 |
| 4238STDY6426265 | 20280_5#173 | ERR1624905 |
| 4238STDY6426267 | 20280_5#175 | ERR1624907 |
| 4238STDY6426268 | 20280_5#176 | ERR1624908 |
| 4238STDY6426269 | 20280_5#177 | ERR1624909 |
| 4238STDY6426270 | 20280_5#178 | ERR1624910 |
| 4238STDY6426271 | 20280_5#179 | ERR1624911 |
| 4238STDY6426272 | 20280_5#180 | ERR1624912 |
| 4238STDY6426273 | 20280_5#181 | ERR1624913 |
| 4238STDY6426274 | 20280_5#182 | ERR1624914 |
| 4238STDY6426283 | 20280_5#190 | ERR1624922 |
| 4238STDY6426284 | 20280_6#1 | ERR1624923 |
| 4238STDY6426285 | 20280_6#2 | ERR1624924 |
| 4238STDY6426286 | 20280_6#3 | ERR1624925 |
| 4238STDY6426287 | 20280_6#4 | ERR1624926 |
| 4238STDY6426289 | 20280_6#6 | ERR1624928 |
| 4238STDY6426290 | 20280_6#7 | ERR1624929 |
| 4238STDY6426291 | 20280_6#8 | ERR1624930 |
| 4238STDY6426292 | 20280_6#9 | ERR1624931 |
| 4238STDY6426293 | 20280_6#10 | ERR1624932 |
| 4238STDY6426294 | 20280_6#11 | ERR1624933 |
| 4238STDY6426295 | 20280_6#12 | ERR1624934 |
| 4238STDY6426296 | 20280_6#13 | ERR1624935 |
| 4238STDY6426297 | 20280_6#14 | ERR1624936 |
| 4238STDY6426298 | 20280_6#15 | ERR1624937 |
| 4238STDY6426299 | 20280_6#16 | ERR1624938 |
| 4238STDY6426300 | 20280_6#17 | ERR1624939 |
| 4238STDY6426301 | 20280_6#18 | ERR1624940 |
| 4238STDY6426302 | 20280_6#19 | ERR1624941 |
| 4238STDY6426303 | 20280_6#20 | ERR1624942 |
| 4238STDY6426304 | 20280_6#21 | ERR1624943 |
| 4238STDY6426305 | 20280_6#22 | ERR1624944 |
| 4238STDY6426306 | 20280_6#23 | ERR1624945 |
| 4238STDY6426307 | 20280_6#24 | ERR1624946 |
| 4238STDY6426308 | 20280_6#25 | ERR1624947 |
| 4238STDY6426309 | 20280_6#26 | ERR1624948 |
| 4238STDY6426310 | 20280_6#27 | ERR1624949 |
| 4238STDY6426311 | 20280_6#28 | ERR1624950 |
| 4238STDY6426313 | 20280_6#30 | ERR1624952 |
| 4238STDY6426315 | 20280_6#32 | ERR1624954 |
| 4238STDY6426318 | 20280_6#35 | ERR1624957 |
| 4238STDY6426320 | 20280_6#37 | ERR1624959 |
| 4238STDY6426321 | 20280_6#38 | ERR1624960 |
| 4238STDY6426322 | 20280_6#39 | ERR1624961 |
| 4238STDY6426323 | 20280_6#40 | ERR1624962 |
| 4238STDY6426324 | 20280_6#41 | ERR1624963 |
| 4238STDY6426325 | 20280_6#42 | ERR1624964 |
| 4238STDY6426326 | 20280_6#43 | ERR1624965 |
| 4238STDY6426327 | 20280_6#44 | ERR1624966 |
| 4238STDY6426328 | 20280_6#45 | ERR1624967 |
| 4238STDY6426329 | 20280_6#46 | ERR1624968 |
| 4238STDY6426330 | 20280_6#47 | ERR1624969 |
| 4238STDY6426331 | 20280_6#48 | ERR1624970 |
| 4238STDY6426332 | 20280_6#49 | ERR1624971 |
| 4238STDY6426333 | 20280_6#50 | ERR1624972 |
| 4238STDY6426334 | 20280_6#51 | ERR1624973 |
| 4238STDY6426335 | 20280_6#52 | ERR1624974 |
| 4238STDY6426337 | 20280_6#54 | ERR1624976 |
| 4238STDY6426339 | 20280_6#56 | ERR1624978 |
| 4238STDY6426340 | 20280_6#57 | ERR1624979 |
| 4238STDY6426341 | 20280_6#58 | ERR1624980 |
| 4238STDY6426342 | 20280_6#59 | ERR1624981 |
| 4238STDY6426343 | 20280_6#60 | ERR1624982 |
| 4238STDY6426344 | 20280_6#61 | ERR1624983 |
| 4238STDY6426345 | 20280_6#62 | ERR1624984 |
| 4238STDY6426346 | 20280_6#63 | ERR1624985 |
| 4238STDY6426347 | 20280_6#64 | ERR1624986 |
| 4238STDY6426348 | 20280_6#65 | ERR1624987 |
| 4238STDY6426349 | 20280_6#66 | ERR1624988 |
| 4238STDY6426350 | 20280_6#67 | ERR1624989 |
| 4238STDY6426351 | 20280_6#68 | ERR1624990 |
| 4238STDY6426352 | 20280_6#69 | ERR1624991 |
| 4238STDY6426353 | 20280_6#70 | ERR1624992 |
| 4238STDY6426354 | 20280_6#71 | ERR1624993 |
| 4238STDY6426355 | 20280_6#72 | ERR1624994 |
| 4238STDY6426356 | 20280_6#73 | ERR1624995 |
| 4238STDY6426357 | 20280_6#74 | ERR1624996 |
| 4238STDY6426358 | 20280_6#75 | ERR1624997 |
| 4238STDY6426359 | 20280_6#76 | ERR1624998 |
| 4238STDY6426360 | 20280_6#77 | ERR1624999 |
| 4238STDY6426361 | 20280_6#78 | ERR1625000 |
| 4238STDY6426362 | 20280_6#79 | ERR1625001 |
| 4238STDY6426363 | 20280_6#80 | ERR1625002 |
| 4238STDY6426364 | 20280_6#81 | ERR1625003 |
| 4238STDY6426366 | 20280_6#83 | ERR1625005 |
| 4238STDY6426367 | 20280_6#84 | ERR1625006 |
| 4238STDY6426368 | 20280_6#85 | ERR1625007 |
| 4238STDY6426369 | 20280_6#86 | ERR1625008 |
| 4238STDY6426370 | 20280_6#87 | ERR1625009 |
| 4238STDY6426375 | 20280_6#92 | ERR1625014 |
| 4238STDY6426376 | 20280_6#93 | ERR1625015 |
| 4238STDY6426377 | 20280_6#94 | ERR1625016 |
| 4238STDY6426379 | 20280_6#95 | ERR1625017 |
| 4238STDY6426380 | 20280_6#96 | ERR1625018 |
| 4238STDY6426381 | 20280_6#97 | ERR1625019 |
| 4238STDY6426382 | 20280_6#98 | ERR1625020 |
| 4238STDY6426383 | 20280_6#99 | ERR1625021 |
| 4238STDY6426384 | 20280_6#100 | ERR1625022 |
| 4238STDY6426385 | 20280_6#101 | ERR1625023 |
| 4238STDY6426386 | 20280_6#102 | ERR1625024 |
| 4238STDY6426387 | 20280_6#103 | ERR1625025 |
| 4238STDY6426388 | 20280_6#104 | ERR1625026 |
| 4238STDY6426389 | 20280_6#105 | ERR1625027 |
| 4238STDY6426390 | 20280_6#106 | ERR1625028 |
| 4238STDY6426391 | 20280_6#107 | ERR1625029 |
| 4238STDY6426392 | 20280_6#108 | ERR1625030 |
| 4238STDY6426393 | 20280_6#109 | ERR1625031 |
| 4238STDY6426394 | 20280_6#110 | ERR1625032 |
| 4238STDY6426395 | 20280_6#111 | ERR1625033 |
| 4238STDY6426396 | 20280_6#112 | ERR1625034 |
| 4238STDY6426398 | 20280_6#114 | ERR1625036 |
| 4238STDY6426399 | 20280_6#115 | ERR1625037 |
| 4238STDY6426401 | 20280_6#117 | ERR1625039 |
| 4238STDY6426402 | 20280_6#118 | ERR1625040 |
| 4238STDY6426403 | 20280_6#119 | ERR1625041 |
| 4238STDY6426404 | 20280_6#120 | ERR1625042 |
| 4238STDY6426405 | 20280_6#121 | ERR1625043 |
| 4238STDY6426406 | 20280_6#122 | ERR1625044 |
| 4238STDY6426407 | 20280_6#123 | ERR1625045 |
| 4238STDY6426409 | 20280_6#125 | ERR1625047 |
| 4238STDY6426410 | 20280_6#126 | ERR1625048 |
| 4238STDY6426411 | 20280_6#127 | ERR1625049 |
| 4238STDY6426412 | 20280_6#128 | ERR1625050 |
| 4238STDY6426413 | 20280_6#129 | ERR1625051 |
| 4238STDY6426414 | 20280_6#130 | ERR1625052 |
| 4238STDY6426415 | 20280_6#131 | ERR1625053 |
| 4238STDY6426416 | 20280_6#132 | ERR1625054 |
| 4238STDY6426417 | 20280_6#133 | ERR1625055 |
| 4238STDY6426418 | 20280_6#134 | ERR1625056 |
| 4238STDY6426419 | 20280_6#135 | ERR1625057 |
| 4238STDY6426420 | 20280_6#136 | ERR1625058 |
| 4238STDY6426421 | 20280_6#137 | ERR1625059 |
| 4238STDY6426422 | 20280_6#138 | ERR1625060 |
| 4238STDY6426423 | 20280_6#139 | ERR1625061 |
| 4238STDY6426424 | 20280_6#140 | ERR1625062 |
| 4238STDY6426425 | 20280_6#141 | ERR1625063 |
| 4238STDY6426426 | 20280_6#142 | ERR1625064 |
| 4238STDY6426428 | 20280_6#144 | ERR1625066 |
| 4238STDY6426429 | 20280_6#145 | ERR1625067 |
| 4238STDY6426430 | 20280_6#146 | ERR1625068 |
| 4238STDY6426431 | 20280_6#147 | ERR1625069 |
| 4238STDY6426432 | 20280_6#148 | ERR1625070 |
| 4238STDY6426433 | 20280_6#149 | ERR1625071 |
| 4238STDY6426434 | 20280_6#150 | ERR1625072 |
| 4238STDY6426437 | 20280_6#153 | ERR1625075 |
| 4238STDY6426438 | 20280_6#154 | ERR1625076 |
| 4238STDY6426439 | 20280_6#155 | ERR1625077 |
| 4238STDY6426440 | 20280_6#156 | ERR1625078 |
| 4238STDY6426441 | 20280_6#157 | ERR1625079 |
| 4238STDY6426442 | 20280_6#158 | ERR1625080 |
| 4238STDY6426444 | 20280_6#160 | ERR1625082 |
| 4238STDY6426445 | 20280_6#161 | ERR1625083 |
| 4238STDY6426446 | 20280_6#162 | ERR1625084 |
| 4238STDY6426447 | 20280_6#163 | ERR1625085 |
| 4238STDY6426448 | 20280_6#164 | ERR1625086 |
| 4238STDY6426451 | 20280_6#167 | ERR1625089 |
| 4238STDY6426452 | 20280_6#168 | ERR1625090 |
| 4238STDY6426453 | 20280_6#169 | ERR1625091 |
| 4238STDY6426454 | 20280_6#170 | ERR1625092 |
| 4238STDY6426455 | 20280_6#171 | ERR1625093 |
| 4238STDY6426456 | 20280_6#172 | ERR1625094 |
| 4238STDY6426457 | 20280_6#173 | ERR1625095 |
| 4238STDY6426458 | 20280_6#174 | ERR1625096 |
| 4238STDY6426459 | 20280_6#175 | ERR1625097 |
| 4238STDY6426460 | 20280_6#176 | ERR1625098 |
| 4238STDY6426461 | 20280_6#177 | ERR1625099 |
| 4238STDY6426462 | 20280_6#178 | ERR1625100 |
| 4238STDY6426463 | 20280_6#179 | ERR1625101 |
| 4238STDY6426464 | 20280_6#180 | ERR1625102 |
| 4238STDY6426465 | 20280_6#181 | ERR1625103 |
| 4238STDY6426467 | 20280_6#183 | ERR1625105 |
| 4238STDY6426468 | 20280_6#184 | ERR1625106 |
| 4238STDY6426469 | 20280_6#185 | ERR1625107 |
| 4238STDY6426470 | 20280_6#186 | ERR1625108 |
| 4238STDY6426471 | 20280_6#187 | ERR1625109 |
| 4238STDY6426472 | 20280_6#188 | ERR1625110 |
| 4238STDY6426473 | 20280_6#189 | ERR1625111 |
| 4238STDY6426475 | 20280_6#190 | ERR1625112 |
| 4238STDY6426476 | 20280_7#1 | ERR1625113 |
| 4238STDY6426477 | 20280_7#2 | ERR1625114 |
| 4238STDY6426478 | 20280_7#3 | ERR1625115 |
| 4238STDY6426479 | 20280_7#4 | ERR1625116 |
| 4238STDY6426480 | 20280_7#5 | ERR1625117 |
| 4238STDY6426481 | 20280_7#6 | ERR1625118 |
| 4238STDY6426482 | 20280_7#7 | ERR1625119 |
| 4238STDY6426483 | 20280_7#8 | ERR1625120 |
| 4238STDY6426484 | 20280_7#9 | ERR1625121 |
| 4238STDY6426485 | 20280_7#10 | ERR1625122 |
| 4238STDY6426486 | 20280_7#11 | ERR1625123 |
| 4238STDY6426487 | 20280_7#12 | ERR1625124 |
| 4238STDY6426488 | 20280_7#13 | ERR1625125 |
| 4238STDY6426489 | 20280_7#14 | ERR1625126 |
| 4238STDY6426490 | 20280_7#15 | ERR1625127 |
| 4238STDY6426491 | 20280_7#16 | ERR1625128 |
| 4238STDY6426492 | 20280_7#17 | ERR1625129 |
| 4238STDY6426493 | 20280_7#18 | ERR1625130 |
| 4238STDY6426494 | 20280_7#19 | ERR1625131 |
| 4238STDY6426495 | 20280_7#20 | ERR1625132 |
| 4238STDY6426496 | 20280_7#21 | ERR1625133 |
| 4238STDY6426497 | 20280_7#22 | ERR1625134 |
| 4238STDY6426498 | 20280_7#23 | ERR1625135 |
| 4238STDY6426499 | 20280_7#24 | ERR1625136 |
| 4238STDY6426500 | 20280_7#25 | ERR1625137 |
| 4238STDY6426501 | 20280_7#26 | ERR1625138 |
| 4238STDY6426502 | 20280_7#27 | ERR1625139 |
| 4238STDY6426504 | 20280_7#29 | ERR1625141 |
| 4238STDY6426505 | 20280_7#30 | ERR1625142 |
| 4238STDY6426506 | 20280_7#31 | ERR1625143 |
| 4238STDY6426507 | 20280_7#32 | ERR1625144 |
| 4238STDY6426508 | 20280_7#33 | ERR1625145 |
| 4238STDY6426509 | 20280_7#34 | ERR1625146 |
| 4238STDY6426510 | 20280_7#35 | ERR1625147 |
| 4238STDY6426511 | 20280_7#36 | ERR1625148 |
| 4238STDY6426512 | 20280_7#37 | ERR1625149 |
| 4238STDY6426513 | 20280_7#38 | ERR1625150 |
| 4238STDY6426514 | 20280_7#39 | ERR1625151 |
| 4238STDY6426515 | 20280_7#40 | ERR1625152 |
| 4238STDY6426516 | 20280_7#41 | ERR1625153 |
| 4238STDY6426517 | 20280_7#42 | ERR1625154 |
| 4238STDY6426518 | 20280_7#43 | ERR1625155 |
| 4238STDY6426519 | 20280_7#44 | ERR1625156 |
| 4238STDY6426520 | 20280_7#45 | ERR1625157 |
| 4238STDY6426521 | 20280_7#46 | ERR1625158 |
| 4238STDY6426522 | 20280_7#47 | ERR1625159 |
| 4238STDY6426523 | 20280_7#48 | ERR1625160 |
| 4238STDY6426524 | 20280_7#49 | ERR1625161 |
| 4238STDY6426525 | 20280_7#50 | ERR1625162 |
| 4238STDY6426526 | 20280_7#51 | ERR1625163 |
| 4238STDY6426527 | 20280_7#52 | ERR1625164 |
| 4238STDY6426528 | 20280_7#53 | ERR1625165 |
| 4238STDY6426529 | 20280_7#54 | ERR1625166 |
| 4238STDY6426530 | 20280_7#55 | ERR1625167 |
| 4238STDY6426531 | 20280_7#56 | ERR1625168 |
| 4238STDY6426532 | 20280_7#57 | ERR1625169 |
| 4238STDY6426533 | 20280_7#58 | ERR1625170 |
| 4238STDY6426535 | 20280_7#60 | ERR1625172 |
| 4238STDY6426536 | 20280_7#61 | ERR1625173 |
| 4238STDY6426537 | 20280_7#62 | ERR1625174 |
| 4238STDY6426539 | 20280_7#64 | ERR1625176 |
| 4238STDY6426541 | 20280_7#66 | ERR1625178 |
| 4238STDY6426542 | 20280_7#67 | ERR1625179 |
| 4238STDY6426543 | 20280_7#68 | ERR1625180 |
| 4238STDY6426544 | 20280_7#69 | ERR1625181 |
| 4238STDY6426545 | 20280_7#70 | ERR1625182 |
| 4238STDY6426546 | 20280_7#71 | ERR1625183 |
| 4238STDY6426547 | 20280_7#72 | ERR1625184 |
| 4238STDY6426548 | 20280_7#73 | ERR1625185 |
| 4238STDY6426549 | 20280_7#74 | ERR1625186 |
| 4238STDY6426550 | 20280_7#75 | ERR1625187 |
| 4238STDY6426551 | 20280_7#76 | ERR1625188 |
| 4238STDY6426552 | 20280_7#77 | ERR1625189 |
| 4238STDY6426553 | 20280_7#78 | ERR1625190 |
| 4238STDY6426554 | 20280_7#79 | ERR1625191 |
| 4238STDY6426555 | 20280_7#80 | ERR1625192 |
| 4238STDY6426556 | 20280_7#81 | ERR1625193 |
| 4238STDY6426557 | 20280_7#82 | ERR1625194 |
| 4238STDY6426558 | 20280_7#83 | ERR1625195 |
| 4238STDY6426559 | 20280_7#84 | ERR1625196 |
| 4238STDY6426560 | 20280_7#85 | ERR1625197 |
| 4238STDY6426561 | 20280_7#86 | ERR1625198 |
| 4238STDY6426562 | 20280_7#87 | ERR1625199 |
| 4238STDY6426563 | 20280_7#88 | ERR1625200 |
| 4238STDY6426564 | 20280_7#89 | ERR1625201 |
| 4238STDY6426565 | 20280_7#90 | ERR1625202 |
| 4238STDY6426568 | 20280_7#93 | ERR1625205 |
| 4238STDY6426569 | 20280_7#94 | ERR1625206 |
| 4238STDY6426571 | 20280_7#95 | ERR1625207 |
| 4238STDY6426572 | 20280_7#96 | ERR1625208 |
| 4238STDY6426573 | 20280_7#97 | ERR1625209 |
| 4238STDY6426574 | 20280_7#98 | ERR1625210 |
| 4238STDY6426575 | 20280_7#99 | ERR1625211 |
| 4238STDY6426576 | 20280_7#100 | ERR1625212 |
| 4238STDY6426577 | 20280_7#101 | ERR1625213 |
| 4238STDY6426578 | 20280_7#102 | ERR1625214 |
| 4238STDY6426579 | 20280_7#103 | ERR1625215 |
| 4238STDY6426580 | 20280_7#104 | ERR1625216 |
| 4238STDY6426581 | 20280_7#105 | ERR1625217 |
| 4238STDY6426582 | 20280_7#106 | ERR1625218 |
| 4238STDY6426583 | 20280_7#107 | ERR1625219 |
| 4238STDY6426585 | 20280_7#109 | ERR1625221 |
| 4238STDY6426586 | 20280_7#110 | ERR1625222 |
| 4238STDY6426587 | 20280_7#111 | ERR1625223 |
| 4238STDY6426588 | 20280_7#112 | ERR1625224 |
| 4238STDY6426590 | 20280_7#114 | ERR1625226 |
| 4238STDY6426591 | 20280_7#115 | ERR1625227 |
| 4238STDY6426592 | 20280_7#116 | ERR1625228 |
| 4238STDY6426593 | 20280_7#117 | ERR1625229 |
| 4238STDY6426594 | 20280_7#118 | ERR1625230 |
| 4238STDY6426595 | 20280_7#119 | ERR1625231 |
| 4238STDY6426596 | 20280_7#120 | ERR1625232 |
| 4238STDY6426597 | 20280_7#121 | ERR1625233 |
| 4238STDY6426598 | 20280_7#122 | ERR1625234 |
| 4238STDY6426599 | 20280_7#123 | ERR1625235 |
| 4238STDY6426600 | 20280_7#124 | ERR1625236 |
| 4238STDY6426601 | 20280_7#125 | ERR1625237 |
| 4238STDY6426603 | 20280_7#127 | ERR1625239 |
| 4238STDY6426604 | 20280_7#128 | ERR1625240 |
| 4238STDY6426605 | 20280_7#129 | ERR1625241 |
| 4238STDY6426606 | 20280_7#130 | ERR1625242 |
| 4238STDY6426607 | 20280_7#131 | ERR1625243 |
| 4238STDY6426608 | 20280_7#132 | ERR1625244 |
| 4238STDY6426609 | 20280_7#133 | ERR1625245 |
| 4238STDY6426610 | 20280_7#134 | ERR1625246 |
| 4238STDY6426611 | 20280_7#135 | ERR1625247 |
| 4238STDY6426612 | 20280_7#136 | ERR1625248 |
| 4238STDY6426613 | 20280_7#137 | ERR1625249 |
| 4238STDY6426614 | 20280_7#138 | ERR1625250 |
| 4238STDY6426615 | 20280_7#139 | ERR1625251 |
| 4238STDY6426616 | 20280_7#140 | ERR1625252 |
| 4238STDY6426617 | 20280_7#141 | ERR1625253 |
| 4238STDY6426618 | 20280_7#142 | ERR1625254 |
| 4238STDY6426619 | 20280_7#143 | ERR1625255 |
| 4238STDY6426620 | 20280_7#144 | ERR1625256 |
| 4238STDY6426621 | 20280_7#145 | ERR1625257 |
| 4238STDY6426622 | 20280_7#146 | ERR1625258 |
| 4238STDY6426623 | 20280_7#147 | ERR1625259 |
| 4238STDY6426624 | 20280_7#148 | ERR1625260 |
| 4238STDY6426626 | 20280_7#150 | ERR1625262 |
| 4238STDY6426627 | 20280_7#151 | ERR1625263 |
| 4238STDY6426628 | 20280_7#152 | ERR1625264 |
| 4238STDY6426629 | 20280_7#153 | ERR1625265 |
| 4238STDY6426631 | 20280_7#155 | ERR1625267 |
| 4238STDY6426633 | 20280_7#157 | ERR1625269 |
| 4238STDY6426634 | 20280_7#158 | ERR1625270 |
| 4238STDY6426635 | 20280_7#159 | ERR1625271 |
| 4238STDY6426636 | 20280_7#160 | ERR1625272 |
| 4238STDY6426637 | 20280_7#161 | ERR1625273 |
| 4238STDY6426638 | 20280_7#162 | ERR1625274 |
| 4238STDY6426639 | 20280_7#163 | ERR1625275 |
| 4238STDY6426640 | 20280_7#164 | ERR1625276 |
| 4238STDY6426641 | 20280_7#165 | ERR1625277 |
| 4238STDY6426642 | 20280_7#166 | ERR1625278 |
| 4238STDY6426643 | 20280_7#167 | ERR1625279 |
| 4238STDY6426645 | 20280_7#169 | ERR1625281 |
| 4238STDY6426646 | 20280_7#170 | ERR1625282 |
| 4238STDY6426647 | 20280_7#171 | ERR1625283 |
| 4238STDY6426648 | 20280_7#172 | ERR1625284 |
| 4238STDY6426649 | 20280_7#173 | ERR1625285 |
| 4238STDY6426650 | 20280_7#174 | ERR1625286 |
| 4238STDY6426651 | 20280_7#175 | ERR1625287 |
| 4238STDY6426652 | 20280_7#176 | ERR1625288 |
| 4238STDY6426653 | 20280_7#177 | ERR1625289 |
| 4238STDY6426654 | 20280_7#178 | ERR1625290 |
| 4238STDY6426655 | 20280_7#179 | ERR1625291 |
| 4238STDY6426656 | 20280_7#180 | ERR1625292 |
| 4238STDY6426657 | 20280_7#181 | ERR1625293 |
| 4238STDY6426658 | 20280_7#182 | ERR1625294 |
| 4238STDY6426659 | 20280_7#183 | ERR1625295 |
| 4238STDY6426663 | 20280_7#187 | ERR1625299 |
| 4238STDY6426664 | 20280_7#188 | ERR1625300 |
| 4238STDY6426665 | 20280_7#189 | ERR1625301 |
| 4238STDY6426667 | 20280_7#190 | ERR1625302 |
| 4238STDY6426668 | 20280_8#1 | ERR1625303 |
| 4238STDY6426669 | 20280_8#2 | ERR1625304 |
| 4238STDY6426670 | 20280_8#3 | ERR1625305 |
| 4238STDY6426671 | 20280_8#4 | ERR1625306 |
| 4238STDY6426672 | 20280_8#5 | ERR1625307 |
| 4238STDY6426673 | 20280_8#6 | ERR1625308 |
| 4238STDY6426674 | 20280_8#7 | ERR1625309 |
| 4238STDY6426675 | 20280_8#8 | ERR1625310 |
| 4238STDY6426676 | 20280_8#9 | ERR1625311 |
| 4238STDY6426677 | 20280_8#10 | ERR1625312 |
| 4238STDY6426678 | 20280_8#11 | ERR1625313 |
| 4238STDY6426679 | 20280_8#12 | ERR1625314 |
| 4238STDY6426682 | 20280_8#15 | ERR1625317 |
| 4238STDY6426683 | 20280_8#16 | ERR1625318 |
| 4238STDY6426684 | 20280_8#17 | ERR1625319 |
| 4238STDY6426685 | 20280_8#18 | ERR1625320 |
| 4238STDY6426686 | 20280_8#19 | ERR1625321 |
| 4238STDY6426687 | 20280_8#20 | ERR1625322 |
| 4238STDY6426688 | 20280_8#21 | ERR1625323 |
| 4238STDY6426689 | 20280_8#22 | ERR1625324 |
| 4238STDY6426690 | 20280_8#23 | ERR1625325 |
| 4238STDY6426691 | 20280_8#24 | ERR1625326 |
| 4238STDY6426692 | 20280_8#25 | ERR1625327 |
| 4238STDY6426693 | 20280_8#26 | ERR1625328 |
| 4238STDY6426694 | 20280_8#27 | ERR1625329 |
| 4238STDY6426695 | 20280_8#28 | ERR1625330 |
| 4238STDY6426696 | 20280_8#29 | ERR1625331 |
| 4238STDY6426698 | 20280_8#31 | ERR1625333 |
| 4238STDY6426699 | 20280_8#32 | ERR1625334 |
| 4238STDY6426700 | 20280_8#33 | ERR1625335 |
| 4238STDY6426701 | 20280_8#34 | ERR1625336 |
| 4238STDY6426702 | 20280_8#35 | ERR1625337 |
| 4238STDY6426703 | 20280_8#36 | ERR1625338 |
| 4238STDY6426705 | 20280_8#38 | ERR1625340 |
| 4238STDY6426706 | 20280_8#39 | ERR1625341 |
| 4238STDY6426707 | 20280_8#40 | ERR1625342 |
| 4238STDY6426708 | 20280_8#41 | ERR1625343 |
| 4238STDY6426710 | 20280_8#43 | ERR1625345 |
| 4238STDY6426711 | 20280_8#44 | ERR1625346 |
| 4238STDY6426712 | 20280_8#45 | ERR1625347 |
| 4238STDY6426713 | 20280_8#46 | ERR1625348 |
| 4238STDY6426714 | 20280_8#47 | ERR1625349 |
| 4238STDY6426716 | 20280_8#49 | ERR1625351 |
| 4238STDY6426717 | 20280_8#50 | ERR1625352 |
| 4238STDY6426718 | 20280_8#51 | ERR1625353 |
| 4238STDY6426719 | 20280_8#52 | ERR1625354 |
| 4238STDY6426720 | 20280_8#53 | ERR1625355 |
| 4238STDY6426721 | 20280_8#54 | ERR1625356 |
| 4238STDY6426722 | 20280_8#55 | ERR1625357 |
| 4238STDY6426723 | 20280_8#56 | ERR1625358 |
| 4238STDY6426724 | 20280_8#57 | ERR1625359 |
| 4238STDY6426726 | 20280_8#59 | ERR1625361 |
| 4238STDY6426727 | 20280_8#60 | ERR1625362 |
| 4238STDY6426728 | 20280_8#61 | ERR1625363 |
| 4238STDY6426729 | 20280_8#62 | ERR1625364 |
| 4238STDY6426730 | 20280_8#63 | ERR1625365 |
| 4238STDY6426731 | 20280_8#64 | ERR1625366 |
| 4238STDY6426732 | 20280_8#65 | ERR1625367 |
| 4238STDY6426733 | 20280_8#66 | ERR1625368 |
| 4238STDY6426734 | 20280_8#67 | ERR1625369 |
| 4238STDY6426735 | 20280_8#68 | ERR1625370 |
| 4238STDY6426736 | 20280_8#69 | ERR1625371 |
| 4238STDY6426737 | 20280_8#70 | ERR1625372 |
| 4238STDY6426738 | 20280_8#71 | ERR1625373 |
| 4238STDY6426741 | 20280_8#74 | ERR1625376 |
| 4238STDY6426742 | 20280_8#75 | ERR1625377 |
| 4238STDY6426743 | 20280_8#76 | ERR1625378 |
| 4238STDY6426744 | 20280_8#77 | ERR1625379 |
| 4238STDY6426746 | 20280_8#79 | ERR1625381 |
| 4238STDY6426747 | 20280_8#80 | ERR1625382 |
| 4238STDY6426748 | 20280_8#81 | ERR1625383 |
| 4238STDY6426749 | 20280_8#82 | ERR1625384 |
| 4238STDY6426750 | 20280_8#83 | ERR1625385 |
| 4238STDY6426751 | 20280_8#84 | ERR1625386 |
| 4238STDY6426752 | 20280_8#85 | ERR1625387 |
| 4238STDY6426753 | 20280_8#86 | ERR1625388 |
| 4238STDY6426756 | 20280_8#89 | ERR1625391 |
| 4238STDY6426757 | 20280_8#90 | ERR1625392 |
| 4238STDY6426758 | 20280_8#91 | ERR1625393 |
| 4238STDY6426759 | 20280_8#92 | ERR1625394 |
| 4238STDY6426760 | 20280_8#93 | ERR1625395 |
| 4238STDY6426761 | 20280_8#94 | ERR1625396 |
| 4238STDY6426763 | 20280_8#95 | ERR1625397 |
| 4238STDY6426764 | 20280_8#96 | ERR1625398 |
| 4238STDY6426765 | 20280_8#97 | ERR1625399 |
| 4238STDY6426766 | 20280_8#98 | ERR1625400 |
| 4238STDY6426767 | 20280_8#99 | ERR1625401 |
| 4238STDY6426768 | 20280_8#100 | ERR1625402 |
| 4238STDY6426769 | 20280_8#101 | ERR1625403 |
| 4238STDY6426770 | 20280_8#102 | ERR1625404 |
| 4238STDY6426771 | 20280_8#103 | ERR1625405 |
| 4238STDY6426772 | 20280_8#104 | ERR1625406 |
| 4238STDY6426773 | 20280_8#105 | ERR1625407 |
| 4238STDY6426774 | 20280_8#106 | ERR1625408 |
| 4238STDY6426775 | 20280_8#107 | ERR1625409 |
| 4238STDY6426776 | 20280_8#108 | ERR1625410 |
| 4238STDY6426777 | 20280_8#109 | ERR1625411 |
| 4238STDY6426778 | 20280_8#110 | ERR1625412 |
| 4238STDY6426780 | 20280_8#112 | ERR1625414 |
| 4238STDY6426781 | 20280_8#113 | ERR1625415 |
| 4238STDY6426782 | 20280_8#114 | ERR1625416 |
| 4238STDY6426783 | 20280_8#115 | ERR1625417 |
| 4238STDY6426784 | 20280_8#116 | ERR1625418 |
| 4238STDY6426785 | 20280_8#117 | ERR1625419 |
| 4238STDY6426787 | 20280_8#119 | ERR1625421 |
| 4238STDY6426788 | 20280_8#120 | ERR1625422 |
| 4238STDY6426789 | 20280_8#121 | ERR1625423 |
| 4238STDY6426790 | 20280_8#122 | ERR1625424 |
| 4238STDY6426791 | 20280_8#123 | ERR1625425 |
| 4238STDY6426792 | 20280_8#124 | ERR1625426 |
| 4238STDY6426793 | 20280_8#125 | ERR1625427 |
| 4238STDY6426794 | 20280_8#126 | ERR1625428 |
| 4238STDY6426795 | 20280_8#127 | ERR1625429 |
| 4238STDY6426797 | 20280_8#129 | ERR1625431 |
| 4238STDY6426798 | 20280_8#130 | ERR1625432 |
| 4238STDY6426799 | 20280_8#131 | ERR1625433 |
| 4238STDY6426801 | 20280_8#133 | ERR1625435 |
| 4238STDY6426802 | 20280_8#134 | ERR1625436 |
| 4238STDY6426804 | 20280_8#136 | ERR1625438 |
| 4238STDY6426805 | 20280_8#137 | ERR1625439 |
| 4238STDY6426806 | 20280_8#138 | ERR1625440 |
| 4238STDY6426807 | 20280_8#139 | ERR1625441 |
| 4238STDY6426808 | 20280_8#140 | ERR1625442 |
| 4238STDY6426809 | 20280_8#141 | ERR1625443 |
| 4238STDY6426810 | 20280_8#142 | ERR1625444 |
| 4238STDY6426811 | 20280_8#143 | ERR1625445 |
| 4238STDY6426813 | 20280_8#145 | ERR1625447 |
| 4238STDY6426814 | 20280_8#146 | ERR1625448 |
| 4238STDY6426815 | 20280_8#147 | ERR1625449 |
| 4238STDY6426816 | 20280_8#148 | ERR1625450 |
| 4238STDY6426817 | 20280_8#149 | ERR1625451 |
| 4238STDY6426818 | 20280_8#150 | ERR1625452 |
| 4238STDY6426819 | 20280_8#151 | ERR1625453 |
| 4238STDY6426820 | 20280_8#152 | ERR1625454 |
| 4238STDY6426821 | 20280_8#153 | ERR1625455 |
| 4238STDY6426822 | 20280_8#154 | ERR1625456 |
| 4238STDY6426823 | 20280_8#155 | ERR1625457 |
| 4238STDY6426824 | 20280_8#156 | ERR1625458 |
| 4238STDY6426825 | 20280_8#157 | ERR1625459 |
| 4238STDY6426826 | 20280_8#158 | ERR1625460 |
| 4238STDY6426827 | 20280_8#159 | ERR1625461 |
| 4238STDY6426828 | 20280_8#160 | ERR1625462 |
| 4238STDY6426829 | 20280_8#161 | ERR1625463 |
| 4238STDY6426830 | 20280_8#162 | ERR1625464 |
| 4238STDY6426835 | 20280_8#167 | ERR1625469 |
| 4238STDY6426836 | 20280_8#168 | ERR1625470 |
| 4238STDY6426837 | 20280_8#169 | ERR1625471 |
| 4238STDY6426839 | 20280_8#171 | ERR1625473 |
| 4238STDY6426840 | 20280_8#172 | ERR1625474 |
| 4238STDY6426841 | 20280_8#173 | ERR1625475 |
| 4238STDY6426842 | 20280_8#174 | ERR1625476 |
| 4238STDY6426843 | 20280_8#175 | ERR1625477 |
| 4238STDY6426844 | 20280_8#176 | ERR1625478 |
| 4238STDY6426845 | 20280_8#177 | ERR1625479 |
| 4238STDY6426846 | 20280_8#178 | ERR1625480 |
| 4238STDY6426847 | 20280_8#179 | ERR1625481 |
| 4238STDY6426848 | 20280_8#180 | ERR1625482 |
| 4238STDY6426849 | 20280_8#181 | ERR1625483 |
| 4238STDY6426850 | 20280_8#182 | ERR1625484 |
| 4238STDY6426851 | 20280_8#183 | ERR1625485 |
| 4238STDY6426852 | 20280_8#184 | ERR1625486 |
| 4238STDY6426853 | 20280_8#185 | ERR1625487 |
| 4238STDY6426855 | 20280_8#187 | ERR1625489 |
| 4238STDY6426856 | 20280_8#188 | ERR1625490 |
| 4238STDY6426857 | 20280_8#189 | ERR1625491 |
| 4238STDY6445344 | 20416_6#1 | ERR1672372 |
| 4238STDY6445345 | 20416_6#2 | ERR1672373 |
| 4238STDY6445346 | 20416_6#3 | ERR1672374 |
| 4238STDY6445347 | 20416_6#4 | ERR1672375 |
| 4238STDY6445348 | 20416_6#5 | ERR1672376 |
| 4238STDY6445349 | 20416_6#6 | ERR1672377 |
| 4238STDY6445350 | 20416_6#7 | ERR1672378 |
| 4238STDY6445351 | 20416_6#8 | ERR1672379 |
| 4238STDY6445352 | 20416_6#9 | ERR1672380 |
| 4238STDY6445353 | 20416_6#10 | ERR1672381 |
| 4238STDY6445354 | 20416_6#11 | ERR1672382 |
| 4238STDY6445355 | 20416_6#12 | ERR1672383 |
| 4238STDY6445356 | 20416_6#13 | ERR1672384 |
| 4238STDY6445357 | 20416_6#14 | ERR1672385 |
| 4238STDY6445358 | 20416_6#15 | ERR1672386 |
| 4238STDY6445359 | 20416_6#16 | ERR1672387 |
| 4238STDY6445360 | 20416_6#17 | ERR1672388 |
| 4238STDY6445361 | 20416_6#18 | ERR1672389 |
| 4238STDY6445362 | 20416_6#19 | ERR1672390 |
| 4238STDY6445363 | 20416_6#20 | ERR1672391 |
| 4238STDY6445364 | 20416_6#21 | ERR1672392 |
| 4238STDY6445365 | 20416_6#22 | ERR1672393 |
| 4238STDY6445366 | 20416_6#23 | ERR1672394 |
| 4238STDY6445367 | 20416_6#24 | ERR1672395 |
| 4238STDY6445368 | 20416_6#25 | ERR1672396 |
| 4238STDY6445370 | 20416_6#27 | ERR1672398 |
| 4238STDY6445371 | 20416_6#28 | ERR1672399 |
| 4238STDY6445372 | 20416_6#29 | ERR1672400 |
| 4238STDY6445373 | 20416_6#30 | ERR1672401 |
| 4238STDY6445374 | 20416_6#31 | ERR1672402 |
| 4238STDY6445376 | 20416_6#33 | ERR1672404 |
| 4238STDY6445377 | 20416_6#34 | ERR1672405 |
| 4238STDY6445378 | 20416_6#35 | ERR1672406 |
| 4238STDY6445379 | 20416_6#36 | ERR1672407 |
| 4238STDY6445380 | 20416_6#37 | ERR1672408 |
| 4238STDY6445381 | 20416_6#38 | ERR1672409 |
| 4238STDY6445382 | 20416_6#39 | ERR1672410 |
| 4238STDY6445383 | 20416_6#40 | ERR1672411 |
| 4238STDY6445384 | 20416_6#41 | ERR1672412 |
| 4238STDY6445385 | 20416_6#42 | ERR1672413 |
| 4238STDY6445387 | 20416_6#44 | ERR1672415 |
| 4238STDY6445388 | 20416_6#45 | ERR1672416 |
| 4238STDY6445389 | 20416_6#46 | ERR1672417 |
| 4238STDY6445390 | 20416_6#47 | ERR1672418 |
| 4238STDY6445391 | 20416_6#48 | ERR1672419 |
| 4238STDY6445392 | 20416_6#49 | ERR1672420 |
| 4238STDY6445393 | 20416_6#50 | ERR1672421 |
| 4238STDY6445394 | 20416_6#51 | ERR1672422 |
| 4238STDY6445395 | 20416_6#52 | ERR1672423 |
| 4238STDY6445396 | 20416_6#53 | ERR1672424 |
| 4238STDY6445397 | 20416_6#54 | ERR1672425 |
| 4238STDY6445398 | 20416_6#55 | ERR1672426 |
| 4238STDY6445399 | 20416_6#56 | ERR1672427 |
| 4238STDY6445400 | 20416_6#57 | ERR1672428 |
| 4238STDY6445401 | 20416_6#58 | ERR1672429 |
| 4238STDY6445402 | 20416_6#59 | ERR1672430 |
| 4238STDY6445403 | 20416_6#60 | ERR1672431 |
| 4238STDY6445404 | 20416_6#61 | ERR1672432 |
| 4238STDY6445405 | 20416_6#62 | ERR1672433 |
| 4238STDY6445406 | 20416_6#63 | ERR1672434 |
| 4238STDY6445407 | 20416_6#64 | ERR1672435 |
| 4238STDY6445408 | 20416_6#65 | ERR1672436 |
| 4238STDY6445409 | 20416_6#66 | ERR1672437 |
| 4238STDY6445410 | 20416_6#67 | ERR1672438 |
| 4238STDY6445411 | 20416_6#68 | ERR1672439 |
| 4238STDY6445412 | 20416_6#69 | ERR1672440 |
| 4238STDY6445413 | 20416_6#70 | ERR1672441 |
| 4238STDY6445414 | 20416_6#71 | ERR1672442 |
| 4238STDY6445415 | 20416_6#72 | ERR1672443 |
| 4238STDY6445416 | 20416_6#73 | ERR1672444 |
| 4238STDY6445418 | 20416_6#75 | ERR1672446 |
| 4238STDY6445419 | 20416_6#76 | ERR1672447 |
| 4238STDY6445420 | 20416_6#77 | ERR1672448 |
| 4238STDY6445421 | 20416_6#78 | ERR1672449 |
| 4238STDY6445422 | 20416_6#79 | ERR1672450 |
| 4238STDY6445423 | 20416_6#80 | ERR1672451 |
| 4238STDY6445424 | 20416_6#81 | ERR1672452 |
| 4238STDY6445425 | 20416_6#82 | ERR1672453 |
| 4238STDY6445426 | 20416_6#83 | ERR1672454 |
| 4238STDY6445427 | 20416_6#84 | ERR1672455 |
| 4238STDY6445428 | 20416_6#85 | ERR1672456 |
| 4238STDY6445429 | 20416_6#86 | ERR1672457 |
| 4238STDY6445430 | 20416_6#87 | ERR1672458 |
| 4238STDY6445431 | 20416_6#88 | ERR1672459 |
| 4238STDY6445432 | 20416_6#89 | ERR1672460 |
| 4238STDY6445433 | 20416_6#90 | ERR1672461 |
| 4238STDY6445434 | 20416_6#91 | ERR1672462 |
| 4238STDY6445435 | 20416_6#92 | ERR1672463 |
| 4238STDY6445441 | 20416_6#94 | ERR1672465 |
| 4238STDY6445442 | 20416_6#95 | ERR1672466 |
| 4238STDY6445443 | 20416_6#96 | ERR1672467 |
| 4238STDY6445444 | 20416_6#97 | ERR1672468 |
| 4238STDY6445445 | 20416_6#98 | ERR1672469 |
| 4238STDY6445446 | 20416_6#99 | ERR1672470 |
| 4238STDY6445447 | 20416_6#100 | ERR1672471 |
| 4238STDY6445448 | 20416_6#101 | ERR1672472 |
| 4238STDY6445449 | 20416_6#102 | ERR1672473 |
| 4238STDY6445450 | 20416_6#103 | ERR1672474 |
| 4238STDY6445451 | 20416_6#104 | ERR1672475 |
| 4238STDY6445453 | 20416_6#106 | ERR1672477 |
| 4238STDY6445454 | 20416_6#107 | ERR1672478 |
| 4238STDY6445455 | 20416_6#108 | ERR1672479 |
| 4238STDY6445456 | 20416_6#109 | ERR1672480 |
| 4238STDY6445457 | 20416_6#110 | ERR1672481 |
| 4238STDY6445458 | 20416_6#111 | ERR1672482 |
| 4238STDY6445459 | 20416_6#112 | ERR1672483 |
| 4238STDY6445460 | 20416_6#113 | ERR1672484 |
| 4238STDY6445461 | 20416_6#114 | ERR1672485 |
| 4238STDY6445462 | 20416_6#115 | ERR1672486 |
| 4238STDY6445463 | 20416_6#116 | ERR1672487 |
| 4238STDY6445464 | 20416_6#117 | ERR1672488 |
| 4238STDY6445465 | 20416_6#118 | ERR1672489 |
| 4238STDY6445466 | 20416_6#119 | ERR1672490 |
| 4238STDY6445467 | 20416_6#120 | ERR1672491 |
| 4238STDY6445468 | 20416_6#121 | ERR1672492 |
| 4238STDY6445469 | 20416_6#122 | ERR1672493 |
| 4238STDY6445470 | 20416_6#123 | ERR1672494 |
| 4238STDY6445471 | 20416_6#124 | ERR1672495 |
| 4238STDY6445472 | 20416_6#125 | ERR1672496 |
| 4238STDY6445474 | 20416_6#127 | ERR1672498 |
| 4238STDY6445475 | 20416_6#128 | ERR1672499 |
| 4238STDY6445477 | 20416_6#130 | ERR1672501 |
| 4238STDY6445478 | 20416_6#131 | ERR1672502 |
| 4238STDY6445479 | 20416_6#132 | ERR1672503 |
| 4238STDY6445481 | 20416_6#134 | ERR1672505 |
| 4238STDY6445482 | 20416_6#135 | ERR1672506 |
| 4238STDY6445483 | 20416_6#136 | ERR1672507 |
| 4238STDY6445484 | 20416_6#137 | ERR1672508 |
| 4238STDY6445485 | 20416_6#138 | ERR1672509 |
| 4238STDY6445486 | 20416_6#139 | ERR1672510 |
| 4238STDY6445487 | 20416_6#140 | ERR1672511 |
| 4238STDY6445488 | 20416_6#141 | ERR1672512 |
| 4238STDY6445489 | 20416_6#142 | ERR1672513 |
| 4238STDY6445490 | 20416_6#143 | ERR1672514 |
| 4238STDY6445491 | 20416_6#144 | ERR1672515 |
| 4238STDY6445492 | 20416_6#145 | ERR1672516 |
| 4238STDY6445493 | 20416_6#146 | ERR1672517 |
| 4238STDY6445494 | 20416_6#147 | ERR1672518 |
| 4238STDY6445495 | 20416_6#148 | ERR1672519 |
| 4238STDY6445496 | 20416_6#149 | ERR1672520 |
| 4238STDY6445497 | 20416_6#150 | ERR1672521 |
| 4238STDY6445498 | 20416_6#151 | ERR1672522 |
| 4238STDY6445499 | 20416_6#152 | ERR1672523 |
| 4238STDY6445500 | 20416_6#153 | ERR1672524 |
| 4238STDY6445501 | 20416_6#154 | ERR1672525 |
| 4238STDY6445502 | 20416_6#155 | ERR1672526 |
| 4238STDY6445503 | 20416_6#156 | ERR1672527 |
| 4238STDY6445504 | 20416_6#157 | ERR1672528 |
| 4238STDY6445505 | 20416_6#158 | ERR1672529 |
| 4238STDY6445506 | 20416_6#159 | ERR1672530 |
| 4238STDY6445508 | 20416_6#161 | ERR1672532 |
| 4238STDY6445509 | 20416_6#162 | ERR1672533 |
| 4238STDY6445511 | 20416_6#164 | ERR1672535 |
| 4238STDY6445512 | 20416_6#165 | ERR1672536 |
| 4238STDY6445513 | 20416_6#166 | ERR1672537 |
| 4238STDY6445514 | 20416_6#167 | ERR1672538 |
| 4238STDY6445515 | 20416_6#168 | ERR1672539 |
| 4238STDY6445516 | 20416_6#169 | ERR1672540 |
| 4238STDY6445517 | 20416_6#170 | ERR1672541 |
| 4238STDY6445519 | 20416_6#172 | ERR1672543 |
| 4238STDY6445520 | 20416_6#173 | ERR1672544 |
| 4238STDY6445522 | 20416_6#175 | ERR1672546 |
| 4238STDY6445523 | 20416_6#176 | ERR1672547 |
| 4238STDY6445524 | 20416_6#177 | ERR1672548 |
| 4238STDY6445525 | 20416_6#178 | ERR1672549 |
| 4238STDY6445526 | 20416_6#179 | ERR1672550 |
| 4238STDY6445527 | 20416_6#180 | ERR1672551 |
| 4238STDY6445528 | 20416_6#181 | ERR1672552 |
| 4238STDY6445529 | 20416_6#182 | ERR1672553 |
| 4238STDY6445530 | 20416_6#183 | ERR1672554 |
| 4238STDY6445531 | 20416_6#184 | ERR1672555 |
| 4238STDY6445532 | 20416_6#185 | ERR1672556 |
| 4238STDY6445533 | 20416_6#186 | ERR1672557 |
| 4238STDY6445534 | 20416_6#187 | ERR1672558 |
| 4238STDY6445536 | 20416_6#188 | ERR1672559 |
| 4238STDY6445537 | 20416_6#189 | ERR1672560 |
| 4238STDY6445538 | 20416_7#1 | ERR1672561 |
| 4238STDY6445539 | 20416_7#2 | ERR1672562 |
| 4238STDY6445540 | 20416_7#3 | ERR1672563 |
| 4238STDY6445541 | 20416_7#4 | ERR1672564 |
| 4238STDY6445542 | 20416_7#5 | ERR1672565 |
| 4238STDY6445543 | 20416_7#6 | ERR1672566 |
| 4238STDY6445544 | 20416_7#7 | ERR1672567 |
| 4238STDY6445545 | 20416_7#8 | ERR1672568 |
| 4238STDY6445546 | 20416_7#9 | ERR1672569 |
| 4238STDY6445547 | 20416_7#10 | ERR1672570 |
| 4238STDY6445548 | 20416_7#11 | ERR1672571 |
| 4238STDY6445549 | 20416_7#12 | ERR1672572 |
| 4238STDY6445550 | 20416_7#13 | ERR1672573 |
| 4238STDY6445551 | 20416_7#14 | ERR1672574 |
| 4238STDY6445552 | 20416_7#15 | ERR1672575 |
| 4238STDY6445553 | 20416_7#16 | ERR1672576 |
| 4238STDY6445554 | 20416_7#17 | ERR1672577 |
| 4238STDY6445555 | 20416_7#18 | ERR1672578 |
| 4238STDY6445556 | 20416_7#19 | ERR1672579 |
| 4238STDY6445557 | 20416_7#20 | ERR1672580 |
| 4238STDY6445558 | 20416_7#21 | ERR1672581 |
| 4238STDY6445559 | 20416_7#22 | ERR1672582 |
| 4238STDY6445560 | 20416_7#23 | ERR1672583 |
| 4238STDY6445561 | 20416_7#24 | ERR1672584 |
| 4238STDY6445562 | 20416_7#25 | ERR1672585 |
| 4238STDY6445563 | 20416_7#26 | ERR1672586 |
| 4238STDY6445564 | 20416_7#27 | ERR1672587 |
| 4238STDY6445565 | 20416_7#28 | ERR1672588 |
| 4238STDY6445567 | 20416_7#30 | ERR1672590 |
| 4238STDY6445568 | 20416_7#31 | ERR1672591 |
| 4238STDY6445569 | 20416_7#32 | ERR1672592 |
| 4238STDY6445570 | 20416_7#33 | ERR1672593 |
| 4238STDY6445571 | 20416_7#34 | ERR1672594 |
| 4238STDY6445572 | 20416_7#35 | ERR1672595 |
| 4238STDY6445573 | 20416_7#36 | ERR1672596 |
| 4238STDY6445574 | 20416_7#37 | ERR1672597 |
| 4238STDY6445575 | 20416_7#38 | ERR1672598 |
| 4238STDY6445576 | 20416_7#39 | ERR1672599 |
| 4238STDY6445577 | 20416_7#40 | ERR1672600 |
| 4238STDY6445578 | 20416_7#41 | ERR1672601 |
| 4238STDY6445579 | 20416_7#42 | ERR1672602 |
| 4238STDY6445581 | 20416_7#44 | ERR1672604 |
| 4238STDY6445582 | 20416_7#45 | ERR1672605 |
| 4238STDY6445583 | 20416_7#46 | ERR1672606 |
| 4238STDY6445584 | 20416_7#47 | ERR1672607 |
| 4238STDY6445585 | 20416_7#48 | ERR1672608 |
| 4238STDY6445586 | 20416_7#49 | ERR1672609 |
| 4238STDY6445587 | 20416_7#50 | ERR1672610 |
| 4238STDY6445588 | 20416_7#51 | ERR1672611 |
| 4238STDY6445589 | 20416_7#52 | ERR1672612 |
| 4238STDY6445590 | 20416_7#53 | ERR1672613 |
| 4238STDY6445591 | 20416_7#54 | ERR1672614 |
| 4238STDY6445592 | 20416_7#55 | ERR1672615 |
| 4238STDY6445593 | 20416_7#56 | ERR1672616 |
| 4238STDY6445594 | 20416_7#57 | ERR1672617 |
| 4238STDY6445595 | 20416_7#58 | ERR1672618 |
| 4238STDY6445596 | 20416_7#59 | ERR1672619 |
| 4238STDY6445597 | 20416_7#60 | ERR1672620 |
| 4238STDY6445598 | 20416_7#61 | ERR1672621 |
| 4238STDY6445600 | 20416_7#63 | ERR1672623 |
| 4238STDY6445602 | 20416_7#65 | ERR1672625 |
| 4238STDY6445603 | 20416_7#66 | ERR1672626 |
| 4238STDY6445604 | 20416_7#67 | ERR1672627 |
| 4238STDY6445606 | 20416_7#69 | ERR1672629 |
| 4238STDY6445607 | 20416_7#70 | ERR1672630 |
| 4238STDY6445609 | 20416_7#72 | ERR1672632 |
| 4238STDY6445610 | 20416_7#73 | ERR1672633 |
| 4238STDY6445611 | 20416_7#74 | ERR1672634 |
| 4238STDY6445612 | 20416_7#75 | ERR1672635 |
| 4238STDY6445613 | 20416_7#76 | ERR1672636 |
| 4238STDY6445614 | 20416_7#77 | ERR1672637 |
| 4238STDY6445615 | 20416_7#78 | ERR1672638 |
| 4238STDY6445616 | 20416_7#79 | ERR1672639 |
| 4238STDY6445617 | 20416_7#80 | ERR1672640 |
| 4238STDY6445618 | 20416_7#81 | ERR1672641 |
| 4238STDY6445619 | 20416_7#82 | ERR1672642 |
| 4238STDY6445620 | 20416_7#83 | ERR1672643 |
| 4238STDY6445621 | 20416_7#84 | ERR1672644 |
| 4238STDY6445622 | 20416_7#85 | ERR1672645 |
| 4238STDY6445623 | 20416_7#86 | ERR1672646 |
| 4238STDY6445624 | 20416_7#87 | ERR1672647 |
| 4238STDY6445625 | 20416_7#88 | ERR1672648 |
| 4238STDY6445627 | 20416_7#90 | ERR1672650 |
| 4238STDY6445628 | 20416_7#91 | ERR1672651 |
| 4238STDY6445629 | 20416_7#92 | ERR1672652 |
| 4238STDY6445630 | 20416_7#93 | ERR1672653 |
| 4238STDY6445632 | 20416_7#94 | ERR1672654 |
| 4238STDY6445633 | 20416_7#95 | ERR1672655 |
| 4238STDY6445634 | 20416_7#96 | ERR1672656 |
| 4238STDY6445635 | 20416_7#97 | ERR1672657 |
| 4238STDY6445636 | 20416_7#98 | ERR1672658 |
| 4238STDY6445637 | 20416_7#99 | ERR1672659 |
| 4238STDY6445638 | 20416_7#100 | ERR1672660 |
| 4238STDY6445639 | 20416_7#101 | ERR1672661 |
| 4238STDY6445640 | 20416_7#102 | ERR1672662 |
| 4238STDY6445642 | 20416_7#104 | ERR1672664 |
| 4238STDY6445643 | 20416_7#105 | ERR1672665 |
| 4238STDY6445644 | 20416_7#106 | ERR1672666 |
| 4238STDY6445645 | 20416_7#107 | ERR1672667 |
| 4238STDY6445646 | 20416_7#108 | ERR1672668 |
| 4238STDY6445648 | 20416_7#110 | ERR1672670 |
| 4238STDY6445649 | 20416_7#111 | ERR1672671 |
| 4238STDY6445650 | 20416_7#112 | ERR1672672 |
| 4238STDY6445651 | 20416_7#113 | ERR1672673 |
| 4238STDY6445652 | 20416_7#114 | ERR1672674 |
| 4238STDY6445653 | 20416_7#115 | ERR1672675 |
| 4238STDY6445655 | 20416_7#117 | ERR1672677 |
| 4238STDY6445656 | 20416_7#118 | ERR1672678 |
| 4238STDY6445657 | 20416_7#119 | ERR1672679 |
| 4238STDY6445658 | 20416_7#120 | ERR1672680 |
| 4238STDY6445659 | 20416_7#121 | ERR1672681 |
| 4238STDY6445660 | 20416_7#122 | ERR1672682 |
| 4238STDY6445661 | 20416_7#123 | ERR1672683 |
| 4238STDY6445662 | 20416_7#124 | ERR1672684 |
| 4238STDY6445663 | 20416_7#125 | ERR1672685 |
| 4238STDY6445664 | 20416_7#126 | ERR1672686 |
| 4238STDY6445665 | 20416_7#127 | ERR1672687 |
| 4238STDY6445666 | 20416_7#128 | ERR1672688 |
| 4238STDY6445667 | 20416_7#129 | ERR1672689 |
| 4238STDY6445668 | 20416_7#130 | ERR1672690 |
| 4238STDY6445669 | 20416_7#131 | ERR1672691 |
| 4238STDY6445670 | 20416_7#132 | ERR1672692 |
| 4238STDY6445671 | 20416_7#133 | ERR1672693 |
| 4238STDY6445672 | 20416_7#134 | ERR1672694 |
| 4238STDY6445673 | 20416_7#135 | ERR1672695 |
| 4238STDY6445674 | 20416_7#136 | ERR1672696 |
| 4238STDY6445675 | 20416_7#137 | ERR1672697 |
| 4238STDY6445676 | 20416_7#138 | ERR1672698 |
| 4238STDY6445677 | 20416_7#139 | ERR1672699 |
| 4238STDY6445678 | 20416_7#140 | ERR1672700 |
| 4238STDY6445679 | 20416_7#141 | ERR1672701 |
| 4238STDY6445680 | 20416_7#142 | ERR1672702 |
| 4238STDY6445681 | 20416_7#143 | ERR1672703 |
| 4238STDY6445682 | 20416_7#144 | ERR1672704 |
| 4238STDY6445683 | 20416_7#145 | ERR1672705 |
| 4238STDY6445684 | 20416_7#146 | ERR1672706 |
| 4238STDY6445685 | 20416_7#147 | ERR1672707 |
| 4238STDY6445686 | 20416_7#148 | ERR1672708 |
| 4238STDY6445689 | 20416_7#151 | ERR1672711 |
| 4238STDY6445690 | 20416_7#152 | ERR1672712 |
| 4238STDY6445691 | 20416_7#153 | ERR1672713 |
| 4238STDY6445692 | 20416_7#154 | ERR1672714 |
| 4238STDY6445693 | 20416_7#155 | ERR1672715 |
| 4238STDY6445694 | 20416_7#156 | ERR1672716 |
| 4238STDY6445696 | 20416_7#158 | ERR1672718 |
| 4238STDY6445697 | 20416_7#159 | ERR1672719 |
| 4238STDY6445698 | 20416_7#160 | ERR1672720 |
| 4238STDY6445699 | 20416_7#161 | ERR1672721 |
| 4238STDY6445700 | 20416_7#162 | ERR1672722 |
| 4238STDY6445701 | 20416_7#163 | ERR1672723 |
| 4238STDY6445702 | 20416_7#164 | ERR1672724 |
| 4238STDY6445703 | 20416_7#165 | ERR1672725 |
| 4238STDY6445706 | 20416_7#168 | ERR1672728 |
| 4238STDY6445707 | 20416_7#169 | ERR1672729 |
| 4238STDY6445708 | 20416_7#170 | ERR1672730 |
| 4238STDY6445709 | 20416_7#171 | ERR1672731 |
| 4238STDY6445710 | 20416_7#172 | ERR1672732 |
| 4238STDY6445711 | 20416_7#173 | ERR1672733 |
| 4238STDY6445712 | 20416_7#174 | ERR1672734 |
| 4238STDY6445713 | 20416_7#175 | ERR1672735 |
| 4238STDY6445714 | 20416_7#176 | ERR1672736 |
| 4238STDY6445715 | 20416_7#177 | ERR1672737 |
| 4238STDY6445716 | 20416_7#178 | ERR1672738 |
| 4238STDY6445717 | 20416_7#179 | ERR1672739 |
| 4238STDY6445718 | 20416_7#180 | ERR1672740 |
| 4238STDY6445719 | 20416_7#181 | ERR1672741 |
| 4238STDY6445720 | 20416_7#182 | ERR1672742 |
| 4238STDY6445721 | 20416_7#183 | ERR1672743 |
| 4238STDY6445722 | 20416_7#184 | ERR1672744 |
| 4238STDY6445723 | 20416_7#185 | ERR1672745 |
| 4238STDY6445724 | 20416_7#186 | ERR1672746 |
| 4238STDY6445725 | 20416_7#187 | ERR1672747 |
| 4238STDY6445726 | 20416_7#188 | ERR1672748 |
| 4238STDY6445728 | 20416_8#1 | ERR1672749 |
| 4238STDY6445729 | 20416_8#2 | ERR1672750 |
| 4238STDY6445730 | 20416_8#3 | ERR1672751 |
| 4238STDY6445731 | 20416_8#4 | ERR1672752 |
| 4238STDY6445732 | 20416_8#5 | ERR1672753 |
| 4238STDY6445734 | 20416_8#7 | ERR1672755 |
| 4238STDY6445735 | 20416_8#8 | ERR1672756 |
| 4238STDY6445736 | 20416_8#9 | ERR1672757 |
| 4238STDY6445737 | 20416_8#10 | ERR1672758 |
| 4238STDY6445738 | 20416_8#11 | ERR1672759 |
| 4238STDY6445739 | 20416_8#12 | ERR1672760 |
| 4238STDY6445740 | 20416_8#13 | ERR1672761 |
| 4238STDY6445741 | 20416_8#14 | ERR1672762 |
| 4238STDY6445742 | 20416_8#15 | ERR1672763 |
| 4238STDY6445744 | 20416_8#17 | ERR1672765 |
| 4238STDY6445745 | 20416_8#18 | ERR1672766 |
| 4238STDY6445746 | 20416_8#19 | ERR1672767 |
| 4238STDY6445747 | 20416_8#20 | ERR1672768 |
| 4238STDY6445748 | 20416_8#21 | ERR1672769 |
| 4238STDY6445749 | 20416_8#22 | ERR1672770 |
| 4238STDY6445750 | 20416_8#23 | ERR1672771 |
| 4238STDY6445751 | 20416_8#24 | ERR1672772 |
| 4238STDY6445752 | 20416_8#25 | ERR1672773 |
| 4238STDY6445753 | 20416_8#26 | ERR1672774 |
| 4238STDY6445754 | 20416_8#27 | ERR1672775 |
| 4238STDY6445758 | 20416_8#31 | ERR1672779 |
| 4238STDY6445759 | 20416_8#32 | ERR1672780 |
| 4238STDY6445760 | 20416_8#33 | ERR1672781 |
| 4238STDY6445762 | 20416_8#35 | ERR1672783 |
| 4238STDY6445763 | 20416_8#36 | ERR1672784 |
| 4238STDY6445764 | 20416_8#37 | ERR1672785 |
| 4238STDY6445765 | 20416_8#38 | ERR1672786 |
| 4238STDY6445767 | 20416_8#40 | ERR1672788 |
| 4238STDY6445768 | 20416_8#41 | ERR1672789 |
| 4238STDY6445769 | 20416_8#42 | ERR1672790 |
| 4238STDY6445770 | 20416_8#43 | ERR1672791 |
| 4238STDY6445771 | 20416_8#44 | ERR1672792 |
| 4238STDY6445772 | 20416_8#45 | ERR1672793 |
| 4238STDY6445773 | 20416_8#46 | ERR1672794 |
| 4238STDY6445774 | 20416_8#47 | ERR1672795 |
| 4238STDY6445775 | 20416_8#48 | ERR1672796 |
| 4238STDY6445776 | 20416_8#49 | ERR1672797 |
| 4238STDY6445777 | 20416_8#50 | ERR1672798 |
| 4238STDY6445778 | 20416_8#51 | ERR1672799 |
| 4238STDY6445779 | 20416_8#52 | ERR1672800 |
| 4238STDY6445780 | 20416_8#53 | ERR1672801 |
| 4238STDY6445781 | 20416_8#54 | ERR1672802 |
| 4238STDY6445782 | 20416_8#55 | ERR1672803 |
| 4238STDY6445783 | 20416_8#56 | ERR1672804 |
| 4238STDY6445784 | 20416_8#57 | ERR1672805 |
| 4238STDY6445785 | 20416_8#58 | ERR1672806 |
| 4238STDY6445786 | 20416_8#59 | ERR1672807 |
| 4238STDY6445787 | 20416_8#60 | ERR1672808 |
| 4238STDY6445788 | 20416_8#61 | ERR1672809 |
| 4238STDY6445789 | 20416_8#62 | ERR1672810 |
| 4238STDY6445790 | 20416_8#63 | ERR1672811 |
| 4238STDY6445791 | 20416_8#64 | ERR1672812 |
| 4238STDY6445792 | 20416_8#65 | ERR1672813 |
| 4238STDY6445793 | 20416_8#66 | ERR1672814 |
| 4238STDY6445794 | 20416_8#67 | ERR1672815 |
| 4238STDY6445795 | 20416_8#68 | ERR1672816 |
| 4238STDY6445796 | 20416_8#69 | ERR1672817 |
| 4238STDY6445797 | 20416_8#70 | ERR1672818 |
| 4238STDY6445798 | 20416_8#71 | ERR1672819 |
| 4238STDY6445799 | 20416_8#72 | ERR1672820 |
| 4238STDY6445800 | 20416_8#73 | ERR1672821 |
| 4238STDY6445801 | 20416_8#74 | ERR1672822 |
| 4238STDY6445802 | 20416_8#75 | ERR1672823 |
| 4238STDY6445803 | 20416_8#76 | ERR1672824 |
| 4238STDY6445804 | 20416_8#77 | ERR1672825 |
| 4238STDY6445805 | 20416_8#78 | ERR1672826 |
| 4238STDY6445806 | 20416_8#79 | ERR1672827 |
| 4238STDY6445807 | 20416_8#80 | ERR1672828 |
| 4238STDY6445808 | 20416_8#81 | ERR1672829 |
| 4238STDY6445809 | 20416_8#82 | ERR1672830 |
| 4238STDY6445811 | 20416_8#84 | ERR1672832 |
| 4238STDY6445812 | 20416_8#85 | ERR1672833 |
| 4238STDY6445813 | 20416_8#86 | ERR1672834 |
| 4238STDY6445814 | 20416_8#87 | ERR1672835 |
| 4238STDY6445815 | 20416_8#88 | ERR1672836 |
| 4238STDY6445816 | 20416_8#89 | ERR1672837 |
| 4238STDY6445817 | 20416_8#90 | ERR1672838 |
| 4238STDY6445818 | 20416_8#91 | ERR1672839 |
| 4238STDY6445819 | 20416_8#92 | ERR1672840 |
| 4238STDY6445820 | 20416_8#93 | ERR1672841 |
| 4238STDY6445824 | 20416_8#94 | ERR1672842 |
| 4238STDY6445825 | 20416_8#95 | ERR1672843 |
| 4238STDY6445826 | 20416_8#96 | ERR1672844 |
| 4238STDY6445827 | 20416_8#97 | ERR1672845 |
| 4238STDY6445828 | 20416_8#98 | ERR1672846 |
| 4238STDY6445829 | 20416_8#99 | ERR1672847 |
| 4238STDY6445830 | 20416_8#100 | ERR1672848 |
| 4238STDY6445831 | 20416_8#101 | ERR1672849 |
| 4238STDY6445832 | 20416_8#102 | ERR1672850 |
| 4238STDY6445833 | 20416_8#103 | ERR1672851 |
| 4238STDY6445834 | 20416_8#104 | ERR1672852 |
| 4238STDY6445835 | 20416_8#105 | ERR1672853 |
| 4238STDY6445836 | 20416_8#106 | ERR1672854 |
| 4238STDY6445837 | 20416_8#107 | ERR1672855 |
| 4238STDY6445838 | 20416_8#108 | ERR1672856 |
| 4238STDY6445839 | 20416_8#109 | ERR1672857 |
| 4238STDY6445841 | 20416_8#111 | ERR1672859 |
| 4238STDY6445842 | 20416_8#112 | ERR1672860 |
| 4238STDY6445844 | 20416_8#114 | ERR1672862 |
| 4238STDY6445845 | 20416_8#115 | ERR1672863 |
| 4238STDY6445846 | 20416_8#116 | ERR1672864 |
| 4238STDY6445847 | 20416_8#117 | ERR1672865 |
| 4238STDY6445848 | 20416_8#118 | ERR1672866 |
| 4238STDY6445849 | 20416_8#119 | ERR1672867 |
| 4238STDY6445850 | 20416_8#120 | ERR1672868 |
| 4238STDY6445851 | 20416_8#121 | ERR1672869 |
| 4238STDY6445852 | 20416_8#122 | ERR1672870 |
| 4238STDY6445853 | 20416_8#123 | ERR1672871 |
| 4238STDY6445854 | 20416_8#124 | ERR1672872 |
| 4238STDY6445855 | 20416_8#125 | ERR1672873 |
| 4238STDY6445856 | 20416_8#126 | ERR1672874 |
| 4238STDY6445857 | 20416_8#127 | ERR1672875 |
| 4238STDY6445858 | 20416_8#128 | ERR1672876 |
| 4238STDY6445859 | 20416_8#129 | ERR1672877 |
| 4238STDY6445860 | 20416_8#130 | ERR1672878 |
| 4238STDY6445862 | 20416_8#132 | ERR1672880 |
| 4238STDY6445863 | 20416_8#133 | ERR1672881 |
| 4238STDY6445864 | 20416_8#134 | ERR1672882 |
| 4238STDY6445865 | 20416_8#135 | ERR1672883 |
| 4238STDY6445866 | 20416_8#136 | ERR1672884 |
| 4238STDY6445867 | 20416_8#137 | ERR1672885 |
| 4238STDY6445868 | 20416_8#138 | ERR1672886 |
| 4238STDY6445869 | 20416_8#139 | ERR1672887 |
| 4238STDY6445870 | 20416_8#140 | ERR1672888 |
| 4238STDY6445871 | 20416_8#141 | ERR1672889 |
| 4238STDY6445872 | 20416_8#142 | ERR1672890 |
| 4238STDY6445873 | 20416_8#143 | ERR1672891 |
| 4238STDY6445874 | 20416_8#144 | ERR1672892 |
| 4238STDY6445876 | 20416_8#146 | ERR1672894 |
| 4238STDY6445877 | 20416_8#147 | ERR1672895 |
| 4238STDY6445878 | 20416_8#148 | ERR1672896 |
| 4238STDY6445879 | 20416_8#149 | ERR1672897 |
| 4238STDY6445880 | 20416_8#150 | ERR1672898 |
| 4238STDY6445881 | 20416_8#151 | ERR1672899 |
| 4238STDY6445882 | 20416_8#152 | ERR1672900 |
| 4238STDY6445883 | 20416_8#153 | ERR1672901 |
| 4238STDY6445884 | 20416_8#154 | ERR1672902 |
| 4238STDY6445885 | 20416_8#155 | ERR1672903 |
| 4238STDY6445886 | 20416_8#156 | ERR1672904 |
| 4238STDY6445887 | 20416_8#157 | ERR1672905 |
| 4238STDY6445888 | 20416_8#158 | ERR1672906 |
| 4238STDY6445889 | 20416_8#159 | ERR1672907 |
| 4238STDY6445890 | 20416_8#160 | ERR1672908 |
| 4238STDY6445891 | 20416_8#161 | ERR1672909 |
| 4238STDY6445892 | 20416_8#162 | ERR1672910 |
| 4238STDY6445894 | 20416_8#164 | ERR1672912 |
| 4238STDY6445895 | 20416_8#165 | ERR1672913 |
| 4238STDY6445896 | 20416_8#166 | ERR1672914 |
| 4238STDY6445897 | 20416_8#167 | ERR1672915 |
| 4238STDY6445898 | 20416_8#168 | ERR1672916 |
| 4238STDY6445899 | 20416_8#169 | ERR1672917 |
| 4238STDY6445900 | 20416_8#170 | ERR1672918 |
| 4238STDY6445901 | 20416_8#171 | ERR1672919 |
| 4238STDY6445903 | 20416_8#173 | ERR1672921 |
| 4238STDY6445904 | 20416_8#174 | ERR1672922 |
| 4238STDY6445905 | 20416_8#175 | ERR1672923 |
| 4238STDY6445906 | 20416_8#176 | ERR1672924 |
| 4238STDY6445907 | 20416_8#177 | ERR1672925 |
| 4238STDY6445908 | 20416_8#178 | ERR1672926 |
| 4238STDY6445909 | 20416_8#179 | ERR1672927 |
| 4238STDY6445910 | 20416_8#180 | ERR1672928 |
| 4238STDY6445911 | 20416_8#181 | ERR1672929 |
| 4238STDY6445912 | 20416_8#182 | ERR1672930 |
| 4238STDY6445913 | 20416_8#183 | ERR1672931 |
| 4238STDY6445914 | 20416_8#184 | ERR1672932 |
| 4238STDY6445915 | 20416_8#185 | ERR1672933 |
| 4238STDY6445916 | 20416_8#186 | ERR1672934 |
| 4238STDY6445917 | 20416_8#187 | ERR1672935 |
| 4238STDY6445918 | 20416_8#188 | ERR1672936 |
| 4238STDY6445920 | 20402_3#1 | ERR1659680 |
| 4238STDY6445921 | 20402_3#2 | ERR1659681 |
| 4238STDY6445923 | 20402_3#4 | ERR1659683 |
| 4238STDY6445924 | 20402_3#5 | ERR1659684 |
| 4238STDY6445925 | 20402_3#6 | ERR1659685 |
| 4238STDY6445926 | 20402_3#7 | ERR1659686 |
| 4238STDY6445927 | 20402_3#8 | ERR1659687 |
| 4238STDY6445928 | 20402_3#9 | ERR1659688 |
| 4238STDY6445929 | 20402_3#10 | ERR1659689 |
| 4238STDY6445930 | 20402_3#11 | ERR1659690 |
| 4238STDY6445931 | 20402_3#12 | ERR1659691 |
| 4238STDY6445932 | 20402_3#13 | ERR1659692 |
| 4238STDY6445933 | 20402_3#14 | ERR1659693 |
| 4238STDY6445934 | 20402_3#15 | ERR1659694 |
| 4238STDY6445935 | 20402_3#16 | ERR1659695 |
| 4238STDY6445936 | 20402_3#17 | ERR1659696 |
| 4238STDY6445937 | 20402_3#18 | ERR1659697 |
| 4238STDY6445938 | 20402_3#19 | ERR1659698 |
| 4238STDY6445939 | 20402_3#20 | ERR1659699 |
| 4238STDY6445940 | 20402_3#21 | ERR1659700 |
| 4238STDY6445941 | 20402_3#22 | ERR1659701 |
| 4238STDY6445942 | 20402_3#23 | ERR1659702 |
| 4238STDY6445943 | 20402_3#24 | ERR1659703 |
| 4238STDY6445944 | 20402_3#25 | ERR1659704 |
| 4238STDY6445945 | 20402_3#26 | ERR1659705 |
| 4238STDY6445946 | 20402_3#27 | ERR1659706 |
| 4238STDY6445947 | 20402_3#28 | ERR1659707 |
| 4238STDY6445948 | 20402_3#29 | ERR1659708 |
| 4238STDY6445949 | 20402_3#30 | ERR1659709 |
| 4238STDY6445951 | 20402_3#32 | ERR1659711 |
| 4238STDY6445952 | 20402_3#33 | ERR1659712 |
| 4238STDY6445953 | 20402_3#34 | ERR1659713 |
| 4238STDY6445954 | 20402_3#35 | ERR1659714 |
| 4238STDY6445955 | 20402_3#36 | ERR1659715 |
| 4238STDY6445956 | 20402_3#37 | ERR1659716 |
| 4238STDY6445957 | 20402_3#38 | ERR1659717 |
| 4238STDY6445959 | 20402_3#40 | ERR1659719 |
| 4238STDY6445960 | 20402_3#41 | ERR1659720 |
| 4238STDY6445961 | 20402_3#42 | ERR1659721 |
| 4238STDY6445962 | 20402_3#43 | ERR1659722 |
| 4238STDY6445963 | 20402_3#44 | ERR1659723 |
| 4238STDY6445964 | 20402_3#45 | ERR1659724 |
| 4238STDY6445965 | 20402_3#46 | ERR1659725 |
| 4238STDY6445966 | 20402_3#47 | ERR1659726 |
| 4238STDY6445967 | 20402_3#48 | ERR1659727 |
| 4238STDY6445968 | 20402_3#49 | ERR1659728 |
| 4238STDY6445969 | 20402_3#50 | ERR1659729 |
| 4238STDY6445970 | 20402_3#51 | ERR1659730 |
| 4238STDY6445971 | 20402_3#52 | ERR1659731 |
| 4238STDY6445972 | 20402_3#53 | ERR1659732 |
| 4238STDY6445973 | 20402_3#54 | ERR1659733 |
| 4238STDY6445975 | 20402_3#56 | ERR1659735 |
| 4238STDY6445976 | 20402_3#57 | ERR1659736 |
| 4238STDY6445977 | 20402_3#58 | ERR1659737 |
| 4238STDY6445978 | 20402_3#59 | ERR1659738 |
| 4238STDY6445979 | 20402_3#60 | ERR1659739 |
| 4238STDY6445980 | 20402_3#61 | ERR1659740 |
| 4238STDY6445981 | 20402_3#62 | ERR1659741 |
| 4238STDY6445982 | 20402_3#63 | ERR1659742 |
| 4238STDY6445983 | 20402_3#64 | ERR1659743 |
| 4238STDY6445984 | 20402_3#65 | ERR1659744 |
| 4238STDY6445985 | 20402_3#66 | ERR1659745 |
| 4238STDY6445986 | 20402_3#67 | ERR1659746 |
| 4238STDY6445987 | 20402_3#68 | ERR1659747 |
| 4238STDY6445988 | 20402_3#69 | ERR1659748 |
| 4238STDY6445989 | 20402_3#70 | ERR1659749 |
| 4238STDY6445990 | 20402_3#71 | ERR1659750 |
| 4238STDY6445991 | 20402_3#72 | ERR1659751 |
| 4238STDY6445992 | 20402_3#73 | ERR1659752 |
| 4238STDY6445993 | 20402_3#74 | ERR1659753 |
| 4238STDY6445994 | 20402_3#75 | ERR1659754 |
| 4238STDY6445995 | 20402_3#76 | ERR1659755 |
| 4238STDY6445996 | 20402_3#77 | ERR1659756 |
| 4238STDY6445997 | 20402_3#78 | ERR1659757 |
| 4238STDY6445998 | 20402_3#79 | ERR1659758 |
| 4238STDY6445999 | 20402_3#80 | ERR1659759 |
| 4238STDY6446000 | 20402_3#81 | ERR1659760 |
| 4238STDY6446001 | 20402_3#82 | ERR1659761 |
| 4238STDY6446002 | 20402_3#83 | ERR1659762 |
| 4238STDY6446003 | 20402_3#84 | ERR1659763 |
| 4238STDY6446004 | 20402_3#85 | ERR1659764 |
| 4238STDY6446005 | 20402_3#86 | ERR1659765 |
| 4238STDY6446006 | 20402_3#87 | ERR1659766 |
| 4238STDY6446007 | 20402_3#88 | ERR1659767 |
| 4238STDY6446009 | 20402_3#90 | ERR1659769 |
| 4238STDY6446010 | 20402_3#91 | ERR1659770 |
| 4238STDY6446011 | 20402_3#92 | ERR1659771 |
| 4238STDY6446012 | 20402_3#93 | ERR1659772 |
| 4238STDY6446013 | 20402_3#94 | ERR1659773 |
| 4238STDY6446014 | 20402_3#95 | ERR1659774 |
| 4238STDY6446016 | 20402_3#96 | ERR1659775 |
| 4238STDY6446017 | 20402_3#97 | ERR1659776 |
| 4238STDY6446018 | 20402_3#98 | ERR1659777 |
| 4238STDY6446019 | 20402_3#99 | ERR1659778 |
| 4238STDY6446020 | 20402_3#100 | ERR1659779 |
| 4238STDY6446021 | 20402_3#101 | ERR1659780 |
| 4238STDY6446022 | 20402_3#102 | ERR1659781 |
| 4238STDY6446023 | 20402_3#103 | ERR1659782 |
| 4238STDY6446024 | 20402_3#104 | ERR1659783 |
| 4238STDY6446025 | 20402_3#105 | ERR1659784 |
| 4238STDY6446026 | 20402_3#106 | ERR1659785 |
| 4238STDY6446027 | 20402_3#107 | ERR1659786 |
| 4238STDY6446028 | 20402_3#108 | ERR1659787 |
| 4238STDY6446029 | 20402_3#109 | ERR1659788 |
| 4238STDY6446030 | 20402_3#110 | ERR1659789 |
| 4238STDY6446031 | 20402_3#111 | ERR1659790 |
| 4238STDY6446032 | 20402_3#112 | ERR1659791 |
| 4238STDY6446033 | 20402_3#113 | ERR1659792 |
| 4238STDY6446034 | 20402_3#114 | ERR1659793 |
| 4238STDY6446035 | 20402_3#115 | ERR1659794 |
| 4238STDY6446036 | 20402_3#116 | ERR1659795 |
| 4238STDY6446037 | 20402_3#117 | ERR1659796 |
| 4238STDY6446038 | 20402_3#118 | ERR1659797 |
| 4238STDY6446039 | 20402_3#119 | ERR1659798 |
| 4238STDY6446040 | 20402_3#120 | ERR1659799 |
| 4238STDY6446042 | 20402_3#122 | ERR1659801 |
| 4238STDY6446043 | 20402_3#123 | ERR1659802 |
| 4238STDY6446044 | 20402_3#124 | ERR1659803 |
| 4238STDY6446045 | 20402_3#125 | ERR1659804 |
| 4238STDY6446046 | 20402_3#126 | ERR1659805 |
| 4238STDY6446047 | 20402_3#127 | ERR1659806 |
| 4238STDY6446049 | 20402_3#129 | ERR1659808 |
| 4238STDY6446050 | 20402_3#130 | ERR1659809 |
| 4238STDY6446051 | 20402_3#131 | ERR1659810 |
| 4238STDY6446052 | 20402_3#132 | ERR1659811 |
| 4238STDY6446053 | 20402_3#133 | ERR1659812 |
| 4238STDY6446055 | 20402_3#135 | ERR1659814 |
| 4238STDY6446057 | 20402_3#137 | ERR1659816 |
| 4238STDY6446058 | 20402_3#138 | ERR1659817 |
| 4238STDY6446059 | 20402_3#139 | ERR1659818 |
| 4238STDY6446060 | 20402_3#140 | ERR1659819 |
| 4238STDY6446061 | 20402_3#141 | ERR1659820 |
| 4238STDY6446062 | 20402_3#142 | ERR1659821 |
| 4238STDY6446063 | 20402_3#143 | ERR1659822 |
| 4238STDY6446064 | 20402_3#144 | ERR1659823 |
| 4238STDY6446066 | 20402_3#146 | ERR1659825 |
| 4238STDY6446067 | 20402_3#147 | ERR1659826 |
| 4238STDY6446068 | 20402_3#148 | ERR1659827 |
| 4238STDY6446069 | 20402_3#149 | ERR1659828 |
| 4238STDY6446071 | 20402_3#151 | ERR1659830 |
| 4238STDY6446072 | 20402_3#152 | ERR1659831 |
| 4238STDY6446073 | 20402_3#153 | ERR1659832 |
| 4238STDY6446074 | 20402_3#154 | ERR1659833 |
| 4238STDY6446075 | 20402_3#155 | ERR1659834 |
| 4238STDY6446076 | 20402_3#156 | ERR1659835 |
| 4238STDY6446077 | 20402_3#157 | ERR1659836 |
| 4238STDY6446078 | 20402_3#158 | ERR1659837 |
| 4238STDY6446079 | 20402_3#159 | ERR1659838 |
| 4238STDY6446080 | 20402_3#160 | ERR1659839 |
| 4238STDY6446081 | 20402_3#161 | ERR1659840 |
| 4238STDY6446082 | 20402_3#162 | ERR1659841 |
| 4238STDY6446083 | 20402_3#163 | ERR1659842 |
| 4238STDY6446084 | 20402_3#164 | ERR1659843 |
| 4238STDY6446085 | 20402_3#165 | ERR1659844 |
| 4238STDY6446086 | 20402_3#166 | ERR1659845 |
| 4238STDY6446087 | 20402_3#167 | ERR1659846 |
| 4238STDY6446088 | 20402_3#168 | ERR1659847 |
| 4238STDY6446089 | 20402_3#169 | ERR1659848 |
| 4238STDY6446090 | 20402_3#170 | ERR1659849 |
| 4238STDY6446091 | 20402_3#171 | ERR1659850 |
| 4238STDY6446092 | 20402_3#172 | ERR1659851 |
| 4238STDY6446095 | 20402_3#175 | ERR1659854 |
| 4238STDY6446097 | 20402_3#177 | ERR1659856 |
| 4238STDY6446098 | 20402_3#178 | ERR1659857 |
| 4238STDY6446099 | 20402_3#179 | ERR1659858 |
| 4238STDY6446100 | 20402_3#180 | ERR1659859 |
| 4238STDY6446101 | 20402_3#181 | ERR1659860 |
| 4238STDY6446102 | 20402_3#182 | ERR1659861 |
| 4238STDY6446103 | 20402_3#183 | ERR1659862 |
| 4238STDY6446104 | 20402_3#184 | ERR1659863 |
| 4238STDY6446105 | 20402_3#185 | ERR1659864 |
| 4238STDY6446106 | 20402_3#186 | ERR1659865 |
| 4238STDY6446107 | 20402_3#187 | ERR1659866 |
| 4238STDY6446108 | 20402_3#188 | ERR1659867 |
| 6623STDY11015433 | 43525_2#3 | ERR10854209 |
| 6623STDY11015434 | 43525_2#5 | ERR10854211 |
| 6623STDY11015435 | 43525_2#7 | ERR10854213 |
| 6623STDY11015436 | 43525_2#9 | ERR10854215 |
| 6623STDY11015437 | 43525_2#11 | ERR10854217 |
| 6623STDY11015439 | 43525_2#15 | ERR10854221 |
| 6623STDY11015441 | 43525_2#35 | ERR10854225 |
| 6623STDY11015443 | 43525_2#39 | ERR10854229 |
| 6623STDY11015444 | 43525_2#41 | ERR10854231 |
| 6623STDY11015447 | 43525_2#47 | ERR10854238 |
| 6623STDY11015448 | 43525_2#65 | ERR10854240 |
| 6623STDY11015449 | 43525_2#67 | ERR10854242 |
| 6623STDY11015450 | 43525_2#69 | ERR10854244 |
| 6623STDY11015452 | 43525_2#73 | ERR10854248 |
| 6623STDY11015453 | 43525_2#75 | ERR10854250 |
| 6623STDY11015454 | 43525_2#1 | ERR10854207 |
| 6623STDY11015455 | 43525_2#77 | ERR10854252 |
| 6623STDY11015457 | 43525_2#97 | ERR10854256 |
| 6623STDY11015458 | 43525_2#99 | ERR10854258 |
| 6623STDY11015459 | 43525_2#101 | ERR10854260 |
| 6623STDY11015461 | 43525_2#105 | ERR10854264 |
| 6623STDY11015462 | 43525_2#107 | ERR10854266 |
| 6623STDY11015463 | 43525_2#109 | ERR10854268 |
| 6623STDY11015464 | 43525_2#111 | ERR10854270 |
| 6623STDY11015466 | 43525_2#131 | ERR10854274 |
| 6623STDY11015467 | 43525_2#133 | ERR10854276 |
| 6623STDY11015468 | 43525_2#135 | ERR10854278 |
| 6623STDY11015469 | 43525_2#137 | ERR10854280 |
| 6623STDY11015470 | 43525_2#139 | ERR10854282 |
| 6623STDY11015471 | 43525_2#141 | ERR10854284 |
| 6623STDY11015473 | 43525_2#161 | ERR10854288 |
| 6623STDY11015474 | 43525_2#163 | ERR10854290 |
| 6623STDY11015477 | 43525_2#169 | ERR10854296 |
| 6623STDY11015478 | 43525_2#171 | ERR10854298 |
| 6623STDY11015479 | 43525_2#173 | ERR10854300 |
| 6623STDY11015481 | 43525_2#192 | ERR10854304 |
| 6623STDY11015482 | 43525_2#194 | ERR10854306 |
| 6623STDY11015484 | 43525_2#198 | ERR10854310 |
| 6623STDY11015485 | 43525_2#200 | ERR10854312 |
| 6623STDY11015486 | 43525_2#202 | ERR10854314 |
| 6623STDY11015487 | 43525_2#204 | ERR10854315 |
| 6623STDY11015488 | 43525_2#206 | ERR10854316 |
| 6623STDY11015505 | 43525_2#264 | ERR10854333 |
| 6623STDY11015507 | 43525_2#268 | ERR10854335 |
| 6623STDY11015508 | 43525_2#270 | ERR10854336 |
| 6623STDY11015509 | 43525_2#272 | ERR10854337 |
| 6623STDY11015512 | 43525_2#278 | ERR10854340 |
| 6623STDY11015516 | 43525_2#294 | ERR10854344 |
| 6623STDY11015517 | 43525_2#296 | ERR10854345 |
| 6623STDY11015518 | 43525_2#298 | ERR10854346 |
| 6623STDY11015523 | 43525_2#316 | ERR10854351 |
| 6623STDY11015524 | 43525_2#318 | ERR10854352 |
| 6623STDY11015527 | 43525_2#324 | ERR10854355 |
| 6623STDY11015528 | 43525_2#326 | ERR10854356 |
| 6623STDY11015529 | 43525_2#2 | ERR10854208 |
| 6623STDY11015530 | 43525_2#4 | ERR10854210 |
| 6623STDY11015531 | 43525_2#6 | ERR10854212 |
| 6623STDY11015533 | 43525_2#10 | ERR10854216 |
| 6623STDY11015534 | 43525_2#12 | ERR10854218 |
| 6623STDY11015536 | 43525_2#16 | ERR10854222 |
| 6623STDY11015538 | 43525_2#36 | ERR10854226 |
| 6623STDY11015539 | 43525_2#38 | ERR10854228 |
| 6623STDY11015540 | 43525_2#40 | ERR10854230 |
| 6623STDY11015541 | 43525_2#42 | ERR10854232 |
| 6623STDY11015542 | 43525_2#44 | ERR10854235 |
| 6623STDY11015543 | 43525_2#46 | ERR10854237 |
| 6623STDY11015544 | 43525_2#48 | ERR10854239 |
| 6623STDY11015545 | 43525_2#66 | ERR10854241 |
| 6623STDY11015546 | 43525_2#68 | ERR10854243 |
| 6623STDY11015548 | 43525_2#72 | ERR10854247 |
| 6623STDY11015549 | 43525_2#74 | ERR10854249 |
| 6623STDY11015552 | 43525_2#80 | ERR10854255 |
| 6623STDY11015553 | 43525_2#98 | ERR10854257 |
| 6623STDY11015554 | 43525_2#100 | ERR10854259 |
| 6623STDY11015571 | 43525_2#166 | ERR10854293 |
| 6623STDY11015572 | 43525_2#168 | ERR10854295 |
| 6623STDY11015573 | 43525_2#170 | ERR10854297 |
| 6623STDY11015574 | 43525_2#172 | ERR10854299 |
| 6623STDY11015575 | 43525_2#174 | ERR10854301 |
| 6623STDY11015576 | 43525_2#176 | ERR10854303 |
| 6623STDY11015577 | 43525_2#193 | ERR10854305 |
| 6623STDY11015625 | 47858_2#2 | ERR13709752 |
| 6623STDY11015626 | 47858_2#4 | ERR13709754 |
| 6623STDY11015627 | 47858_2#6 | ERR13709756 |
| 6623STDY11015629 | 47858_2#10 | ERR13709758 |
| 6623STDY11015630 | 47858_2#12 | ERR13709759 |
| 6623STDY11015631 | 47858_2#14 | ERR13709760 |
| 6623STDY11015632 | 47858_2#16 | ERR13709761 |
| 6623STDY11015633 | 47858_2#34 | ERR13709778 |
| 6623STDY11015634 | 47858_2#36 | ERR13709779 |
| 6623STDY11015635 | 47858_2#38 | ERR13709780 |
| 6623STDY11015636 | 47858_2#40 | ERR13709781 |
| 6623STDY11015637 | 47858_2#42 | ERR13709782 |
| 6623STDY11015638 | 47858_2#44 | ERR13709783 |
| 6623STDY11015639 | 47858_2#46 | ERR13709784 |
| 6623STDY11015640 | 47858_2#48 | ERR13709785 |
| 6623STDY11015641 | 47858_2#66 | ERR13709802 |
| 6623STDY11015642 | 47858_2#68 | ERR13709803 |
| 6623STDY11015643 | 47858_2#70 | ERR13709804 |
| 6623STDY11015644 | 47858_2#72 | ERR13709805 |
| 6623STDY11015645 | 47858_2#74 | ERR13709806 |
| 6623STDY11015646 | 47858_2#76 | ERR13709807 |
| 6623STDY11015647 | 47858_2#78 | ERR13709808 |
| 6623STDY11015648 | 47858_2#80 | ERR13709809 |
| 6623STDY11015649 | 47858_2#98 | ERR13709826 |
| 6623STDY11015650 | 47858_2#100 | ERR13709827 |
| 6623STDY11015651 | 47858_2#102 | ERR13709828 |
| 6623STDY11015652 | 47858_2#104 | ERR13709829 |
| 6623STDY11015653 | 47858_2#106 | ERR13709830 |
| 6623STDY11015654 | 47858_2#108 | ERR13709831 |
| 6623STDY11015655 | 47858_2#110 | ERR13709832 |
| 6623STDY11015656 | 47858_2#112 | ERR13709833 |
| 6623STDY11015657 | 47858_2#130 | ERR13709850 |
| 6623STDY11015658 | 47858_2#132 | ERR13709851 |
| 6623STDY11015659 | 47858_2#134 | ERR13709852 |
| 6623STDY11015660 | 47858_2#136 | ERR13709853 |
| 6623STDY11015661 | 47858_2#138 | ERR13709854 |
| 6623STDY11015662 | 47858_2#140 | ERR13709855 |
| 6623STDY11015663 | 47858_2#142 | ERR13709856 |
| 6623STDY11015664 | 47858_2#144 | ERR13709857 |
| 6623STDY11015665 | 47858_2#162 | ERR13709874 |
| 6623STDY11015666 | 47858_2#164 | ERR13709875 |
| 6623STDY11015667 | 47858_2#166 | ERR13709876 |
| 6623STDY11015668 | 47858_2#168 | ERR13709877 |
| 6623STDY11015669 | 47858_2#170 | ERR13709878 |
| 6623STDY11015670 | 47858_2#172 | ERR13709879 |
| 6623STDY11015671 | 47858_2#174 | ERR13709880 |
| 6623STDY11015672 | 47858_2#176 | ERR13709881 |
| 6623STDY11015673 | 47858_2#194 | ERR13709898 |
| 6623STDY11015674 | 47858_2#196 | ERR13709899 |
| 6623STDY11015675 | 47858_2#198 | ERR13709900 |
| 6623STDY11015676 | 47858_2#200 | ERR13709901 |
| 6623STDY11015677 | 47858_2#202 | ERR13709902 |
| 6623STDY11015678 | 47858_2#204 | ERR13709903 |
| 6623STDY11015679 | 47858_2#206 | ERR13709904 |
| 6623STDY11015681 | 47858_2#226 | ERR13709922 |
| 6623STDY11015682 | 47858_2#228 | ERR13709923 |
| 6623STDY11015683 | 47858_2#230 | ERR13709924 |
| 6623STDY11015684 | 47858_2#232 | ERR13709925 |
| 6623STDY11015685 | 47858_2#234 | ERR13709926 |
| 6623STDY11015686 | 47858_2#236 | ERR13709927 |
| 6623STDY11015687 | 47858_2#238 | ERR13709928 |
| 6623STDY11015688 | 47858_2#240 | ERR13709929 |
| 6623STDY11015689 | 47858_2#258 | ERR13709946 |
| 6623STDY11015690 | 47858_2#260 | ERR13709947 |
| 6623STDY11015701 | 47858_2#298 | ERR13709974 |
| 6623STDY11015703 | 47858_2#302 | ERR13709976 |
| 6623STDY11015704 | 47858_2#304 | ERR13709977 |
| 6623STDY11015705 | 47858_2#322 | ERR13709994 |
| 6623STDY11015706 | 47858_2#324 | ERR13709995 |
| 6623STDY11015707 | 47858_2#326 | ERR13709996 |
| 6623STDY11015708 | 47858_2#328 | ERR13709997 |
| 6623STDY11015710 | 47858_2#332 | ERR13709999 |
| 6623STDY11015711 | 47858_2#334 | ERR13710000 |
| 6623STDY11015712 | 47858_2#336 | ERR13710001 |
| 6623STDY11015713 | 47858_2#354 | ERR13710018 |
| 6623STDY11015714 | 47858_2#356 | ERR13710019 |
| 6623STDY11015715 | 47858_2#358 | ERR13710020 |
| 6623STDY11015716 | 47858_2#360 | ERR13710021 |
| 6623STDY11015717 | 47858_2#362 | ERR13710022 |
| 6623STDY11015718 | 47858_2#364 | ERR13710023 |
| 6623STDY11015719 | 47858_2#366 | ERR13710024 |
| 6623STDY11015720 | 47858_2#367 | ERR13710025 |
| 6623STDY11015723 | 47858_2#21 | ERR13709766 |
| 6623STDY11015724 | 47858_2#23 | ERR13709768 |
| 6623STDY11015725 | 47858_2#25 | ERR13709770 |
| 6623STDY11015727 | 47858_2#29 | ERR13709774 |
| 6623STDY11015728 | 47858_2#31 | ERR13709776 |
| 6623STDY11015729 | 47858_2#49 | ERR13709786 |
| 6623STDY11015730 | 47858_2#51 | ERR13709788 |
| 6623STDY11015731 | 47858_2#53 | ERR13709790 |
| 6623STDY11015732 | 47858_2#55 | ERR13709792 |
| 6623STDY11015733 | 47858_2#57 | ERR13709794 |
| 6623STDY11015734 | 47858_2#59 | ERR13709796 |
| 6623STDY11015735 | 47858_2#61 | ERR13709798 |
| 6623STDY11015737 | 47858_2#81 | ERR13709810 |
| 6623STDY11015738 | 47858_2#83 | ERR13709812 |
| 6623STDY11015739 | 47858_2#85 | ERR13709814 |
| 6623STDY11015740 | 47858_2#87 | ERR13709816 |
| 6623STDY11015741 | 47858_2#89 | ERR13709818 |
| 6623STDY11015742 | 47858_2#91 | ERR13709820 |
| 6623STDY11015743 | 47858_2#93 | ERR13709822 |
| 6623STDY11015744 | 47858_2#95 | ERR13709824 |
| 6623STDY11015745 | 47858_2#113 | ERR13709834 |
| 6623STDY11015746 | 47858_2#115 | ERR13709836 |
| 6623STDY11015747 | 47858_2#117 | ERR13709838 |
| 6623STDY11015748 | 47858_2#119 | ERR13709840 |
| 6623STDY11015750 | 47858_2#123 | ERR13709844 |
| 6623STDY11015751 | 47858_2#125 | ERR13709846 |
| 6623STDY11015752 | 47858_2#127 | ERR13709848 |
| 6623STDY11015753 | 47858_2#145 | ERR13709858 |
| 6623STDY11015754 | 47858_2#147 | ERR13709860 |
| 6623STDY11015755 | 47858_2#149 | ERR13709862 |
| 6623STDY11015756 | 47858_2#151 | ERR13709864 |
| 6623STDY11015757 | 47858_2#153 | ERR13709866 |
| 6623STDY11015758 | 47858_2#155 | ERR13709868 |
| 6623STDY11015759 | 47858_2#157 | ERR13709870 |
| 6623STDY11015760 | 47858_2#159 | ERR13709872 |
| 6623STDY11015761 | 47858_2#177 | ERR13709882 |
| 6623STDY11015762 | 47858_2#179 | ERR13709884 |
| 6623STDY11015763 | 47858_2#181 | ERR13709886 |
| 6623STDY11015767 | 47858_2#189 | ERR13709894 |
| 6623STDY11015768 | 47858_2#191 | ERR13709896 |
| 6623STDY11015773 | 47858_2#217 | ERR13709914 |
| 6623STDY11015774 | 47858_2#219 | ERR13709916 |
| 6623STDY11015775 | 47858_2#221 | ERR13709918 |
| 6623STDY11015776 | 47858_2#223 | ERR13709920 |
| 6623STDY11015777 | 47858_2#241 | ERR13709930 |
| 6623STDY11015778 | 47858_2#243 | ERR13709932 |
| 6623STDY11015779 | 47858_2#245 | ERR13709934 |
| 6623STDY11015780 | 47858_2#247 | ERR13709936 |
| 6623STDY11015781 | 47858_2#249 | ERR13709938 |
| 6623STDY11015782 | 47858_2#251 | ERR13709940 |
| 6623STDY11015783 | 47858_2#253 | ERR13709942 |
| 6623STDY11015784 | 47858_2#255 | ERR13709944 |
| 6623STDY11015787 | 47858_2#277 | ERR13709958 |
| 6623STDY11015788 | 47858_2#279 | ERR13709960 |
| 6623STDY11015789 | 47858_2#281 | ERR13709962 |
| 6623STDY11015790 | 47858_2#283 | ERR13709964 |
| 6623STDY11015791 | 47858_2#285 | ERR13709966 |
| 6623STDY11015793 | 47858_2#305 | ERR13709978 |
| 6623STDY11015794 | 47858_2#307 | ERR13709980 |
| 6623STDY11015795 | 47858_2#309 | ERR13709982 |
| 6623STDY11015796 | 47858_2#311 | ERR13709984 |
| 6623STDY11015797 | 47858_2#313 | ERR13709986 |
| 6623STDY11015798 | 47858_2#315 | ERR13709988 |
| 6623STDY11015799 | 47858_2#317 | ERR13709990 |
| 6623STDY11015800 | 47858_2#319 | ERR13709992 |
| 6623STDY11015801 | 47858_2#337 | ERR13710002 |
| 6623STDY11015802 | 47858_2#339 | ERR13710004 |
| 6623STDY11015803 | 47858_2#341 | ERR13710006 |
| 6623STDY11015804 | 47858_2#343 | ERR13710008 |
| 6623STDY11015805 | 47858_2#345 | ERR13710010 |
| 6623STDY11015806 | 47858_2#347 | ERR13710012 |
| 6623STDY11015807 | 47858_2#349 | ERR13710014 |
| 6623STDY11015808 | 47858_2#351 | ERR13710016 |
| 6623STDY11015809 | 47858_2#368 | ERR13710026 |
| 6623STDY11015810 | 47858_2#370 | ERR13710028 |
| 6623STDY11015811 | 47858_2#372 | ERR13710030 |
| 6623STDY11015812 | 47858_2#374 | ERR13710032 |
| 6623STDY11015813 | 47858_2#376 | ERR13710034 |
| 6623STDY11015814 | 47858_2#378 | ERR13710036 |
| 6623STDY11015815 | 47858_2#380 | ERR13710038 |
| 6623STDY11015816 | 47858_2#382 | ERR13710040 |
| 6623STDY11015817 | 47858_2#18 | ERR13709763 |
| 6623STDY11015818 | 47858_2#20 | ERR13709765 |
| 6623STDY11015819 | 47858_2#22 | ERR13709767 |
| 6623STDY11015820 | 47858_2#24 | ERR13709769 |
| 6623STDY11015821 | 47858_2#26 | ERR13709771 |
| 6623STDY11015823 | 47858_2#30 | ERR13709775 |
| 6623STDY11015824 | 47858_2#32 | ERR13709777 |
| 6623STDY11015825 | 47858_2#50 | ERR13709787 |
| 6623STDY11015826 | 47858_2#52 | ERR13709789 |
| 6623STDY11015827 | 47858_2#54 | ERR13709791 |
| 6623STDY11015828 | 47858_2#56 | ERR13709793 |
| 6623STDY11015829 | 47858_2#58 | ERR13709795 |
| 6623STDY11015830 | 47858_2#60 | ERR13709797 |
| 6623STDY11015831 | 47858_2#62 | ERR13709799 |
| 6623STDY11015832 | 47858_2#64 | ERR13709801 |
| 6623STDY11015833 | 47858_2#82 | ERR13709811 |
| 6623STDY11015834 | 47858_2#84 | ERR13709813 |
| 6623STDY11015835 | 47858_2#86 | ERR13709815 |
| 6623STDY11015836 | 47858_2#88 | ERR13709817 |
| 6623STDY11015837 | 47858_2#90 | ERR13709819 |
| 6623STDY11015838 | 47858_2#92 | ERR13709821 |
| 6623STDY11015839 | 47858_2#94 | ERR13709823 |
| 6623STDY11015840 | 47858_2#96 | ERR13709825 |
| 6623STDY11015841 | 47858_2#114 | ERR13709835 |
| 6623STDY11015842 | 47858_2#116 | ERR13709837 |
| 6623STDY11015843 | 47858_2#118 | ERR13709839 |
| 6623STDY11015844 | 47858_2#120 | ERR13709841 |
| 6623STDY11015845 | 47858_2#122 | ERR13709843 |
| 6623STDY11015846 | 47858_2#124 | ERR13709845 |
| 6623STDY11015847 | 47858_2#126 | ERR13709847 |
| 6623STDY11015848 | 47858_2#128 | ERR13709849 |
| 6623STDY11015849 | 47858_2#146 | ERR13709859 |
| 6623STDY11015850 | 47858_2#148 | ERR13709861 |
| 6623STDY11015851 | 47858_2#150 | ERR13709863 |
| 6623STDY11015852 | 47858_2#152 | ERR13709865 |
| 6623STDY11015853 | 47858_2#154 | ERR13709867 |
| 6623STDY11015854 | 47858_2#156 | ERR13709869 |
| 6623STDY11015855 | 47858_2#158 | ERR13709871 |
| 6623STDY11015856 | 47858_2#160 | ERR13709873 |
| 6623STDY11015857 | 47858_2#178 | ERR13709883 |
| 6623STDY11015858 | 47858_2#180 | ERR13709885 |
| 6623STDY11015859 | 47858_2#182 | ERR13709887 |
| 6623STDY11015860 | 47858_2#184 | ERR13709889 |
| 6623STDY11015861 | 47858_2#186 | ERR13709891 |
| 6623STDY11015862 | 47858_2#188 | ERR13709893 |
| 6623STDY11015863 | 47858_2#190 | ERR13709895 |
| 6623STDY11015864 | 47858_2#192 | ERR13709897 |
| 6623STDY11015865 | 47858_2#210 | ERR13709907 |
| 6623STDY11015867 | 47858_2#214 | ERR13709911 |
| 6623STDY11015868 | 47858_2#216 | ERR13709913 |
| 7911STDY15470511 | 50821_1#73 | ERR16025358 |
| 7911STDY15470517 | 50821_1#93 | ERR16025366 |
| 7911STDY15470518 | 50821_1#95 | ERR16025367 |
| 7911STDY15470519 | 50821_1#97 | ERR16025368 |
| 7911STDY15470526 | 50821_1#119 | ERR16025376 |
| 7911STDY15470527 | 50821_1#121 | ERR16025377 |
| 7911STDY15470531 | 50821_1#127 | ERR16025378 |
| 7911STDY15470534 | 50821_1#141 | ERR16025387 |
| 7911STDY15470535 | 50821_1#143 | ERR16025388 |
| 7911STDY15470539 | 50821_1#151 | ERR16025389 |
| 7911STDY15470542 | 50821_1#165 | ERR16025398 |
| 7911STDY15470543 | 50821_1#167 | ERR16025399 |
| 7911STDY15470546 | 50821_1#173 | ERR16025400 |
| 7911STDY15470550 | 50821_1#189 | ERR16025409 |
| 7911STDY15470551 | 50821_1#65 | ERR16025357 |
| 7911STDY15470554 | 50821_1#195 | ERR16025410 |
| 7911STDY15470557 | 50821_1#209 | ERR16025419 |
| 7911STDY15470558 | 50821_1#211 | ERR16025420 |
| 7911STDY15470559 | 50821_1#213 | ERR16025421 |
| 7911STDY15470562 | 50821_1#217 | ERR16025422 |
| 7911STDY15470565 | 50821_1#223 | ERR16025423 |
| 7911STDY15470566 | 50821_1#233 | ERR16025432 |
| 7911STDY15470567 | 50821_1#235 | ERR16025433 |
| 7911STDY15470570 | 50821_1#241 | ERR16025434 |
| 7911STDY15470571 | 50821_1#243 | ERR16025435 |
| 7911STDY15470573 | 50821_1#247 | ERR16025436 |
| 7911STDY15470574 | 50821_1#257 | ERR16025445 |
| 7911STDY15470577 | 50821_1#261 | ERR16025446 |
| 7911STDY15470578 | 50821_1#263 | ERR16025447 |
| 7911STDY15470579 | 50821_1#265 | ERR16025448 |
| 7911STDY15470581 | 50821_1#269 | ERR16025449 |
| 7911STDY15470582 | 50821_1#271 | ERR16025450 |
| 7911STDY15470585 | 50821_1#285 | ERR16025459 |
| 7911STDY15470586 | 50821_1#287 | ERR16025460 |
| 7911STDY15470589 | 50821_1#293 | ERR16025461 |
| 7911STDY15470590 | 50821_1#295 | ERR16025462 |
| 7911STDY15470593 | 50821_1#19 | ERR16025335 |
| 7911STDY15470597 | 50821_1#27 | ERR16025340 |
| 7911STDY15470598 | 50821_1#29 | ERR16025342 |
| 7911STDY15470601 | 50821_1#51 | ERR16025346 |
| 7911STDY15470604 | 50821_1#57 | ERR16025350 |
| 7911STDY15470605 | 50821_1#59 | ERR16025352 |
| 7911STDY15470606 | 50821_1#61 | ERR16025354 |
| 7911STDY15470607 | 50821_1#63 | ERR16025356 |
| 7911STDY15470608 | 50821_1#80 | ERR16025359 |
| 7911STDY15470609 | 50821_1#81 | ERR16025360 |
| 7911STDY15470611 | 50821_1#83 | ERR16025361 |
| 7911STDY15470612 | 50821_1#84 | ERR16025362 |
| 7911STDY15470613 | 50821_1#85 | ERR16025363 |
| 7911STDY15470614 | 50821_1#86 | ERR16025364 |
| 7911STDY15470615 | 50821_1#87 | ERR16025365 |
| 7911STDY15470616 | 50821_1#104 | ERR16025369 |
| 7911STDY15470617 | 50821_1#105 | ERR16025370 |
| 7911STDY15470619 | 50821_1#107 | ERR16025371 |
| 7911STDY15470620 | 50821_1#108 | ERR16025372 |
| 7911STDY15470621 | 50821_1#109 | ERR16025373 |
| 7911STDY15470622 | 50821_1#110 | ERR16025374 |
| 7911STDY15470623 | 50821_1#111 | ERR16025375 |
| 7911STDY15470624 | 50821_1#128 | ERR16025379 |
| 7911STDY15470625 | 50821_1#129 | ERR16025380 |
| 7911STDY15470626 | 50821_1#130 | ERR16025381 |
| 7911STDY15470627 | 50821_1#131 | ERR16025382 |
| 7911STDY15470628 | 50821_1#132 | ERR16025383 |
| 7911STDY15470629 | 50821_1#133 | ERR16025384 |
| 7911STDY15470630 | 50821_1#134 | ERR16025385 |
| 7911STDY15470631 | 50821_1#135 | ERR16025386 |
| 7911STDY15470632 | 50821_1#152 | ERR16025390 |
| 7911STDY15470633 | 50821_1#153 | ERR16025391 |
| 7911STDY15470634 | 50821_1#154 | ERR16025392 |
| 7911STDY15470635 | 50821_1#155 | ERR16025393 |
| 7911STDY15470636 | 50821_1#156 | ERR16025394 |
| 7911STDY15470637 | 50821_1#157 | ERR16025395 |
| 7911STDY15470638 | 50821_1#158 | ERR16025396 |
| 7911STDY15470639 | 50821_1#159 | ERR16025397 |
| 7911STDY15470640 | 50821_1#176 | ERR16025401 |
| 7911STDY15470641 | 50821_1#177 | ERR16025402 |
| 7911STDY15470642 | 50821_1#178 | ERR16025403 |
| 7911STDY15470643 | 50821_1#179 | ERR16025404 |
| 7911STDY15470644 | 50821_1#180 | ERR16025405 |
| 7911STDY15470645 | 50821_1#181 | ERR16025406 |
| 7911STDY15470646 | 50821_1#182 | ERR16025407 |
| 7911STDY15470647 | 50821_1#183 | ERR16025408 |
| 7911STDY15470648 | 50821_1#200 | ERR16025411 |
| 7911STDY15470649 | 50821_1#201 | ERR16025412 |
| 7911STDY15470650 | 50821_1#202 | ERR16025413 |
| 7911STDY15470651 | 50821_1#203 | ERR16025414 |
| 7911STDY15470652 | 50821_1#204 | ERR16025415 |
| 7911STDY15470653 | 50821_1#205 | ERR16025416 |
| 7911STDY15470654 | 50821_1#206 | ERR16025417 |
| 7911STDY15470655 | 50821_1#207 | ERR16025418 |
| 7911STDY15470656 | 50821_1#224 | ERR16025424 |
| 7911STDY15470657 | 50821_1#225 | ERR16025425 |
| 7911STDY15470658 | 50821_1#226 | ERR16025426 |
| 7911STDY15470659 | 50821_1#227 | ERR16025427 |
| 7911STDY15470660 | 50821_1#228 | ERR16025428 |
| 7911STDY15470661 | 50821_1#229 | ERR16025429 |
| 7911STDY15470662 | 50821_1#230 | ERR16025430 |
| 7911STDY15470663 | 50821_1#231 | ERR16025431 |
| 7911STDY15470664 | 50821_1#248 | ERR16025437 |
| 7911STDY15470665 | 50821_1#249 | ERR16025438 |
| 7911STDY15470666 | 50821_1#250 | ERR16025439 |
| 7911STDY15470667 | 50821_1#251 | ERR16025440 |
| 7911STDY15470668 | 50821_1#252 | ERR16025441 |
| 7911STDY15470669 | 50821_1#253 | ERR16025442 |
| 7911STDY15470670 | 50821_1#254 | ERR16025443 |
| 7911STDY15470671 | 50821_1#255 | ERR16025444 |
| 7911STDY15470672 | 50821_1#272 | ERR16025451 |
| 7911STDY15470673 | 50821_1#273 | ERR16025452 |
| 7911STDY15470674 | 50821_1#274 | ERR16025453 |
| 7911STDY15470675 | 50821_1#275 | ERR16025454 |
| 7911STDY15470676 | 50821_1#276 | ERR16025455 |
| 7911STDY15470677 | 50821_1#277 | ERR16025456 |
| 7911STDY15470678 | 50821_1#278 | ERR16025457 |
| 7911STDY15470679 | 50821_1#279 | ERR16025458 |
| 7911STDY15470680 | 50821_1#296 | ERR16025463 |
| 7911STDY15470681 | 50821_1#297 | ERR16025464 |
| 7911STDY15470682 | 50821_1#298 | ERR16025465 |
| 7911STDY15470683 | 50821_1#299 | ERR16025466 |
| 7911STDY15470684 | 50821_1#300 | ERR16025467 |
| 7911STDY15470685 | 50821_1#301 | ERR16025468 |
| 7911STDY15470686 | 50821_1#302 | ERR16025469 |
| 7911STDY15470687 | 50821_1#303 | ERR16025470 |
| 7911STDY15470688 | 50821_1#18 | ERR16025334 |
| 7911STDY15470689 | 50821_1#20 | ERR16025336 |
| 7911STDY15470690 | 50821_1#22 | ERR16025337 |
| 7911STDY15470691 | 50821_1#24 | ERR16025338 |
| 7911STDY15470692 | 50821_1#26 | ERR16025339 |
| 7911STDY15470693 | 50821_1#28 | ERR16025341 |
| 7911STDY15470694 | 50821_1#30 | ERR16025343 |
| 7911STDY15470695 | 50821_1#32 | ERR16025344 |
| 7911STDY15470696 | 50821_1#50 | ERR16025345 |
| 7911STDY15470697 | 50821_1#52 | ERR16025347 |
| 7911STDY15470698 | 50821_1#54 | ERR16025348 |
| 7911STDY15470699 | 50821_1#56 | ERR16025349 |
| 7911STDY15470700 | 50821_1#58 | ERR16025351 |
| 7911STDY15470701 | 50821_1#60 | ERR16025353 |
| 7911STDY15470702 | 50821_1#62 | ERR16025355 |
