# Supplementary material for: Group B streptococcal disease in infants 0–3 months in The Netherlands, 1987–2023: a nationwide genomic and epidemiological surveillance study
Source: Lancet Reg Health Eur. 2026 May 12;66:101711. doi: 10.1016/j.lanepe.2026.101711 (PMC13193784; doi:10.1016/j.lanepe.2026.101711)
Supplement: Collab Authors [file mmc2.docx]

**GROUP B STREPTOCOCCAL DISEASE IN INFANTS 0-3 MONTHS IN THE NETHERLANDS, 1987-2023: A NATIONWIDE GENOMIC AND EPIDEMIOLOGICAL SURVEILLANCE STUDY**

**NOGBS study group:**

| **First name** | **Surname** | **Affiliation** |
| --- | --- | --- |
| Ron | van Beek | Department of Paediatrics, Amphia Hospital, Breda, the Netherlands |
| Vincent | Bekker | Department of Paediatrics, Leiden University Medical Centre, Leiden, the Netherlands |
| Maartje | van den Berg | Department of Paediatrics, Haaglanden Medical Centre, The Hague, the Netherlands |
| Geert Jan | Blok | Department of Neonatology, Northwest Clinics, Alkmaar, the Netherlands |
| Mijke | Breukels | Department of Paediatrics, Elkerliek Hospital, Helmond, the Netherlands |
| Alwin F.J. | Brouwer | Department of Paediatrics, Hospital of Nij Smellinghe, Drachten, the Netherlands |
| Renske J.P.M. | Cornelisse-van Vugt | Department of Paediatrics, Canisius Wilhelmina Hospital, Nijmegen, the Netherlands |
| Luçan C. | Delemarre | Department of Paediatrics, Amstelland Hospital, Amstelveen, the Netherlands |
| Anouk | Dings | Department of Paediatrics, Gelre Hospitals, Apeldoorn, the Netherlands |
| Rienus A | Doedens | Department of Paediatrics, Martini Hospital, Groningen, the Netherlands |
| Stefan M. | van Dorth | Department of Paediatrics, Tjongerschans Hospital, Heerenveen, the Netherlands |
| Hester M. | Havers | Department of Paediatrics, Alrijne Hospital, Leiderdorp, the Netherlands |
| Jojanneke | Heidema | Department of Paediatrics, Antonius Hospital, Utrecht, the Netherlands |
| Marieke A.C. | Hemels | Department of Neonatology, Isala Clinics, Zwolle, the Netherlands |
| Maartje E.N. | van den Heuvel | Department of Paediatrics, OLVG, location West, Amsterdam, the Netherlands |
| Marlies A. | van Houten | Department of Infectious Diseases (LUCID), Leiden University Medical Centre, Leiden, the Netherlands |
| Monique A.M. | Jacobs | Department of Paediatrics, Slingeland Hospital, Doetinchem, the Netherlands |
| Arieke | Janse | Department of Paediatrics, Gelderse Vallei Hospital, Ede, the Netherlands |
| Miranda | de Jong | Department of Paediatrics, Albert Schweitzer Hospital, Dordrecht, the Netherlands |
| Anton H. | van Kaam | Department of Neonatology, Emma Children’s Hospital, Amsterdam UMC, Amsterdam, the Netherlands |
| Ageeth | Kaspers | Department of Paediatrics, Medisch Spectrum Twente, Twente, the Netherlands |
| Merel N. | van Kassel | Department of Anaesthesiology, Amsterdam UMC, Amsterdam, the Netherlands |
| Anne A.M.W. | van Kempen | Department of Paediatrics, OLVG, location East, Amsterdam, the Netherlands |
| Kristine | Klúčovská | Department of Paediatrics, Treant Hospital Group, Hoogeveen, the Netherlands |
| Karen | Korbeek | Department of Paediatrics, St. Jansdal Hospital, Harderwijk, the Netherlands |
| René F. | Kornelisse | Department of Paediatrics, Erasmus Medical Centre, Rotterdam, the Netherlands |
| Anke G. | Kuijpers | Department of Paediatrics, Bernhoven Hospital, Uden, the Netherlands |
| Taco W. | Kuijpers | Department of Peadiatrics, Emma Children’s Hospital, Amsterdam UMC, Amsterdam, the Netherlands |
| Elizabeth | van Leeuwen | Department of Obstetrics and Gynaecology, Amsterdam UMC, Amsterdam, the Netherlands |
| Jeannette | von Lindern | Department of Paediatrics, Groene Hart Hospital, Gouda, the Netherlands |
| Carmen | Lorente Flores | Department of Neonatology, Maxima Medical Centre, Veldhoven, the Netherlands |
| Karen | Van Mechelen | Department of Neonatology, Maastricht University Medical Centre, Maastricht, the Netherlands |
| Clemens B. | Meijssen | Department of Paediatrics, Meander Medical Centre, Amersfoort, the Netherlands |
| Jeroen | Noordzij | Department of Paediatrics, Reinier de Graaf Hospital, Delft, the Netherlands |
| Annemarie | Oudshoorn | Department of Paediatrics, Gelre Hospitals, Apeldoorn, the Netherlands |
| Frans B. | Plötz | Department of Paediatrics, Tergooi Medical Centre, Hilversum, the Netherlands |
| Marjolijn | Quaak | Department of Paediatrics, Dijklander Hospital, Hoorn, the Netherlands |
| Maaike | van Rossem | Department of Paediatrics, Rijnstate Hospital, Arnhem, the Netherlands |
| Maarten | Rijpert | Department of Paediatrics, Zaans Medical Centre, Zaandam, the Netherlands |
| Machteld A.G. | van Scherpenzeel-de Vries | Department of Paediatrics, Frisius Medical Centre, Leeuwarden, the Netherlands |
| Irene | Schiering | Department of Paediatrics, Spaarne Gasthuis, Haarlem, the Netherlands |
| George | Shabo | Department of Paediatrics, Hospital Group Twente, Twente, the Netherlands |
| Linde | Snoek | Department of General Practice, Amsterdam UMC, Amsterdam, the Netherlands |
| Nina M. | van Sorge | Department of Medical Microbiology and Infection Prevention, Amsterdam UMC, Amsterdam, the Netherlands |
| Jacqueline U.M. | Termote | Department of Neonatology, University Medical Centre Utrecht, Utrecht, the Netherlands |
| Gerdien A. | Tramper-Stranders | Department of Paediatrics, Franciscus Gasthuis, Rotterdam, the Netherlands |
| Mirjam | van Veen | Department of Paediatrics, Haga Hospital, The Hague, the Netherlands |
| Marlies | Vermaas | Department of Paediatrics, Admiraal de Ruyter Hospital, Goes, the Netherlands |
| Marjoke | Verweij | Department of Paediatrics, Viecuri Medical Centre, Venlo, the Netherlands |
| Douwe H. | Visser | Department of Neonatology, Emma Children’s Hospital, Amsterdam UMC, Amsterdam, the Netherlands |
| Wouter J. | de Waal | Department of Paediatrics, Diakonesse Hospital, Utrecht, the Netherlands |
| Anne-Marie | van Wermeskerken | Department of Paediatrics, Flevohospital, Almere, the Netherlands |
| Janneke F. | Wilms | Department of Paediatrics, BovenIJ Hospital, Amsterdam, the Netherlands |
| Tom F.W | Wolfs | Department of Paediatrics, University Medical Centre Utrecht, Utrecht, the Netherlands |
| Angela C.M. | van Zijl | Department of Neonatology, University Medical Centre Utrecht, Utrecht, the Netherlands |
